# NFIA-dependent upregulation of SMC4 promotes metastasis and metabolic reprogramming in glioma

**DOI:** 10.3389/fonc.2025.1624370

**Published:** 2025-08-25

**Authors:** Guoyin Li, Yukui Zhao, Yubo He, Zhaoqiang Qian, Xiaoyan Li, Zewen Song, Zhiqiang Liu

**Affiliations:** ^1^ Key Laboratory of Modern Teaching Technology, Ministry of Education, Shaanxi Normal University, Xi’an, China; ^2^ Fuxi Laboratory, Zhoukou Normal University, Zhoukou, China; ^3^ Department of Neurosurgery, Shanxi Provincial People’s Hospital, Taiyuan, China; ^4^ College of Life Sciences, Shaanxi Normal University, Xi’an, China; ^5^ Department of Blood Transfusion, Shanxi Provincial People’s Hospital, Taiyuan, China; ^6^ Department of Oncology, The Third Xiangya Hospital of Central South University, Changsha, China

**Keywords:** glioma, NFIA/SMC4 axis, metastasis, metabolic reprogramming, SRRs

## Abstract

**Background:**

Gliomas, particularly glioblastoma, are aggressive brain tumors with poor prognosis and unmet therapeutic needs. Structural maintenance of chromosomes 4 (SMC4), a core component of the condensin complex, is dysregulated in multiple cancers, but its role in glioma metabolism and metastasis remains unclear.

**Methods:**

Using integrated multi-omics analyses of glioma datasets, we assessed SMC4 expression and its correlation with clinical outcomes. Functional studies in U-251MG and LN229 glioma cells including CCK-8, EdU, cell cycle, Transwell, and wound-healing assays were combined with subcutaneous xenograft and tail-vein metastasis mouse models to evaluate SMC4’s effects on proliferation, migration, invasion, and metastasis. ECAR/OCR and rescue experiments validated SMC4’s role in glycolysis. Luciferase reporter and ChIP assays identified nuclear factor I A (NFIA) as an upstream transcriptional regulator of SMC4. A prognostic model (SRRS) was developed via LASSO regression and validated across cohorts.

**Results:**

SMC4 was significantly overexpressed in glioma tissues, with higher expression correlating with advanced tumor grades and poorer patient survival (AUC > 0.82). Mechanistically, SMC4 promoted G1/S cell cycle transition and proliferation *in vitro*/*in vivo*. It enhanced metastasis by activating TGF-β/SMAD signaling, evidenced by upregulated p-SMAD2/3, N-cadherin, SNAI1, and ZEB1, and increased lung metastases in mice. SMC4 also facilitated aerobic glycolysis by upregulating LDHA, shown via increased glucose uptake, lactate production, and ECAR, with rescue experiments confirming LDHA dependency. NFIA directly bound two motifs in the SMC4 promoter (-1379 bp and -354 bp), driving transcription, validated by dual-luciferase and ChIP assays. The SRRS, integrating 15 SMC4-coexpressed genes, stratified patients into high/low-risk groups with distinct survival (AUC > 0.7 for 1-, 3-, 5-year OS). A nomogram combining SRRS and clinical parameters improved accuracy (AUC > 0.88). Pharmacogenomic analysis linked SRRS to sensitivity to erlotinib and other agents.

**Conclusion:**

SMC4 drives glioma progression through dual mechanisms TGF-β/SMAD-mediated metastasis and LDHA-dependent glycolysis regulated by NFIA. This extends beyond its known role in TGF-β activation by identifying NFIA as an upstream regulator and metabolic reprogramming as a novel function. The SRRS and nomogram provide robust tools for prognosis and personalized therapy, supporting the NFIA/SMC4 axis and downstream effectors as potential therapeutic targets for glioma.

## 1 Introduction

Glioma, a primary brain tumor originating from glial cells or their progenitor cells, constitutes approximately 25% of all primary brain neoplasms (1). Among them, glioblastoma (GBM), the most aggressive subtype, accounts for 14.3% of primary brain and central nervous system tumors and 49.1% of malignant brain tumors (1). GBM remains a devastating disease with a dismal 5-year survival rate of only 7.2% (2) and a median overall survival (OS) of 14.6 months post-diagnosis (3, 4). While low-grade gliomas (LGGs) exhibit more favorable prognoses with median OS of 78.1 months (WHO grade II) and 37.6 months (WHO grade III) (5), their long-term neurological sequelae and potential for malignant transformation necessitate improved therapeutic strategies (6). Current clinical challenges in glioma management include (1): incomplete surgical resection due to infiltrative growth patterns (2); intrinsic resistance to radiotherapy and chemotherapy (3); lack of validated molecular targets for precision therapy; and (4) high rates of recurrence and treatment-related morbidity. These unmet needs underscore the urgency of identifying novel therapeutic targets to improve patient outcomes.

Structural maintenance of chromosomes 4 (SMC4), a conserved ATPase belonging to the SMC family, is essential for chromosomal dynamics across eukaryotic species (7). This protein features a characteristic five-domain architecture: a hinge region mediating dimerization, N-terminal and C-terminal ATPase domains containing Walker A/B motifs for nucleotide binding (8, 9), and coiled-coil domains linking the terminal regions. Through its hinge domain, SMC4 forms a heterodimer with SMC2, which assembles into the cohesin complex with non-SMC subunits (10). This complex orchestrates critical nuclear processes, including sister chromatid cohesion, DNA replication, repair, and transcriptional regulation (11). Notably, SMC4’s ATPase activity serves as a molecular switch for condensin complex function (12), while emerging evidence implicates its roles in embryonic cell division, RNA splicing, and extracellular matrix remodeling (13).

Dysregulated SMC4 expression has been reported in multiple malignancies, including breast (14), cervical (15), endometrial (16), and hepatocellular carcinomas (17), with oncogenic roles attributed to activation of NF-κB (15), TGF-β/Smad (18), and JAK2/STAT3 (19) signaling pathways. In gliomas, SMC4 overexpression has been linked to tumor progression (18, 20), though its upstream regulatory mechanisms and functional implications in metabolic reprogramming remain poorly understood. Here, we demonstrate that Nuclear Factor I A (NFIA) transcriptionally upregulates SMC4 expression, and elucidate a dual mechanistic role for SMC4 in glioma progression: (1) promotion of metastatic potential via enhancement of TGF-β/Smad signaling transduction; and (2) facilitation of aerobic glycolysis through upregulation of LDHA. Additionally, we establish and validate an SMC4-centric prognostic model to stratify patient survival outcomes. Collectively, these findings deepen our understanding of glioma pathogenesis and highlight SMC4 as a multifunctional therapeutic target with translational potential for improved diagnosis and treatment.

## 2 Materials and methods

### 2.1 Data acquisition and processing

Tumor transcriptome and clinical datasets for LGG and GBM were obtained from The Cancer Genome Atlas (TCGA) database (https://portal.gdc.cancer.gov), including TCGA-LGG (normal (N) = 0, tumor (T) = 532) and TCGA-GBM cohorts (N = 5, T = 168). Chinese Glioma Genome Atlas (CGGA) datasets (CGGA_301, N = 0, T = 301; CGGA_325, N = 0, T = 325; CGGA_693, N = 0, T = 693) were downloaded from http://www.cgga.org.cn/, while GEO datasets (GSE4290, N = 27, T = 153; GSE29796, N = 20, T = 52; GSE50161, N = 13, T = 117) were retrieved from the Gene Expression Omnibus repository (https://ncbi.nlm.nih.gov/gds). Dataset selection: TCGA, CGGA, and GEO datasets were chosen for their large sample sizes, comprehensive clinical annotations (WHO grade, survival time), and inclusion of both low-grade glioma (LGG) and glioblastoma (GBM) samples to ensure generalizability. Raw RNA-seq data were processed using standard pipelines: normalization with DESeq2/limma, batch effect correction with sva, and filtering of low-expression genes (average counts < 10) to retain reliable transcripts. All datasets used in this study were derived from publicly accessible repositories, and thus, formal ethical approval was exempted per institutional guidelines.

### 2.2 Cell culture and lentiviral infection

Human glioma cell lines LN229 and U-251MG were acquired from the Cell Bank of the Shanghai Institute of Biological Sciences, Chinese Academy of Sciences. Both cell lines were maintained in Dulbecco’s Modified Eagle Medium (DMEM, high glucose) supplemented with 10% fetal bovine serum (FBS), under standard culture conditions of 37°C, 5% CO_2_, and 95% relative humidity. Stable cell lines with constitutive overexpression or knockdown of the target gene were generated using lentiviral vectors. Briefly, cells were transduced with lentiviral particles according to the manufacturer’s protocols, followed by selection with 2-5 μg/mL puromycin for 7 days to establish stable clones.

### 2.3 CCK-8 assay

U-251MG and LN229 cells were seeded into 96-well plates at a density of 1×10^4^ cells per well (approximately 15% confluence, n = 4) and allowed to adhere overnight. Cell viability was assessed at 0, 24, 48, and 72 hours post-seeding. The CCK-8 reagent (Beyotime, C0037, China) was prepared by diluting it 1:10 in serum-free DMEM. Subsequently, 100 μl of the diluted reagent was added to each well, and plates were incubated at 37°C for 1 hour. Absorbance values at 450 nm were measured using a microplate reader (BioTek, Synergy HTX) to quantify cell proliferation.

### 2.4 Cell cycle assay

U-251MG and LN229 cells were seeded into 6-well plates at a density of 2 × 10^5^ cells per well (approximately 20% confluence, n = 3) and incubated overnight to allow for cell attachment. Subsequently, the cells were starved in a medium supplemented with 1% FBS for 24 hours to synchronize the cell cycle. After starvation, the cells were cultured in complete medium (containing 10% FBS) for an additional 24 hours to resume normal growth. To analyze the cell cycle distribution, the cells were processed using a cell cycle assay kit (Life-iLab, AC12L544, China) according to the manufacturer’s instructions. Briefly, the cells were harvested, fixed, and stained with the provided reagents. The stained cells were then analyzed by flow cytometry (Agilent NovoCytes) to determine the proportion of cells in different phases of the cell cycle (G0/G1, S, and G2/M).

### 2.5 Glucose consumption assay

Glucose uptake in cell culture supernatants was measured using a Glucose Uptake Assay Kit (DOJINDO, UP20, Japan), following the manufacturer’s protocol. Briefly, LN229 cells (3×10^5^ cells/mL in MEM medium containing 10% FBS) were seeded at 150 μL per well in 96-well plates and allowed to adhere overnight at 37°C, 5% CO_2_ (n = 3). The next day, cell supernatants were removed, and cells were washed twice with 150 μL of pre-warmed (37°C) glucose-free, serum-free DMEM. After washing, 150 μL of glucose-free, serum-free DMEM was added to each well, and plates were incubated statically for 15 minutes under standard culture conditions to equilibrate the cells. Subsequently, supernatants were removed, and cells were treated with 150 μL of pre-warmed (37°C) probe solution (prepared with glucose-free, serum-free DMEM), and normally cultured for 15 minutes. Supernatants were discarded, and cells were washed three times with 150 μL of WI Solution, and detected with a fluorescence microplate reader (BioTek, Synergy HTX).

### 2.6 Lactate release assay

Lactate production was measured using a glycolysis assay kit (DOJINDO, G272, Japan) following the manufacturer’s instructions. A total of 1×10^4^ LN229 cells (10% FBS, 1% penicillin-streptomycin, MEM medium) were seeded into a black 96-well plate with a transparent bottom and incubated overnight at 37°C in a 5% CO2 incubator (n = 3). Remove the medium and add 100 µl of MEM medium containing 10µmol/l Carbonyl cyanide p-trifluoromethoxyphenyl hydrazone (FCCP) to each well. Incubate at 37°C in a 5% CO2 incubator for 4 hours. Transfer 50 µl of cell culture supernatant from each well into a 1.5ml microtube, and dilute it 10-fold with ultrapure water to prepare samples for lactate assay. Transfer 20µl of the diluted cell culture supernatant into each well of a new regular 96-well plate. Add 80 µl of the lactate working solution to each well. Incubate at 37°C in a 5% CO2 incubator for 30 minutes. Measure the absorbance at 450 nm using a microplate reader (BioTek, Synergy HTX), and compare the obtained absorbance values.

### 2.7 Extracellular acidification rate assay

Hydration of probes: Add Seahorse XF calibration solution to the Utility Plate. Place the test plate back onto the Utility Plate and hydrate the probes overnight in a 37°C incubator without CO2. ECAR detection solution: Take 100 ml of basic medium, preheat it at 37°C, add L-Glutamine to achieve a final concentration of 2 mM, and adjust the pH to 7.35 ± 0.05. Cells processing and detection: U-251MG cells were seeded into a Seahorse XF cell culture plate, with 105 cells per well. Once the cells have adhered to the wall (usually requiring 4 to 6 hours), replace the growth medium with the assay buffer. Place the cells in a 37°C incubator without CO2 for one hour, and then proceed with the machine detection. Add glucose (10 mM), Oligomycin (1 μM), and 2-DG (10 mM) in sequence.

### 2.8 Oxygen consumption rate assay

Hydration of probes: Add Seahorse XF calibration solution to the Utility Plate. Place the test plate back onto the Utility Plate and hydrate the probes overnight in a 37°C incubator without CO2. OCR detection solution: Take 100 ml of base medium and preheat it at 37°C. Add L-glutamine, glucose, and pyruvate to achieve final concentrations of 2 mM, 10 mM, and 1 mM, respectively. Adjust the pH to 7.4 and store the medium at 37°C. Cells processing and detection: U-251MG cells were seeded into a Seahorse XF cell culture plate, with 10^5^ cells per well. Once the cells have adhered to the wall (usually requiring 4 to 6 hours), replace the growth medium with the assay buffer. Place the cells in a 37°C incubator without CO2 for one hour, and then proceed with the machine detection. Add Oligomycin (1 μM), FCCP (1 μM), and antimycin A/rotenone (1 μM) in sequence.

### 2.9 Wound healing assay

U-251MG and LN229 cells were seeded into a 6-well plate (n = 3). When the cell density reached approximately 80%, a scratch was made using a 200 μl pipette tip. The cells were then washed with PBS and replaced with serum-free medium for 48 hours of culture. Photographs were taken at 0 and 48 hours, and the width of the scratch was measured and recorded.

### 2.10 EdU staining

U-251MG and LN229 cells were seeded into the dishes specifically designed for laser confocal microscopy, and stain the cells when the cell density reaches 80% (n = 3). The cells were sequentially processed through EdU labeling (Beyotime, C0075S, China), fixation, washing, permeabilization, and counterstaining with Hoechst 33342. Following these steps, laser confocal microscopy (Olympus, FV4000) was used to capture images and quantify the proportion of cells labeled with EdU.

### 2.11 Transwells assay

Dilute the Matrigel with serum-free medium at a ratio of 1:8. Then, take 60 μl of the diluted Matrigel and evenly apply it to the upper chamber surface of the membrane at the bottom of the Transwell insert. Place the Transwell insert in a 37°C incubator for 3 hours to allow the Matrigel to polymerize into a thin film. After incubation, aspirate the excess liquid in the upper chamber and add 100 μl of serum-free medium to each well. Let it stand in the incubator for 30 minutes to hydrate the basement membrane. Select U-251MG and LN229 cells during their logarithmic growth phase. Digest the cells, centrifuge them, and resuspend them in serum-free medium. Adjust the cell density to 1*10^5^ cells per milliliter. Place the Transwell insert into a 24-well plate, and add 200 μl of cell suspension into the insert. Add 650 μl of complete medium to each well of the 24-well plate. Subsequently, incubate the cells normally in an incubator for 48 hours (n = 3). Use a cotton swab to wipe off the cells on the upper layer of the Transwell insert, and fix the cells with pre-cooled methanol for 15 minutes. Then stain the cells with crystal violet at room temperature for 20 minutes, wash them twice with PBS, air-dry them at room temperature, take photographs under a microscope, and count the number of cells.

### 2.12 Western blotting and immunofuorescence

U-251MG and LN229 cells were inoculated into a 6-well plate and collect proteins when the cell density reaches 80%. Lyse the cells using RIPA lysis buffer and centrifuge to obtain the supernatant. Determine protein concentration using a BCA Protein Concentration Assay Kit (Beyotime, P0009, China). Subject the proteins to polyacrylamide gel electrophoresis, transfer the membrane, incubate with primary and secondary antibodies, and detect the expression level of the target protein using an ECL chemiluminescence kit. U-251MG and LN229 cells were seeded into the dishes specifically designed for laser confocal microscopy, and stain the cells when the cell density reaches 40%. Cells were undergoing fixation, permeabilization, blocking, incubation with primary and secondary antibodies, and counterstaining with Hoechst 33342, then imaged using a laser scanning confocal microscope. Western blotting (WB) and immunofuorescence were performed using antibodies against NFIA (CST, #69375, Ameica), LDHA (proteintech, 19987-1-AP, China), SMC4 (proteintech, 24758-1-AP, China), GAPDH (affinity, AF7021, China), Smad2/3 (affinity, AF6367, China), p-Smad2/3 (affinity, AF3367, China), E-Cadherin (affinity, BF0219, China), N-Cadherin (affinity, AF5239, China), SNAI1 (affinity, AF6032, China), ZEB1 (affinity, DF7414, China) as primary antibodies. Goat anti-rabbit IgG (H+L) (affinity, S0001, China) was used as secondary antibodies for Western blotting. Goat Anti-Rabbit IgG (H+L) CY3-conjugated (affinity, S0011, China) was used as secondary antibodies for immunofuorescence.

### 2.13 Dual luciferase reporter gene assay

We entrusted Shanghai GeneChem Co., Ltd. to synthesize the wild-type sequence of 2000 bp upstream of the transcription start site of the human SMC4 gene, as well as sequences with mutations in single or multiple potential binding sites of NFIA, and cloned them into GPL4-Basic respectively. The obtained plasmids were co-transfected into LN229 cells along with a Renilla luciferase construct. After 48 hours of transfection, the cells were collected and lysates were prepared. Luciferase activity was then measured using a Dual-Luciferase Reporter Assay Kit (YEASEN, 1142ES60, China).

### 2.14 ChIP assay

The experiment was conducted using the ChIP Assay Kit (Beyotime, P2078, China) with the following specific steps. Ln229 cells were normally cultured in a 10 cm dish, and when the cell density reached approximately 80%, formaldehyde with a final concentration of 1% was used for DNA and protein crosslinking, followed by glycine with a final concentration of 125 nM for decrosslinking. After washing the cells twice with PBS and transferring them to a 1.5 ml centrifuge tube, the cells were obtained by centrifugation at 4°C for 2 minutes (1000 g). The cells were resuspended in SDS Lysis Buffer (0.2 ml) and lysed on ice for 10 minutes. The lysate was then placed in an ice-water mixture and sonicated to obtain DNA fragments ranging in size from 200 to 1000 bp. The supernatant (about 0.2 ml) was obtained by centrifugation at 4°C for 5 minutes (12000 g) and mixed with 1.8 ml of ChIP Dilution Buffer. Twenty microliters (20 μL) of the mixture were taken as the input, while the remaining part was mixed with Protein A+G Agarose/Salmon Sperm DNA (70 μL) and gently mixed at 4°C for 30 minutes. The supernatant was then transferred to a 2 ml centrifuge tube after centrifugation at 4°C for 1 minute (1000 g). An appropriate amount of primary antibody was added, and the mixture was placed on a vertical rotating mixer at 4°C overnight. Protein A+G Agarose/Salmon Sperm DNA (60 μL per tube) was added and gently mixed at 4°C for 1 hour. The precipitate was collected by centrifugation at 4°C for 1 minute (1000 g) and washed sequentially with Low Salt Immune Complex Wash Buffer, High Salt Immune Complex Wash Buffer, LiCl Immune Complex Wash Buffer, and TE Buffer. The precipitate was dissolved in DEPC water and used as a template for qRT-PCR amplification to detect the target gene sequence.

### 2.15 Subcutaneous xenograft tumor model and tail vein metastasis model in mice

Six-week-old female BALB/c Nude were obtained from the Model Animal Research Institute of Nanjing University. Normally cultured LN229 cells were digested, centrifuged, resuspended in PBS, and centrifuged again. Afterward, the cells were resuspended in serum-free medium to a density of 5×10^6^ cells per milliliter. The cell suspension was then mixed with Matrigel in a 1:1 ratio, and 200 μl of this mixture was injected subcutaneously into the dorsum of each mouse to induce tumor formation. Measure the tumor volume starting from the 8th day of tumor bearing and every 3 days until the mice are euthanized. The formula for calculating tumor volume is as follows: Volume = Length * Width^2^/2. After sacrificing the mice, tumor tissues were collected for photography, weighed, and used to plot the tumor growth curve. A metastasis model was constructed by injecting LN229 cell suspension via the tail vein (100 μL per mouse, 1×10^6^ cells/mL). Four weeks after the injection, an *in-vivo* small animal imaging system (BIO-OI, LASER6000, China) was used to detect the signal intensity of metastatic foci. The Animal Research Protocol was approved by the Shanxi Provincial People’s Hospital’s Ethics Committee (2021-191).

### 2.16 Development of SMC4-related risk score

The CGGA_325 dataset was used as the training set to develop the SRRS, and the CGGA_693 and CGGA_301 datasets were served as the validation sets. Firstly, patients in the CGGA_325 cohort were divided into two groups based on the median value of SMC4, and 7,647 differentially expressed genes (logFC ≥ 2; adj.P.Val < 0.05) were obtained. Secondly, 307 genes co-expressed with SMC4 (corFilter ≥ 0.7) were identified. By combining the two sets, 281 genes were subjected to univariate regression analysis, which revealed that all of them were significantly associated with the patients’ overall survival (p < 0.01). Then, the 281 genes were input into the least absolute contraction and selection operator (LASSO) regression model, which generated 15 key genes and their corresponding coefficients. A new score for each patient was calculated by the formula as follows: score = ∑i Coefficient(Gene i) ∗Expression(Gene i). SRRS = 0.1443 × ACTL6A - 0.0038 × BICD1 + 0.0091 × CEP135 + 0.0889 × CKS2 + 0.3367 × CNIH4 + 0.0893 × DDOST + 0.0257 × EML4 + 0.0548 × FAM136A - 0.0829 × GMPS + 0.0632 × KLHDC8A + 0.029 × PLAT + 0.0736 × RAD54B - 0.0754 × RRM1 + 0.0675 × SLC30A7 + 0.7343 × TXLNA.

### 2.17 Statistical analysis

In this study, R 4.1.0 was used for statistical analysis and image rendering. Wilcox test was used to identify the difference between two groups. The log-rank test and Kaplan-Meier method were used for survival analysis between different groups. Less than 0.05 p-value was considered statistically significant. All *in vitro* assays (CCK-8, EdU, Transwell, glycolysis assays) were performed with 3 technical replicates and repeated in 3 independent biological experiments.

## 3 Results

### 3.1 Integrated multi-omics analysis identifies SMC4 as a key oncogene and prognostic indicator in glioma

**Figure 1:**
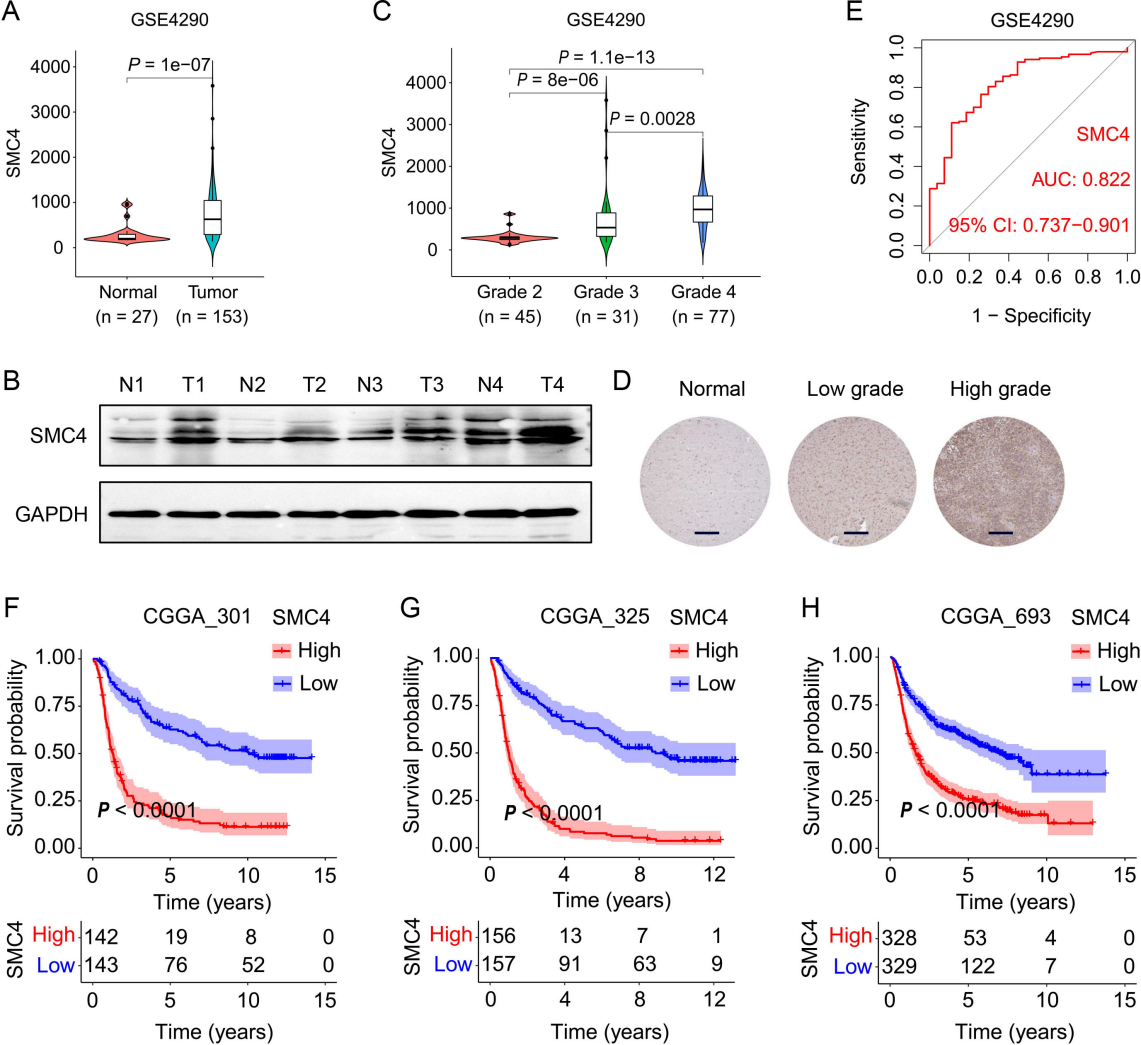
SMC4 overexpression is associated with glioma malignancy and predicts poor clinical outcome. **(A)** SMC4 mRNA expression in normal brain tissues versus glioma tissues from the GSE4290 dataset. **(B)** WB analysis of SMC4 protein levels in human glioma tissues and adjacent non-tumorous tissues. **(C)** Grade-dependent SMC4 mRNA upregulation in the GSE4290 cohort. **(D)** IHC staining of SMC4 in normal cerebral cortex, LGG, and high-grade gliomas (HGG) from the ProteinAtlas database. **(E)** Receiver operating characteristic (ROC) curve analysis of SMC4 expression for glioma diagnosis in the GSE4290 dataset. **(F-H)** Kaplan-Meier survival curves for patients with gliomas stratified by SMC4 expression in three independent CGGA cohorts (CGGA_301, CGGA_325, CGGA_693; *P* < 0.0001).

SMC4, a core subunit of the condensin complex, orchestrates chromosome dynamics, DNA damage repair, and mitotic accuracy, thereby influencing tumor initiation and progression (21). Emerging evidence underscores its oncogenic role in multiple cancer types. Leveraging multi-dataset analyses (GSE4290, GSE29796, GSE50161), we observed significantly elevated SMC4 expression in glioma tissues compared to normal brain parenchyma (*P* < 0.0001; **Figure 1A**, **Supplementary Figures 1A, B**). Western blot (WB) validation in primary human glioma specimens confirmed upregulated SMC4 protein levels (**Figure 1B**). Notably, SMC4 expression exhibited a grade-dependent increase across independent cohorts (GSE4290, CGGA_301, CGGA_325, CGGA_693; **Figure 1C**, **Supplementary Figures 1C–E**), with immunohistochemical (IHC) analysis of ProteinAtlas-derived normal cortex and glioma tissues mirroring this trend at the protein level (**Figure 1D**), consistent with mRNA expression patterns. These multi-omics findings collectively implicate SMC4 dysregulation in gliomagenesis. Diagnostic utility analyses revealed area under the ROC curve (AUC) values > 0.82 across GSE4290, GSE29796, and GSE50161 datasets (**Figure 1E**, **Supplementary Figures 1F, G**), highlighting robust discriminatory power for SMC4. Survival analyses in three independent CGGA cohorts (CGGA_301, CGGA_325, CGGA_693) further demonstrated that high SMC4 expression correlated with inferior patient prognosis (**Figures 1F–H**). Taken together, our integrative multi-dataset approach establishes SMC4 overexpression as a molecular signature of glioma progression and a clinically relevant prognostic biomarker.

### 3.2 SMC4 promotes glioma progression by accelerating cell cycle transition and enhancing proliferation *in vitro* and *in vivo*

**Figure 2:**
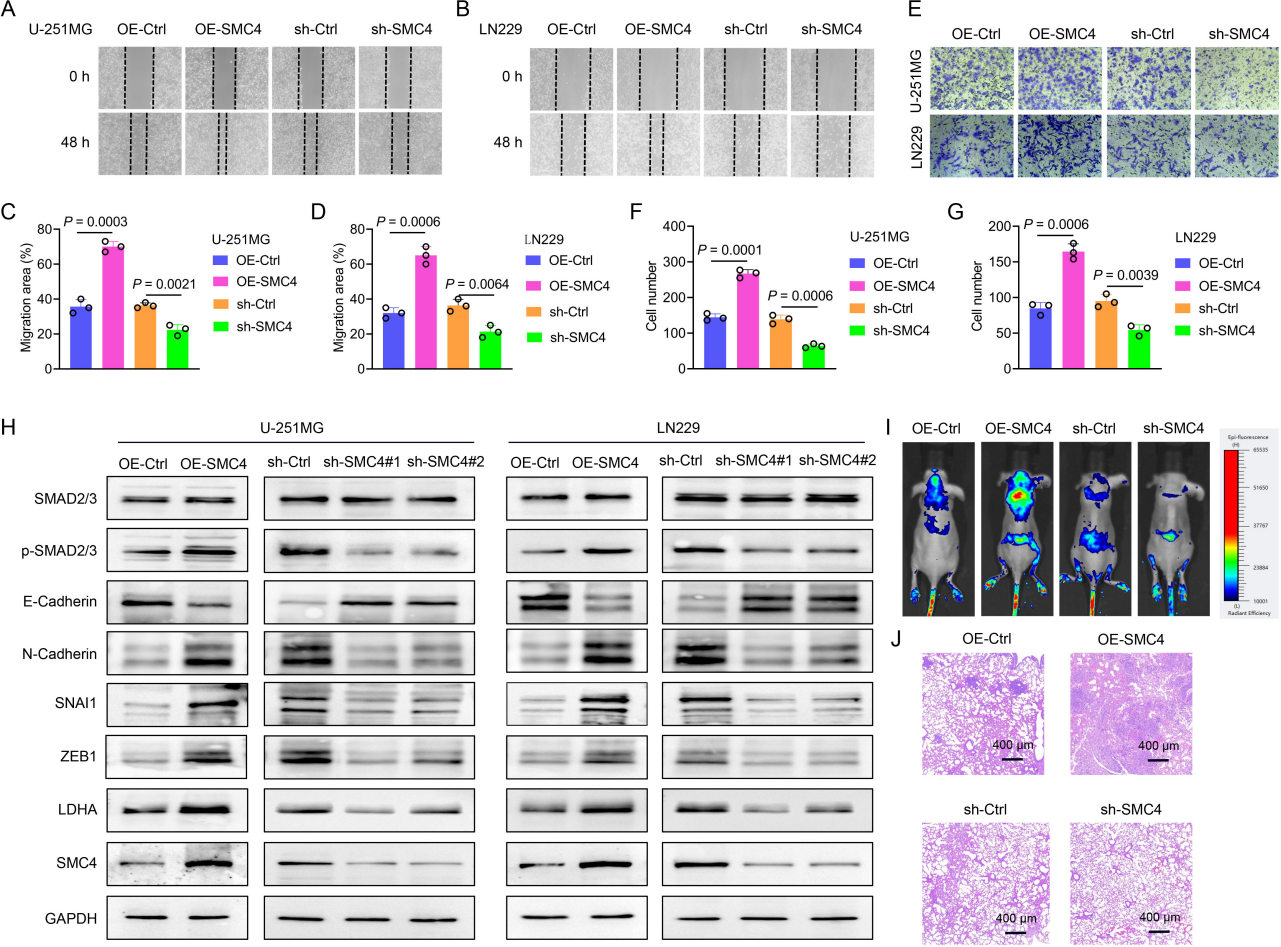
SMC4 promotes the metastasis of glioma both *in vitro* and *in vivo.* **(A-D)** The wound-healing assay was performed to evaluate the effect of SMC4 overexpression/knockdown on the migratory capacity of U-251MG and LN229 cells. Panels A and B show representative images of the wound-healing assay. Statistical analysis revealed that SMC4 overexpression enhanced the migratory capacity of U-251MG and LN229 cells by 96% and 103%, respectively, while SMC4 knockdown reduced their migratory capacity by 39% and 41%, respectively. **(E-G)** The Transwell assay was conducted to examine the effects of SMC4 overexpression/knockdown on the invasive capabilities of U-251MG and LN229 cells. Figure E presents a representative image of the Transwell assay. Statistical results demonstrated that SMC4 overexpression increased the invasive abilities of U-251MG and LN229 cells by 85% and 94% respectively, whereas SMC4 knockdown decreased the invasive abilities of these two cell lines by 54% and 43% respectively. **(H)** The WB assay was used to detect the changes in the expression levels of the core members of the TGF-β/SMAD signaling pathway in U-251MG and LN229 cells after SMC4 overexpression/knockdown, aiming to confirm the activating effect of SMC4 on the TGF-β/SMAD signaling pathway. **(I)** A tail vein metastasis model was established in BALB/c nude mice using LN229 cells with stable SMC4 overexpression/knockdown, and an *in vivo* animal imaging system was used to detect the colonization and growth of tumor cells in the lungs of mice. The images show the distribution of tumor cells in mice at the 6th week after modeling. **(J)** Six weeks after modeling, the mice were euthanized, and lung tissues were collected for HE staining to observe the size and number of metastatic foci in the lung tissues.

**Figure 3:**
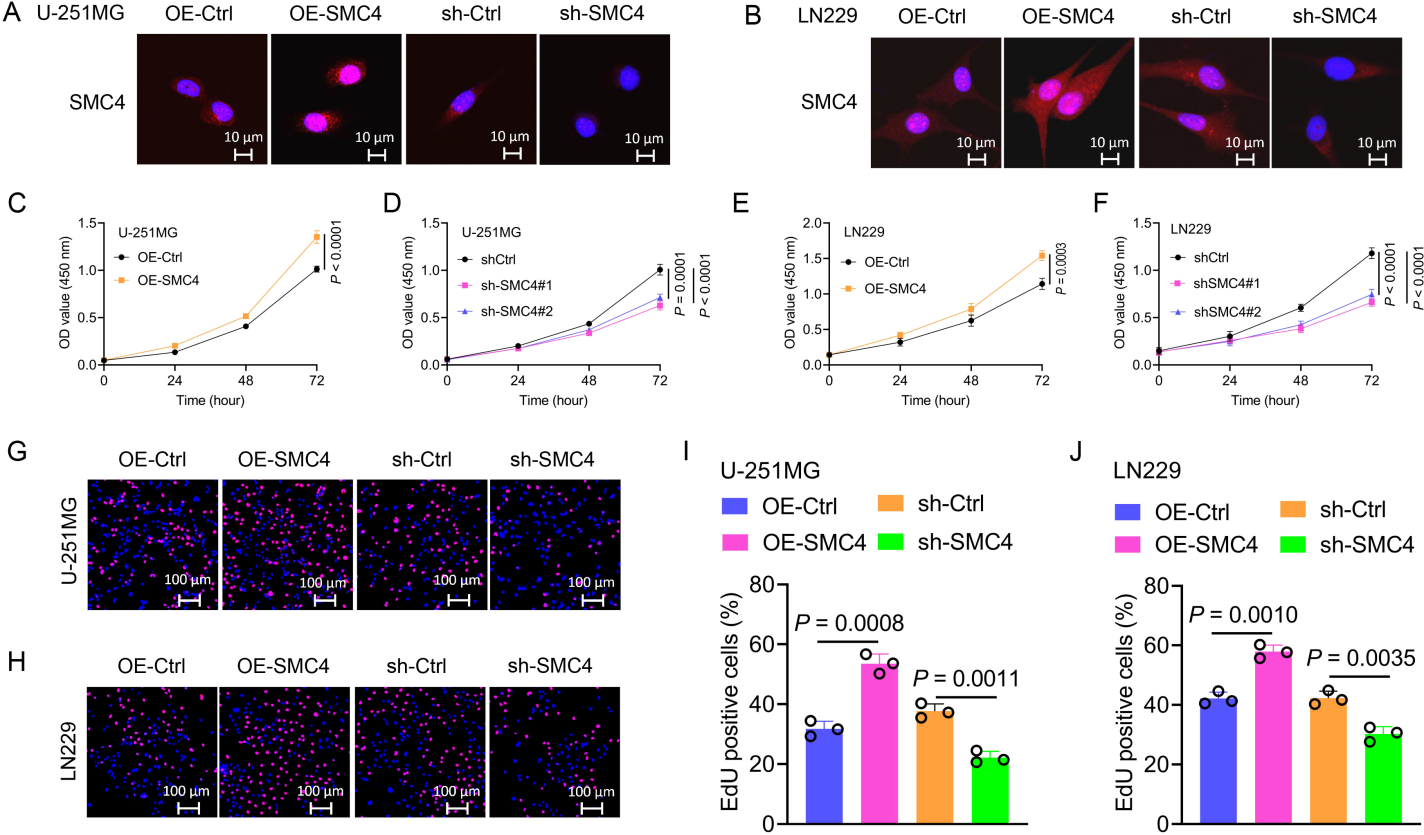
SMC4 promotes the proliferation of glioma cells *in vitro.* **(A, B)** Validation of SMC4 modulation in U-251MG and LN229 cells. Lentiviral transduction was used to generate stable SMC4-overexpressing (OE-SMC4) and knockdown (sh-SMC4) cell lines. IF imaging visualized SMC4 expression changes (sh-SMC4#1 was used for the cell IF assay). **(C-F)** CCK-8 proliferation assays. SMC4 overexpression significantly increased viability in U-251MG **(C, D)** and LN229 **(E, F)** cells, while SMC4 knockdown (two distinct shRNA sequences) exerted opposite effects (n = 4 technical replicates per group; *P* < 0.0001). **(G-J)** EdU incorporation assays. SMC4 overexpression enhanced the positive cell rate of EdU in U-251MG (from 31.8% to 53.5%, *P* = 0.0008) and LN229 (from 42.1% to 57.9%, *P* = 0.0010), whereas knockdown reduced positive rates (U-251MG: 22.2% vs. 37.7% control, *P* = 0.0011; LN229: 30.2% vs. 42.3% control, *P* = 0.0035; n = 3 fields of view per sample; representative images in **(G, H)** quantitative data in **(I, J)** sh-SMC4#1 was used for the EdU assay).

**Figure 4:**
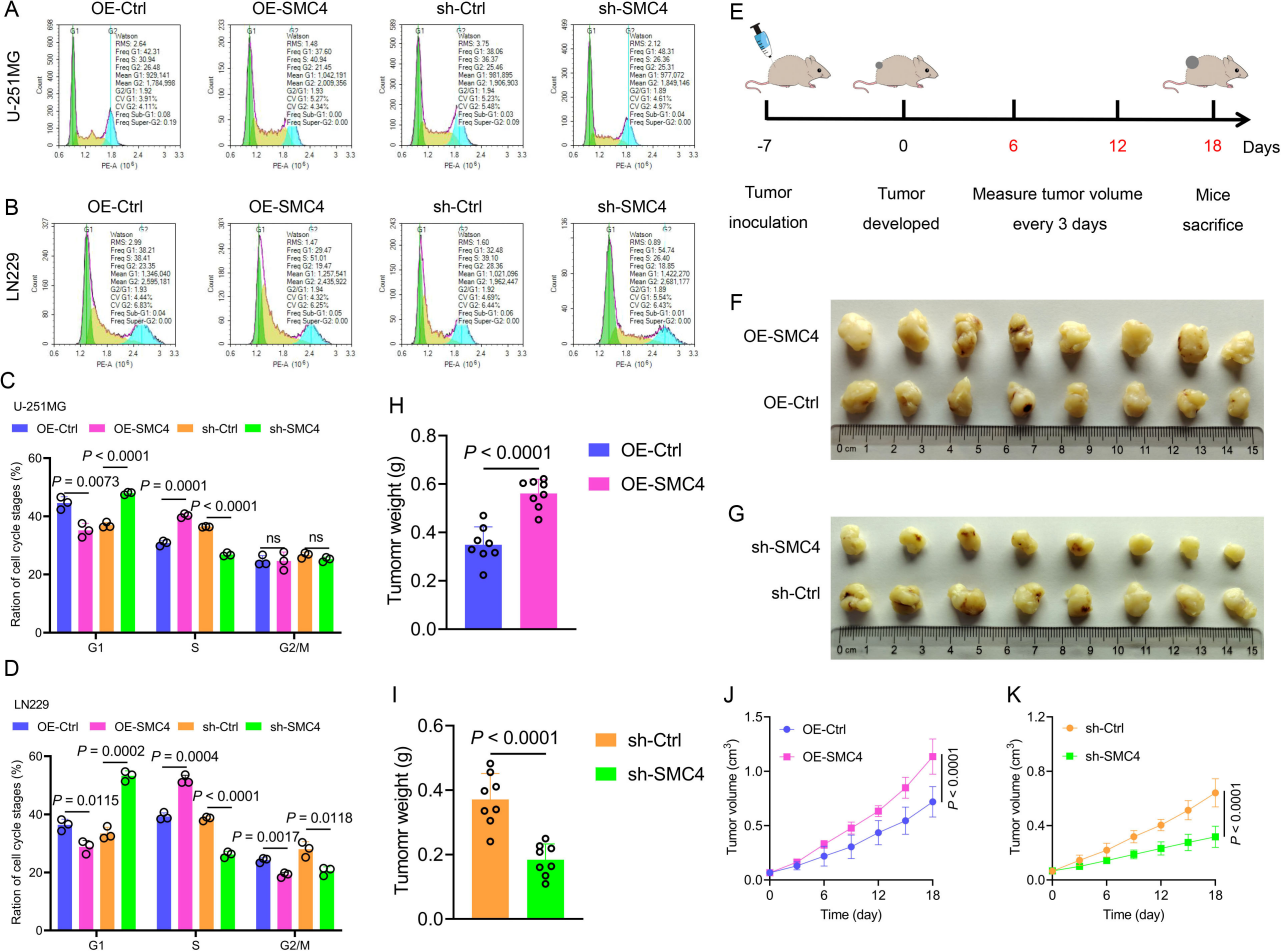
SMC4 promotes glioma cell cycle progression and tumor growth *in vivo.* **(A-D)** Flow cytometry analysis of cell cycle distribution in U-251MG and LN229 cells. SMC4 overexpression (OE-SMC4) reduced G1-phase proportion and increased S-phase accumulation, while SMC4 knockdown (sh-SMC4) exerted opposite effects. Representative flow plots **(A, B)** and quantitative analysis **(C, D)** are shown (n = 3 independent experiments). **(E)** Schematic of LN229-derived xenograft model establishment in BALB/c nude mice (subcutaneous implantation; n = 8 mice per group). **(F-K)** *In vivo* tumor growth analysis. Representative images of excised tumors from OE-Ctrl, OE-SMC4, sh-Ctrl, and sh-SMC4 groups **(F, G)**. Tumor weight quantification **(H, I)** and longitudinal growth curves **(J, K)** demonstrated significantly enhanced tumorigenicity in OE-SMC4 versus OE-Ctrl, and reduced growth in sh-SMC4 versus sh-Ctrl.

To systematically validate the oncogenic role of SMC4 in glioma, we generated stable SMC4-overexpressing and SMC4-knockdown U-251MG/LN229 cell lines via lentiviral transduction. WB analysis first confirmed successful modulation of SMC4 protein levels (**Figures 2H**), which was further visualized by immunofluorescence (IF) imaging (**Figures 3A, B**). To assess the functional impact, we performed CCK-8 assays and observed that SMC4 overexpression significantly enhanced proliferation in both cell lines, whereas SMC4 knockdown (using two distinct targeting sequences) produced opposing effects (**Figures 3C–F**). EdU incorporation assays corroborated these findings: SMC4 overexpression increased the proportion of proliferating cells from 31.8% to 53.5% in U-251MG (*P* = 0.0008) and from 42.1% to 57.9% in LN229 cells (*P* = 0.0010), while SMC4 silencing reduced positive rates to 22.2% (vs. 37.7% control) and 30.2% (vs. 42.3% control), respectively (**Figures 3G–J**; **Supplementary Figure 2**). Mechanistic exploration via cell cycle analysis revealed that SMC4-overexpressing cells exhibited reduced G1-phase arrest (U-251MG: 35.2% vs. 44.6% control; LN229: 28.8% vs. 36.4% control) and enhanced S-phase accumulation (U-251MG: 40.2% vs. 30.7% control; LN229: 51.9% vs. 39.3% control), effects that were reversed by SMC4 knockdown (**Figures 4A–D**; **Supplementary Figure 3**). To translate these *in vitro* findings to *in vivo* settings, we established LN229-derived xenograft models in nude mice. Tumor growth was monitored longitudinally, and post-euthanasia analyses included photographic documentation and gravimetric assessment of excised tumors (**Figure 4E**). By day 25 post-implantation, SMC4-overexpressing tumors (OE-SMC4) displayed significantly larger volumes (1135 ± 151 mm³ vs. 719 ± 130 mm³ in OE-Ctrl, *P* < 0.0001) and weights (0.561 ± 0.055 g vs. 0.349 ± 0.069 g, *P* < 0.0001; **Figures 4F, H, J**). Conversely, SMC4-knockdown tumors (sh-SMC4) showed marked reductions in both parameters (318 ± 72 mm³ vs. 641 ± 96 mm³ in sh-Ctrl, *P* < 0.0001; 0.184 ± 0.046 g vs. 0.371 ± 0.075 g, *P* < 0.0001; **Figures 4G, I, K**). Collectively, these *in vitro* and *in vivo* data demonstrate that SMC4 promotes glioma cell proliferation and drives cell cycle progression, establishing its critical role in glioma tumorigenesis.

### 3.3 SMC4 facilitates glioma metastasis via enhancement of TGF-β/SMAD signaling

To further validate the oncogenic function of SMC4 in glioma, we explored its impact on the migratory and invasive capabilities of glioma cells. In the wound-healing assay, overexpression of SMC4 augmented the migratory capacity of U-251MG and LN229 cells by 96% and 103%, respectively. Conversely, knockdown of SMC4 led to a 39% and 41% decline in the migratory ability of these cells, respectively (**Figures 2A–D**; **Supplementary Figure 4**). The Transwell assay corroborated these findings, demonstrating that SMC4 overexpression elevated the invasive potential of U-251MG and LN229 cells by 85% and 94%, respectively. In contrast, SMC4 knockdown resulted in a 54% and 43% reduction in their invasive capacity, respectively (**Figures 2E–G**; **Supplementary Figure 5**). The WB analyses revealed that overexpression of SMC4 in U-251MG and LN229 cells significantly upregulated the expression levels of p-Smad2/3, SNAI1, ZEB1, and N-cadherin. In contrast, SMC4 knockdown elicited the opposite effect, with a notable downregulation of these proteins. These results imply that SMC4 may drive glioma metastasis through the aberrant activation of the TGF-β/SMAD signaling pathway (**Figure 2H**). To further substantiate the pro-tumorigenic effect of SMC4 in an *in vivo* setting, we established a tail-vein metastasis model using LN229 cells. Data from mouse *in vivo* imaging indicated that SMC4 overexpression markedly enhanced the distant metastatic potential of LN229 cells in mice. In contrast, SMC4 knockdown significantly attenuated the distant metastatic ability of these cells in mice (**Figure 2I**). Accidentally, the HE staining results showed that the number of lung metastasis foci in the SMC4 overexpression group mice was significantly higher than that in the control group, while the number in the SMC4 knockdown group mice was significantly lower than that in the control group (**Figure 2G**). Collectively, these *in vitro* and *in vivo* findings establish SMC4 as a critical facilitator of glioma metastasis by potently activating the TGF-β/SMAD signaling axis, highlighting its therapeutic potential as a target for inhibiting metastatic progression in glioma.

### 3.4 SMC4 enhances glycolysis in glioma cells by upregulating LDHA

**Figure 5:**
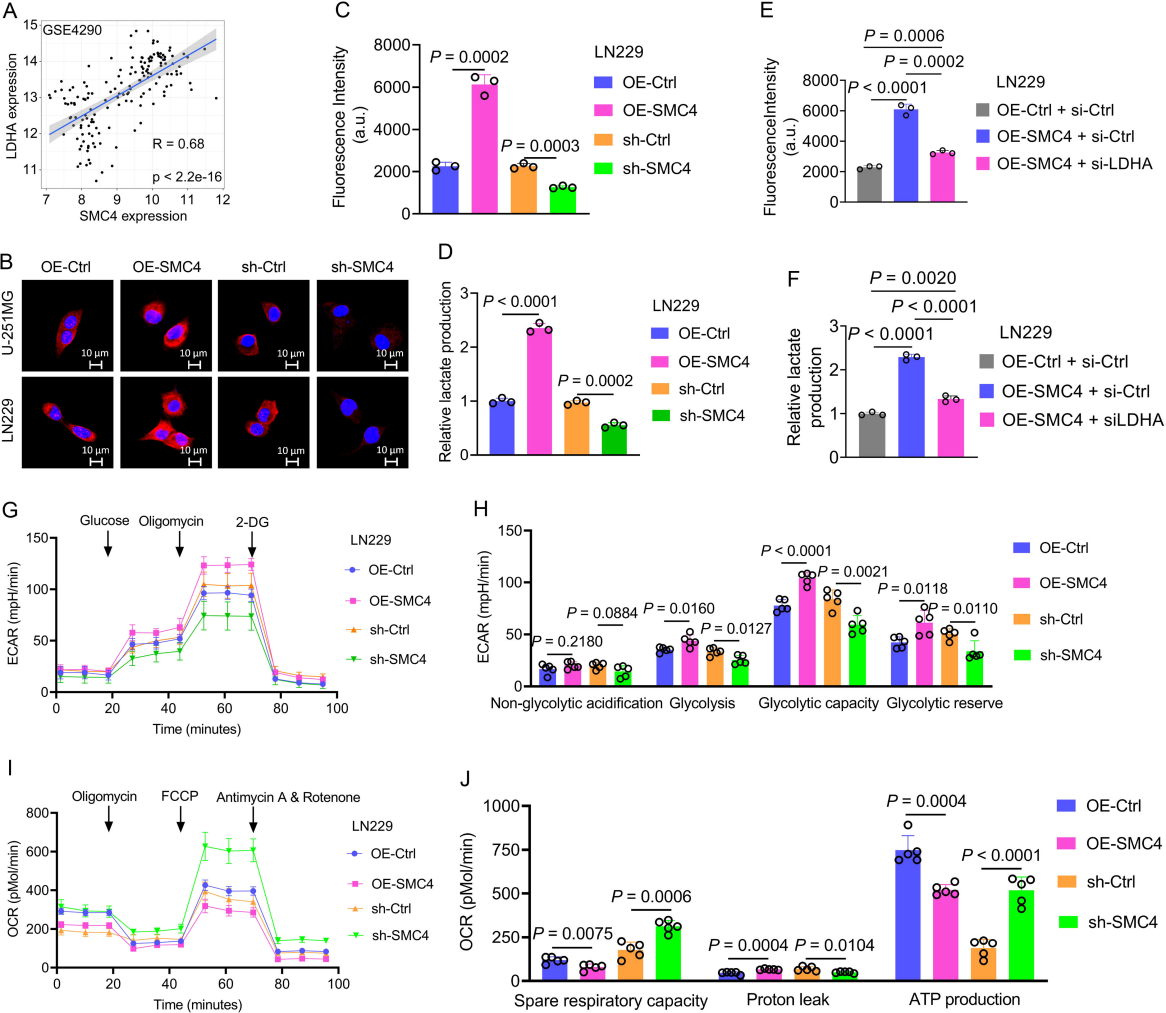
SMC4 promotes glycolysis in glioma cells by upregulating LDHA. **(A)** In the GSE4290 dataset, the expression levels of SMC4 and LDHA were significantly positively correlated. **(B)** The IF assay was used to detect the expression levels of LDHA in U-251MG and LN229 cells after SMC4 overexpression and SMC4 knockdown. **(C, D)** Results of glucose uptake and lactate production measurements in LN229 cells after SMC4 overexpression and SMC4 knockdown. **(E, F)** Rescue experiments showed that LDHA knockdown could reverse the increased glucose uptake and lactic acid production in U-251MG and LN229 cells caused by SMC4 overexpression. **(G-J)** The ECAR **(G)** and OCR **(I)** in LN229 cells after SMC4 overexpression and SMC4 knockdown were detected using a Seahorse cellular energy metabolism analyzer. Statistical results of ECAR **(H)** and OCR **(J)**.

To elucidate the molecular mechanism underlying SMC4-mediated malignant transformation in glioma cells, we first performed bioinformatics analysis across three independent glioma datasets. Correlation analysis revealed a significant positive association between SMC4 and LDHA expression in GSE4290 (R = 0.68; *P* < 2.2e-16), CGGA_325 (R = 0.56; *P* < 2.2e-16), and CGGA_693 (R = 0.59; *P* < 2.2e-16) (**Figure 5A**; **Supplementary Figure 6**). To validate this transcriptional correlation, the WB and IF assays were conducted in U-251MG and LN229 cells. Forced expression of SMC4 significantly upregulated LDHA protein levels, whereas SMC4 knockdown resulted in marked LDHA downregulation (**Figures 2H**, **5B**), suggesting SMC4 may regulate tumor energy metabolism. Functional metabolic assays were then performed to characterize SMC4’s role in glycolysis. SMC4 overexpression increased glucose uptake in LN229 cells by 1.72-fold, while SMC4 depletion reduced glucose uptake by 44.1% (**Figure 5C**). Concomitantly, SMC4 overexpression enhanced lactic acid secretion by 1.35-fold, whereas SMC4 knockdown decreased lactic acid production by 43% (**Figure 5D**). Genetic rescue experiments revealed that LDHA knockdown significantly reversed the SMC4-induced increases in glucose uptake (75% inhibition) and lactic acid production (74% inhibition) (**Figures 5E–F**), confirming LDHA as a critical downstream effector of SMC4 in glycolytic regulation. Extracellular acidification rate (ECAR) and oxygen consumption rate (OCR) analyses were used to profile global metabolic shifts. SMC4 overexpression significantly elevated ECAR, reflecting enhanced glycolytic flux, while SMC4 knockdown had the opposite effect (**Figure 5G**). Quantitative analysis showed that SMC4 overexpression increased glycolysis, glycolytic capacity, and glycolytic reserve, whereas SMC4 depletion attenuated these parameters (**Figure 5H**). In contrast, OCR assays demonstrated that SMC4 overexpression reduced basal and maximal oxygen consumption, while SMC4 knockdown increased mitochondrial respiration (**Figure 5I**). Notably, SMC4 overexpression was associated with increased spare respiratory capacity and proton leak, coupled with decreased ATP productio-phenotypes indicative of mitochondrial uncoupling and metabolic reprogramming toward glycolysis (**Figure 5J**). Collectively, these findings demonstrate that SMC4 promotes glycolysis in glioma cells by upregulating LDHA, thereby providing metabolic energy support for malignant proliferation.

### 3.5 NFIA promotes SMC4 expression through transcriptional activation

**Figure 6:**
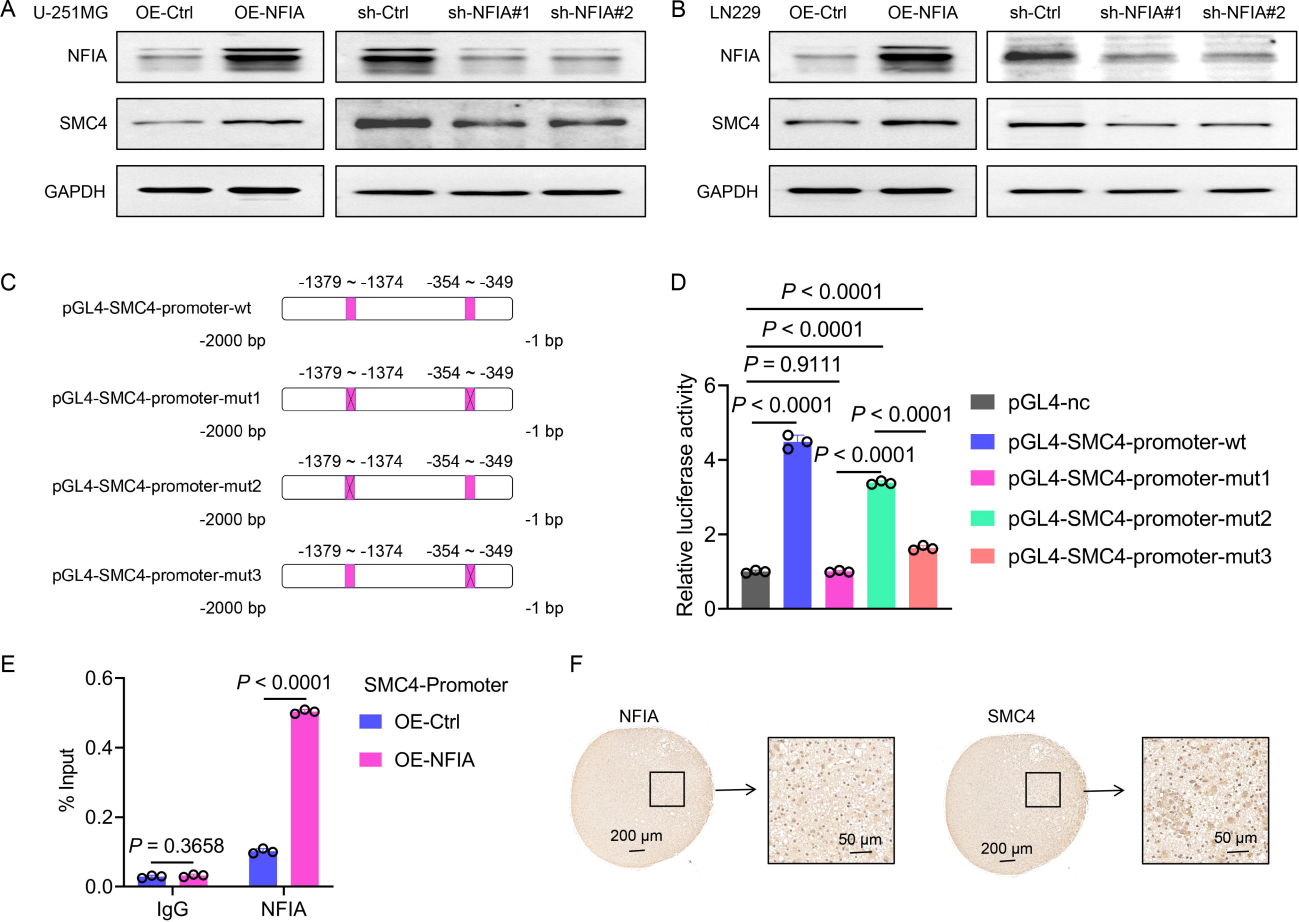
NFIA promotes SMC4 transcription through direct binding to its promoter region in glioma cells. **(A, B)** The WB assay was used to detect the effects of NFIA overexpression and NFIA knockdown in U-251MG and LN229 cells, as well as the expression level of SMC4. **(C)** Schematic diagram of wild-type (wt) and mutant (mut, with mutated NFIA binding site) sequences of the SMC4 promoter. **(D)** The wild-type and mutant sequences of the SMC4 promoter were cloned into the pGL4 vector, and co-transfected with the pRL-TK vector (Renilla luciferase internal reference) into LN229 cells. Compared with the empty vector control group, overexpression of NFIA significantly enhanced the luciferase activity in the wt, mut2, and mut3 groups (*P* < 0.0001), while there was no significant change in the mut1 group (*P* = 0.9111). **(E)** The ChIP assay validates direct binding of NFIA to the SMC4 promoter region. ChIP-qPCR analysis was performed in LN229 cells with NFIA overexpression or control vector. Enriched DNA fragments spanning the predicted NFIA binding sites (-354 to -349 bp) in the SMC4 promoter were quantified and normalized to input DNA. **(F)** The IHC staining results of human gliomas showed that the expression of NFIA and SMC4 exhibited the same trend.

To investigate the regulatory relationship between NFIA and SMC4, we first analyzed their expression correlation using the GEPIA (Gene Expression Profiling Interactive Analysis) database. In both LGG (n = 532) and GBM (n = 169) datasets, NFIA and SMC4 exhibited a significant positive correlation (R = 0.34, *P* = 8.9e-16 for LGG; R = 0.35, *P* = 5.6e-06 for GBM; **Supplementary Figure 7**), suggesting that NFIA may transcriptionally regulate SMC4. To further investigate this relationship, we employed lentiviruses to generate U-251MG and LN229 cell lines with stable NFIA overexpression and stable NFIA knockdown. The WB assays were then carried out. The results demonstrated that SMC4 expression levels were significantly upregulated in both cell lines following NFIA overexpression. Conversely, when NFIA was knocked down using two distinct target sites, SMC4 expression levels were markedly decreased in both cell lines (**Figures 6A, B**). Using the JASPAR database, we identified two potential NFIA binding motifs in the SMC4 promoter region: a distal site (-1379 to -1374 bp) and a proximal site (-354 to -349 bp; **Figure 6C**). Dual-luciferase reporter assays demonstrated that co-transfection of NFIA expression vectors significantly enhanced luciferase activity driven by the wild-type SMC4 promoter (*P* < 0.0001). Strikingly, simultaneous mutation of both sites abrogated this activation, resulting in a 78% reduction in luciferase activity compared to wild-type controls (*P* < 0.0001). Individual mutation of the distal and proximal sites reduced luciferase activity by 24% (*P* < 0.0001) and 63% (*P* < 0.0001), respectively (**Figure 6D**), confirming that both sites contribute to NFIA-mediated transcriptional activation, with the proximal site playing a dominant role. To directly demonstrate physical interaction between NFIA and the SMC4 promoter, we performed chromatin ChIP assays in LN229 cells. Quantitative PCR analysis showed that NFIA overexpression increased binding to the SMC4 promoter region (-354 to -349 bp) by 2.52-fold compared to control cells (*P* < 0.0001; **Figure 6E**), providing *in vitro* evidence of direct transcriptional regulation. Finally, we collected human glioma samples and performed IHC staining on the tissue sections. The IHC results corroborated the positive correlation between NFIA and SMC4 expression levels, showing a consistent trend in the human glioma specimens (**Figure 6F**). Collectively, through integrative analyses of genomic databases, functional cell-based assays, and clinical tissue validation, we establish that NFIA promotes SMC4 expression by directly binding to its promoter region and enhancing transcriptional activity. This NFIA/SMC4 regulatory axis represents a potential therapeutic target for glioma and underscores the importance of transcriptional networks in gliomagenesis.

### 3.6 The SMC4-related risk score effectively predicts patient prognosis and guides clinical medication

**Figure 7:**
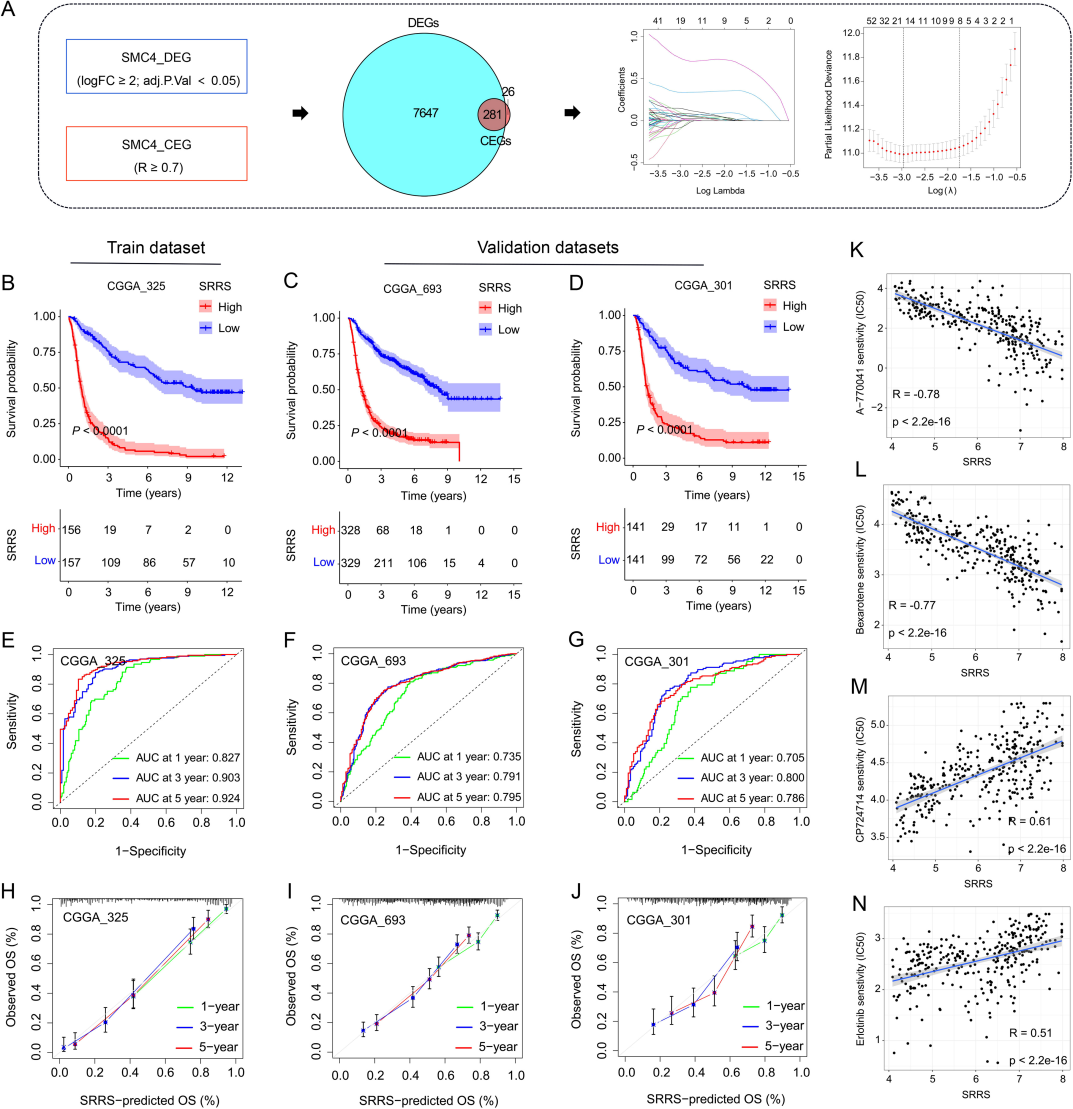
Development and validation of SMC4-related risk score. **(A)** Flowchart for the development of SRRS. **(B-D)** The KM curves were used to evaluate patient prognosis, and the results showed that patients with high SRRS had poor prognosis in both the training and validation sets. **(E-G)** The ROC curves and AUC value of SRRS for 1-, 3-, and 5-year in the training and validation cohorts. **(H-J)** Calibration curves were used to evaluate the consistency between the survival outcomes predicted by SRRS and the observed survival outcomes. **(K-N)** Correlation analysis of SRRS with glioma patients’ sensitivity to A-770041, bexarotene, CP724714, and erlotinib.

**Figure 8:**
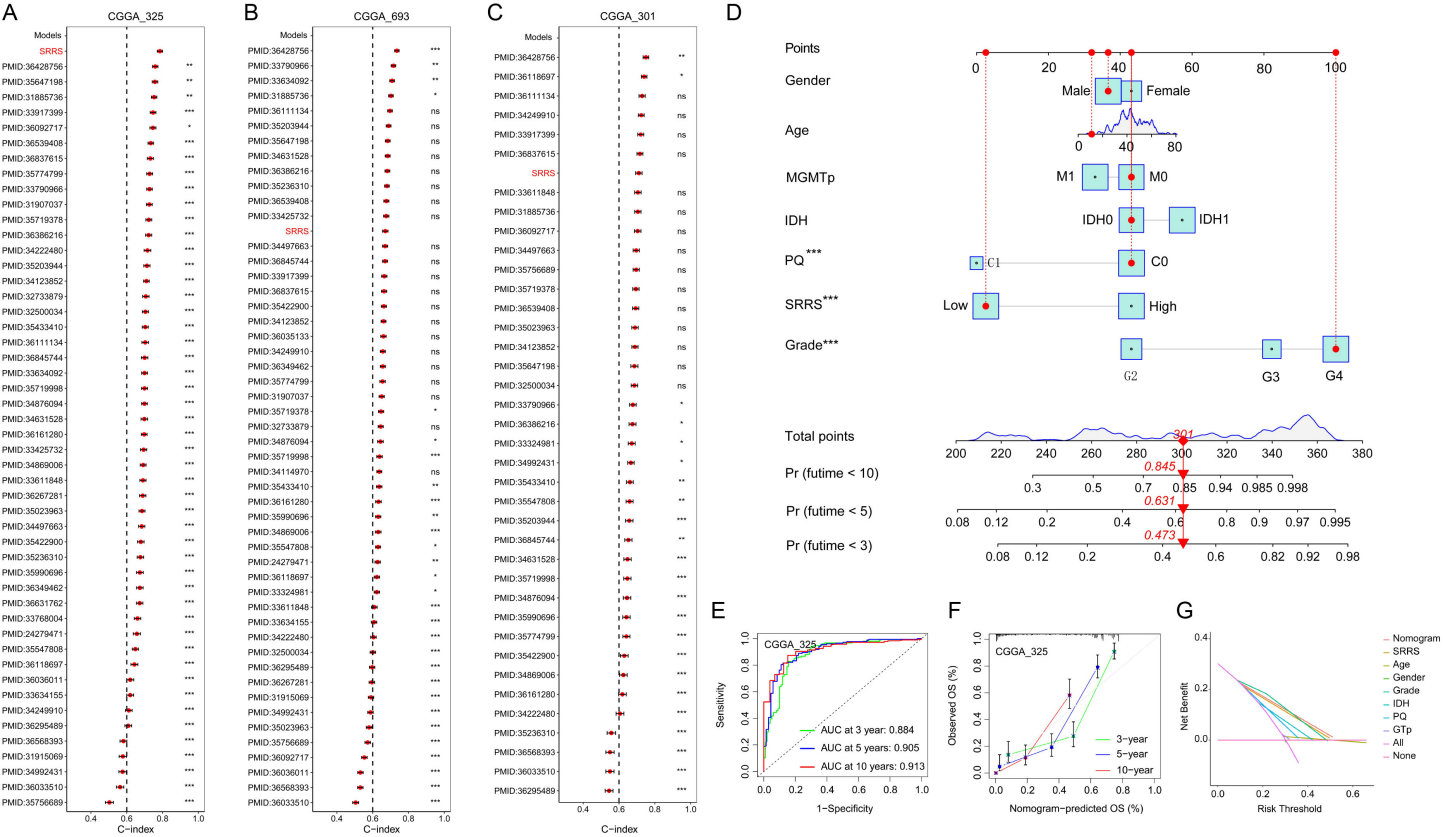
Construction and evaluation of a nomogram based on SRRS and clinical indicators of glioma. **(A-C)** The C-index analyses of SRRS and 49 published mRNA signatures in CGGA_325 **(A)**, CGGA_69 **(B)**, and CGGA_301 **(C)** datasets. Statistic tests: two-sided z-score test. Data are presented as mean ± 95% confidence interval. **(D)** A nomogram was developed using SRRS along with age, gender, tumor grade, MGMTp, IDH, and PQ to quantify patient prognosis. **(E)** Time-dependent ROC curves were used to evaluate the reliability of the nomogram in predicting prognosis for glioma patients. **(F)** Calibration curves were used to evaluate the consistency between the survival outcomes predicted by nomogram and the observed survival outcomes. **(G)** Decision curve analysis (DCA) was used to evaluate the practical value of the predictive nomogram in clinical practice.

Both prior studies and our current investigations confirm that SMC4 orchestrates critical biological pathways driving glioma tumorigenesis and progression. Leveraging this mechanistic understanding, we developed the SRRS to stratify glioma patient prognosis (**Figure 7A**). The CGGA_325 dataset served as the training cohort for SRRS development, while CGGA_693 and CGGA_301 were employed as independent validation cohorts to assess model generalizability. Kaplan-Meier (KM) survival analysis revealed that patients with high SRRS scores exhibited significantly poorer overall survival (OS) in both the training cohort (**Figure 7B**) and validation cohorts (**Figures 7C, D**). Time-dependent receiver operating characteristic (ROC) curve analysis further validated the prognostic utility of SRRS, with area under the curve (AUC) values > 0.7 for 1-, 3-, and 5-year OS across all cohorts (**Figures 7E–G**). Calibration curve analyses demonstrated strong agreement between predicted and observed survival outcomes (**Figures 7H–J**), underscoring SRRS as a clinically reliable prognostic biomarker. Pharmacogenomic analysis revealed that SRRS was strongly negatively correlated with glioma patients’ sensitivity to A-770041 and bexarotene, while strongly positively correlated with sensitivity to CP724714 and erlotinib (**Figures 7K–N**). These findings establish that SRRS effectively evaluates glioma patient prognosis and provides a theoretical foundation for guiding individualized cancer therapy. When benchmarked against 49 previously published glioma-related mRNA signatures, the SRRS demonstrated superior performance in the CGGA_325 training dataset, ranking fifth in CGGA_693, and third in CGGA_301 validation datasets (**Figures 8A–C**). These results highlight the SRRS’s comparable or superior prognostic utility across independent cohorts, solidifying its potential as a robust prognostic biomarker. To integrate SRRS with clinical practice, we developed a nomogram incorporating age, gender, tumor grade, MGMT promoter status (MGMTp), IDH mutation status, and 1p19q codeletion status (PQ) (**Figure 8D**). The results of univariate and multivariate regression analyses showed that SRRS also was an independent prognostic factor for glioma in both validation sets (**Supplementary Figure 8**). ROC analysis showed that the nomogram achieved AUC values > 0.88 for 3-, 5-, and 10-year OS predictions (**Figure 8E**), with calibration curves demonstrating strong consistency between predicted and observed outcomes (**Figure 8F**). The DCA curve further confirms the superiority of the nomogram over other clinical indicators in evaluating patient prognosis (**Figure 8G**). Collectively, these findings establish the SRRS and nomogram as powerful tools for prognostic stratification and personalized treatment guidance in glioma patients, highlighting their translational potential for clinical oncology.

## 4 Discussion

Malignant gliomas, including GBM, represent a heterogeneous group of aggressive brain tumors with dismal clinical outcomes. Despite advances in multimodal therapy, GBM patients face a median survival of 14.6 months and a 5-year survival rate of only 7.2% (2, 3). The high recurrence rate and intrinsic resistance to treatment underscore the urgent need for molecular insights into glioma pathogenesis. The high recurrence rate and intrinsic resistance to treatment underscore the urgent need for molecular insights into glioma pathogenesis. Here, we identify SMC4 as a multifunctional oncoprotein driving glioma progression through dual mechanisms: activation of TGF-β/Smad-mediated metastasis and LDHA-dependent metabolic reprogramming. Additionally, we develop a clinically relevant prognostic model, the SRRS, to stratify patient outcomes.

As a core component of the condensin complex, SMC4 governs chromosomal dynamics, DNA repair, and mitotic fidelity (7, 11). Our multi-omics analysis across multiple datasets (TCGA, CGGA, GEO, ProteinAtlas) revealed consistent upregulation of SMC4 in glioma tissues, with expression levels correlating with tumor grade and patient prognosis (**Figure 1**; **Supplementary Figure 1**). This is consistent with SMC4’s established roles in solid tumors, where it promotes oncogenesis via NF-κB and TGF-β/Smad signaling (14–16). In gliomas, SMC4 drives two critical oncogenic programs: (1) cell proliferation and cell cycle progression: SMC4 overexpression accelerates G1/S transition *in vitro* and enhances tumor growth in xenograft models (**Figures 3C–J**; **Figure 4**), consistent with its role in chromosomal condensation during mitosis (12); (2) metastatic spread via TGF-β/Smad Signaling: SMC4 promotes glioma cell migration and invasion by upregulating EMT markers (N-cadherin, SNAI1, ZEB1) and activating phosphorylated Smad2/3 (**Figure 2H**). *In vivo* tail-vein metastasis models confirm that SMC4 enhances distant dissemination (**Figures 2I, J**), aligning with its role in TGF-β pathway activation in other cancers (18).

Aerobic glycolysis (the Warburg effect) is a hallmark of glioma energy metabolism, and our data link SMC4 to this process through regulation of LDHA (**Figures 5A, B**; **Supplementary Figure 3**). SMC4 overexpression correlates with increased glucose uptake, lactate production, and ECAR, while reducing OCR, all of which are hallmarks of glycolytic dominance (**Figures 5C–G**). Mechanistically, SMC4 upregulates LDHA expression, a key enzyme in converting pyruvate to lactate, thereby regenerating NAD+ and sustaining glycolysis under hypoxic conditions (22). Genetic rescue experiments confirm that LDHA is essential for SMC4-mediated glycolytic enhancement, positioning LDHA as a critical downstream effector.

The transcription factor NFIA directly drives SMC4 expression. Bioinformatics analyses reveal a strong positive correlation between NFIA and SMC4 in glioma datasets, validated by co-expression in clinical specimens. Dual-luciferase reporter and ChIP assays demonstrate that NFIA binds two conserved motifs in the SMC4 promoter (distal -1379 bp and proximal -354 bp), with the proximal site exerting dominant regulatory effects (**Figure 6**). This finding establishes an NFIA/SMC4 regulatory axis, expanding our understanding of NFIA’s role in glioma beyond its known functions in cell cycle control (23) and chemotherapy resistance (24).

Leveraging SMC4’s prognostic value, we developed SRRS using LASSO regression in CGGA datasets. SRRS stratifies patients into high- and low-risk groups with distinct survival outcomes, validated across training (CGGA_325) and independent validation cohorts (CGGA_301, CGGA_693) (**Figure 7**). With AUC values >0.7 for 1-, 3-, and 5-year survival, SRRS outperforms 49 previously published glioma mRNA signatures. Integrating SRRS with clinical parameters (age, tumor grade, IDH status) into a nomogram further enhances prognostic accuracy (AUC > 0.88), providing a robust tool for personalized risk assessment (**Figure 8**). Pharmacogenomic analyses suggest SRRS may guide therapy selection, such as predicting sensitivity to erlotinib.

Our study establishes SMC4 as a therapeutic target with dual roles in glioma progression and metastasis. Targeting SMC4 or its upstream regulator NFIA could disrupt both chromosomal instability and metabolic reprogramming, while LDHA inhibition may synergize with glycolytic pathway blockers. As a core subunit of condensins, SMC4 plays a central role in the cell cycle (particularly during mitosis) (12) and DNA damage repair (11). Therefore, targeting SMC4 could theoretically synergize with anti-tumor drugs that induce DNA damage and disrupt the proliferation cycle of cancer cells, such as temozolomide and cisplatin. Small-molecule inhibitors of NFIA/SMC4 may require modification (e.g., lipophilic moieties) to cross the blood-brain barrier (BBB). Alternative strategies include local delivery via convection-enhanced delivery (CED) or nanoparticles conjugated to BBB-targeting ligands (e.g., transferrin receptors) (25). Emerging data on BBB-penetrant TGF-βinhibitors (e.g., galunisertib) provide a precedent for targeting downstream pathways of SMC4 (26). Given that NFIA (27) and SMC4 (28) are crucial for normal cell division, concerns about toxicity have been raised. However, such concerns may be alleviated through selective inhibition using tumor-specific promoters (e.g., GFAP for gliomas) or targeted delivery systems.

The SRRS model offers clinical utility for prognostic stratification and precision medicine, though validation in independent cohorts is warranted. Future studies will explore SMC4’s role in glioma stem cells and resistance to standard-of-care therapies like temozolomide. In summary, this work uncovers the multifaceted role of SMC4 in glioma biology and provides a translational framework for improving diagnosis and treatment. SMC4 and its regulatory network represent promising targets to address the unmet needs in this devastating disease.

At last, the limitation of this work should be pointed out that (1) Limitations of the samples: The distribution of patients with gliomas of different grades is uneven in both the training set and the validation set. In subsequent studies, separate validation using patients with gliomas at different stages should be conducted. (2) Glioma stem cells (GSCs): We did not evaluate the role of SMC4 in GSCs, which is crucial for recurrence. Future research should explore the role of SMC4 in GSC self-renewal. (3) Depth of mechanism: The exact molecular link between SMC4 and LDHA (such as transcriptional regulation and post-translational regulation) requires further investigation.

## Data Availability

The original contributions presented in the study are included in the article/**Supplementary Material**. Further inquiries can be directed to the corresponding authors.
