# Supplementary material for: NFIA-dependent upregulation of SMC4 promotes metastasis and metabolic reprogramming in glioma
Source: Front Oncol. 2025 Aug 25;15:1624370. doi: 10.3389/fonc.2025.1624370 (PMC12417569; doi:10.3389/fonc.2025.1624370)

Figure 1B

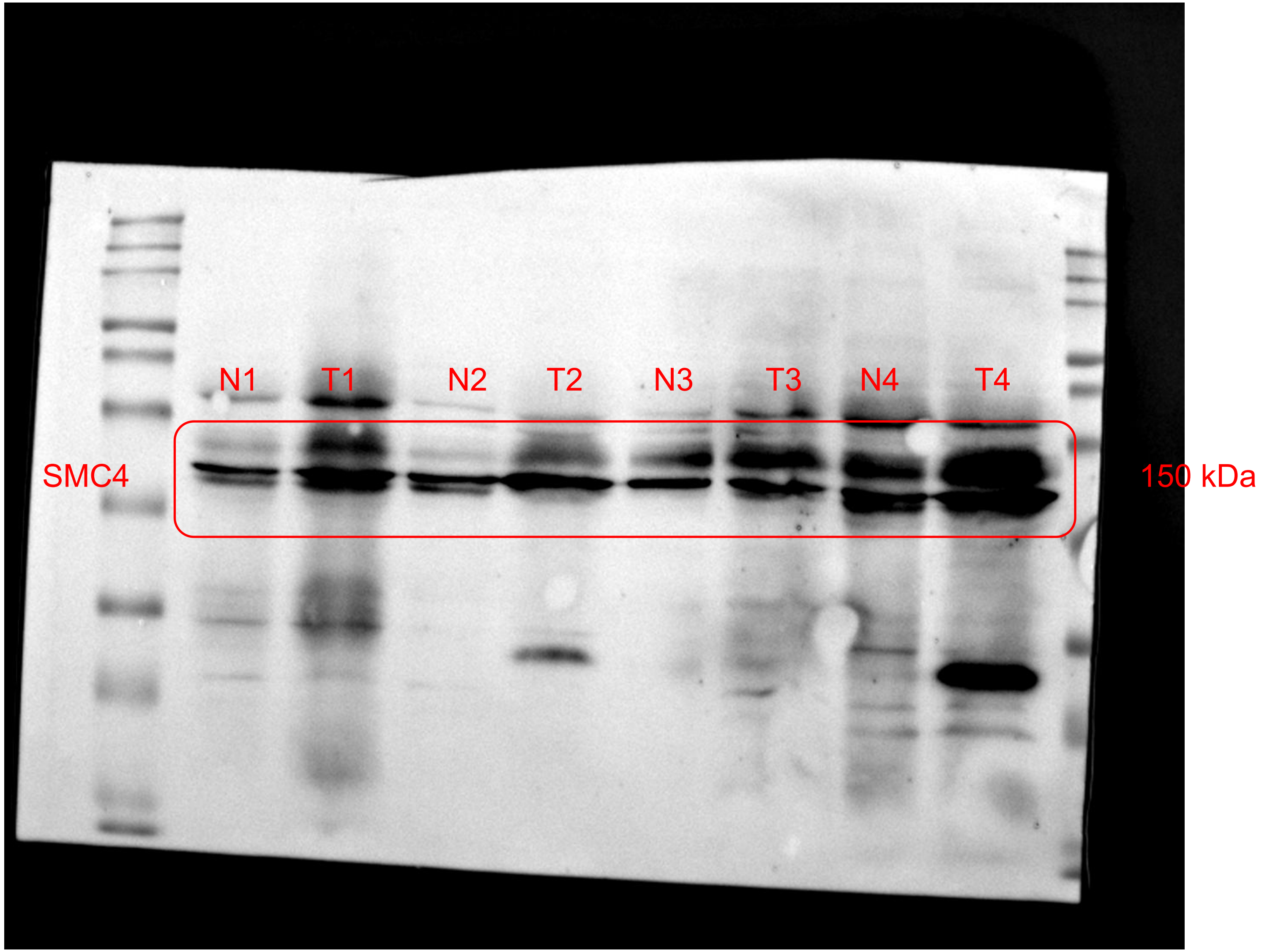

Figure 1B

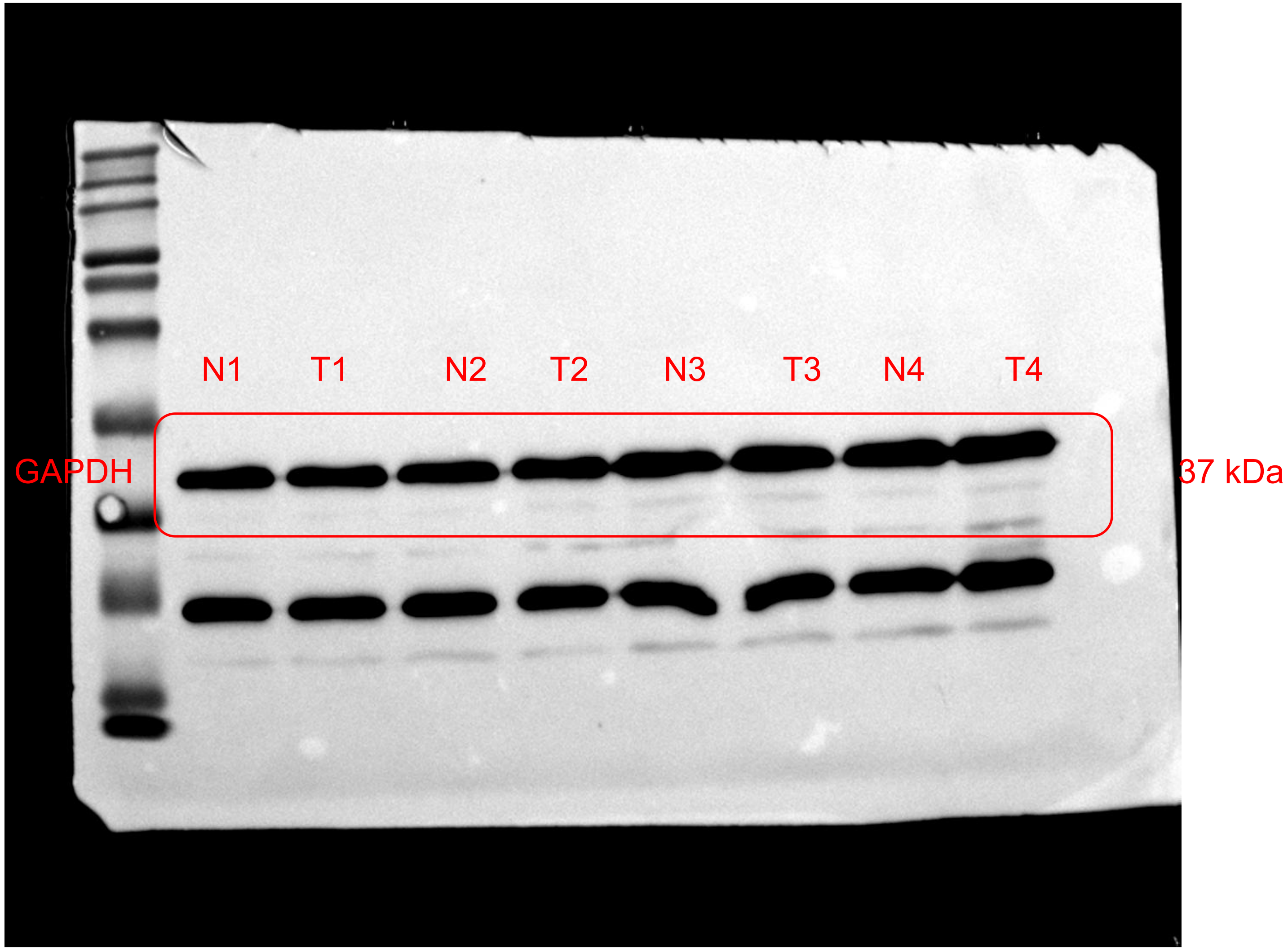

Figure 2A

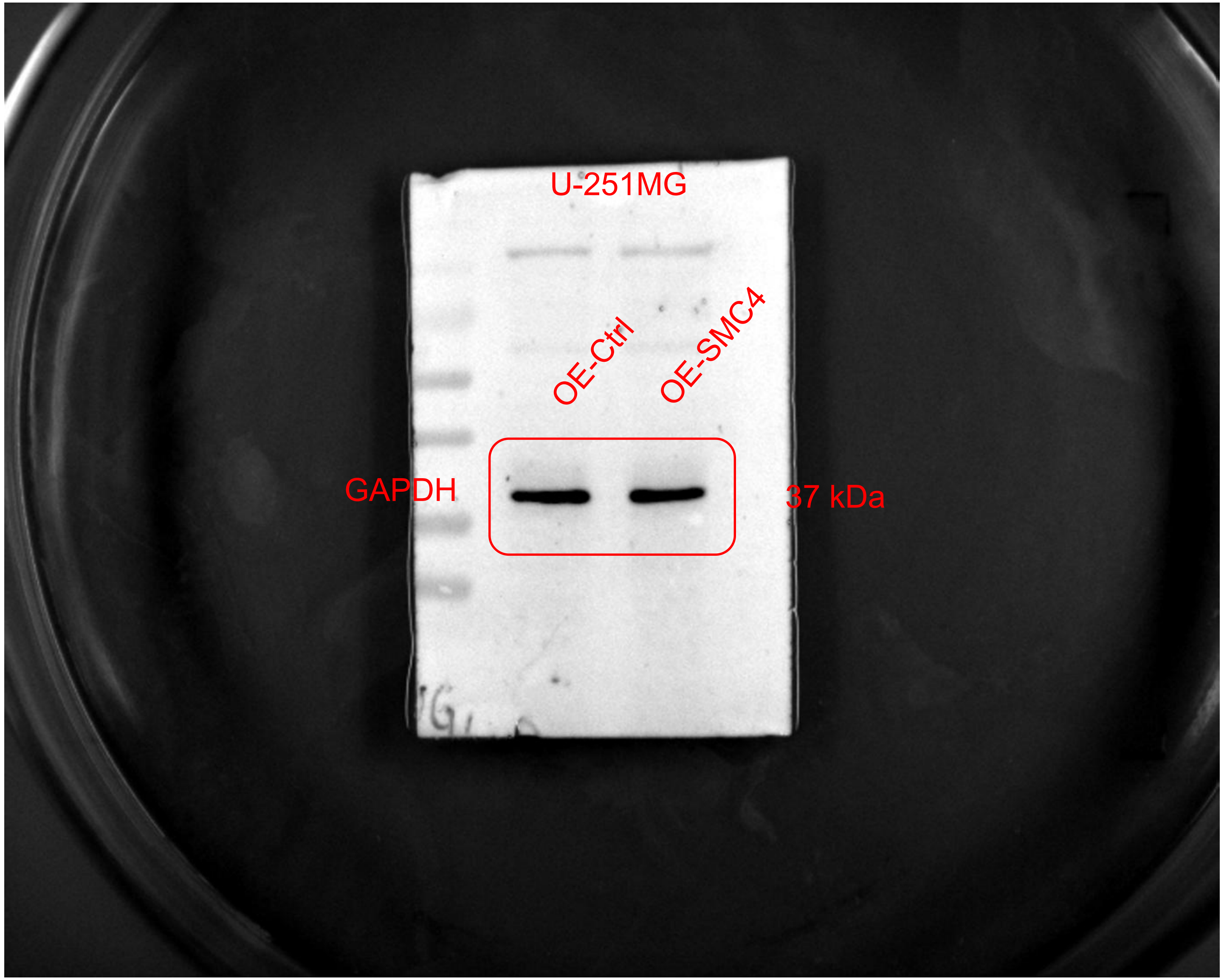

Figure 4H

U-251MG

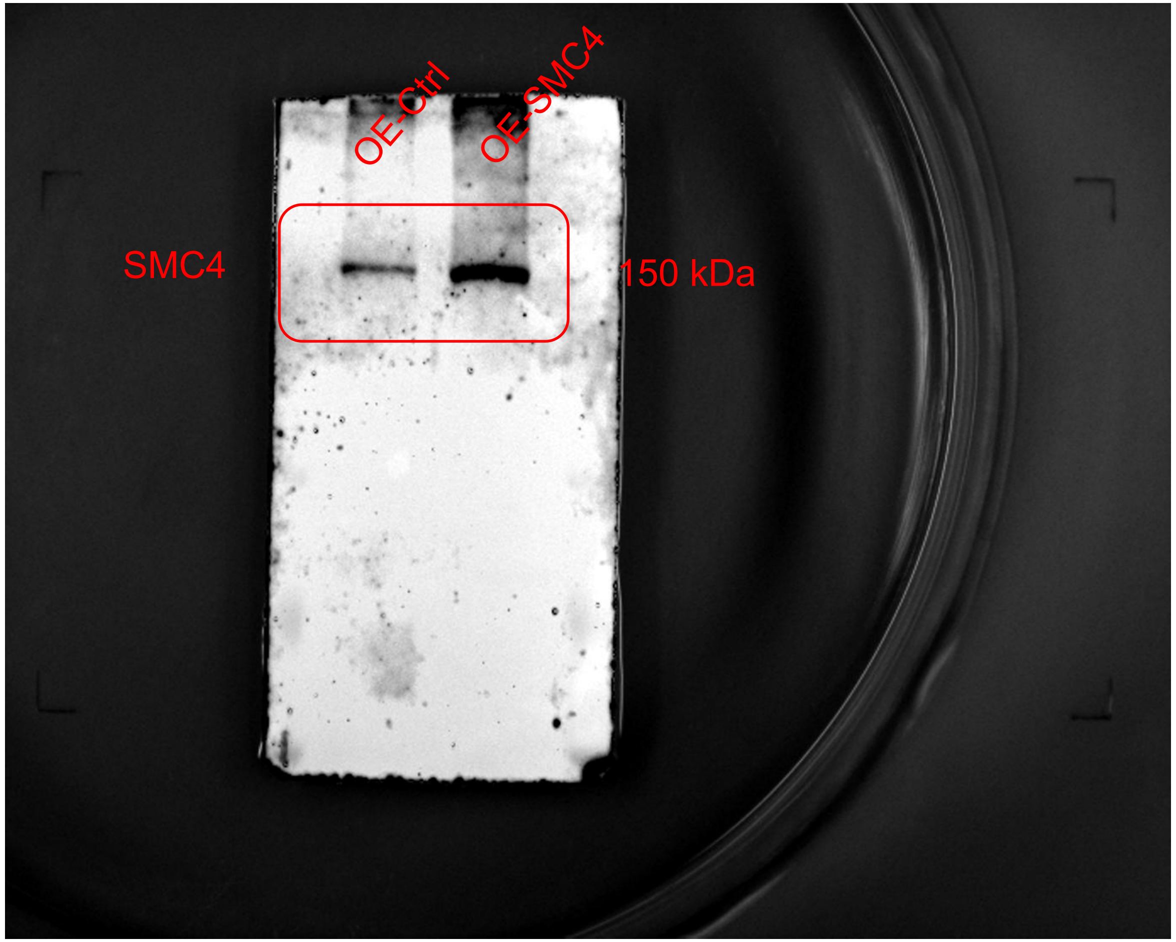

Figure 4H

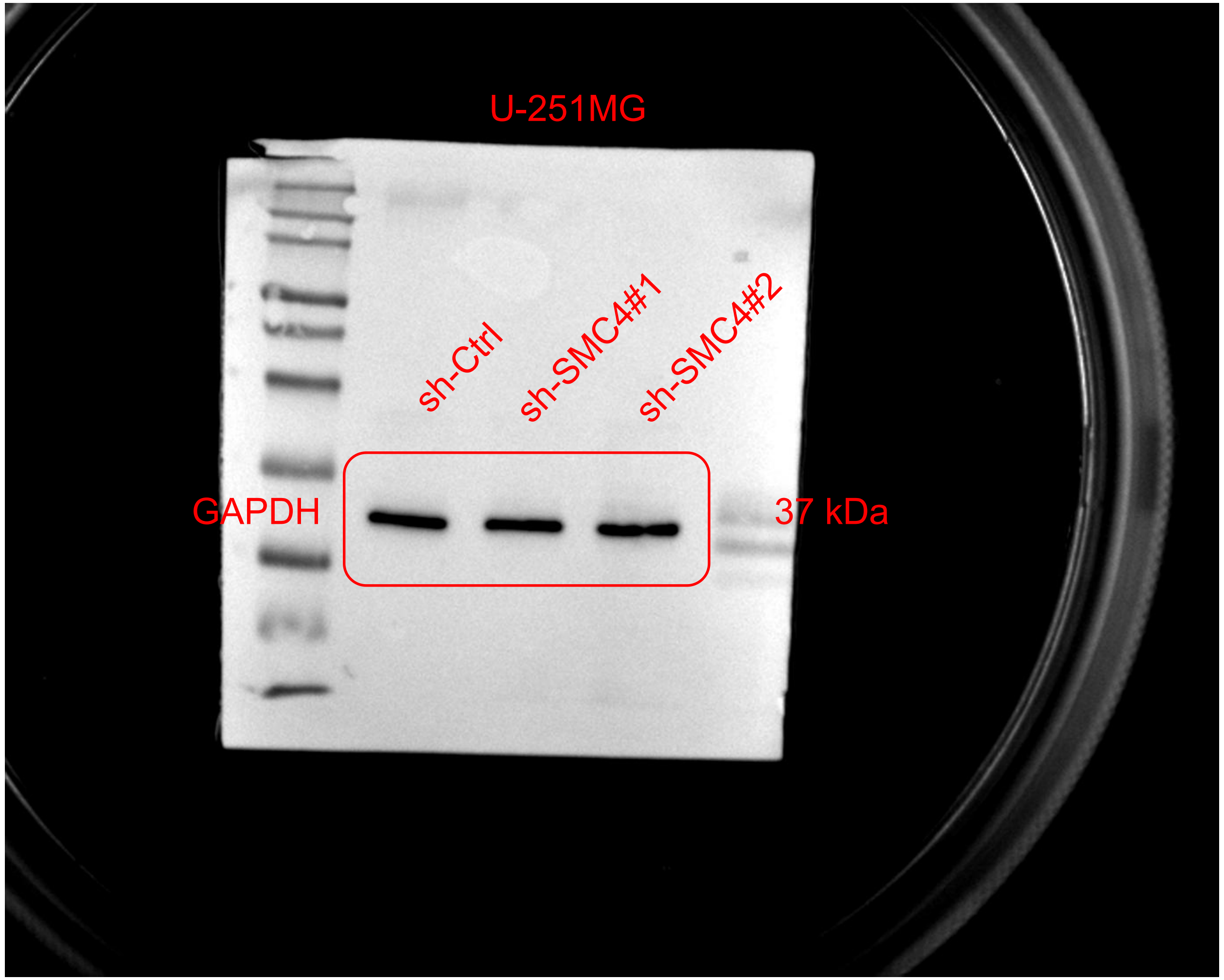

Figure 4H

U-251MG

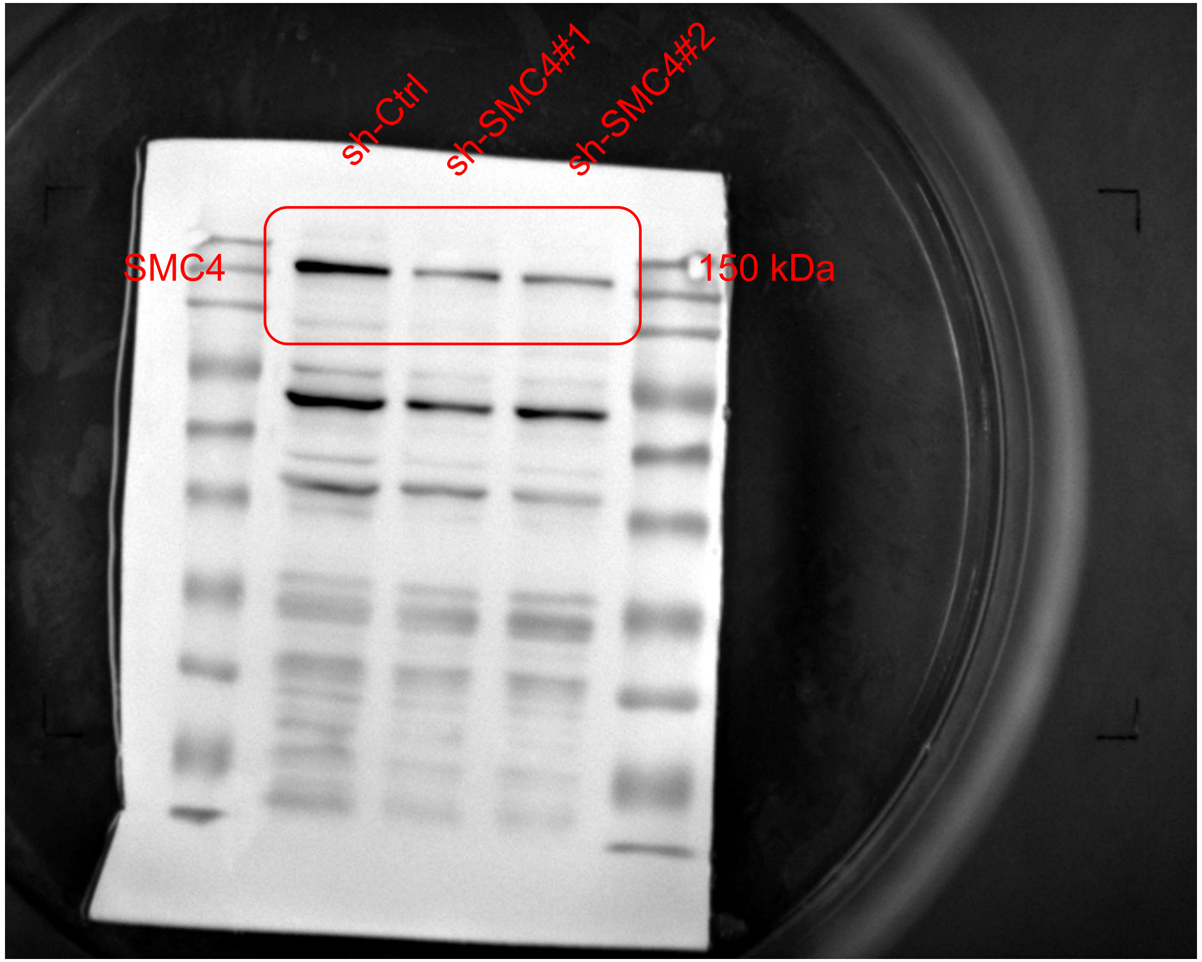

Figure 4H

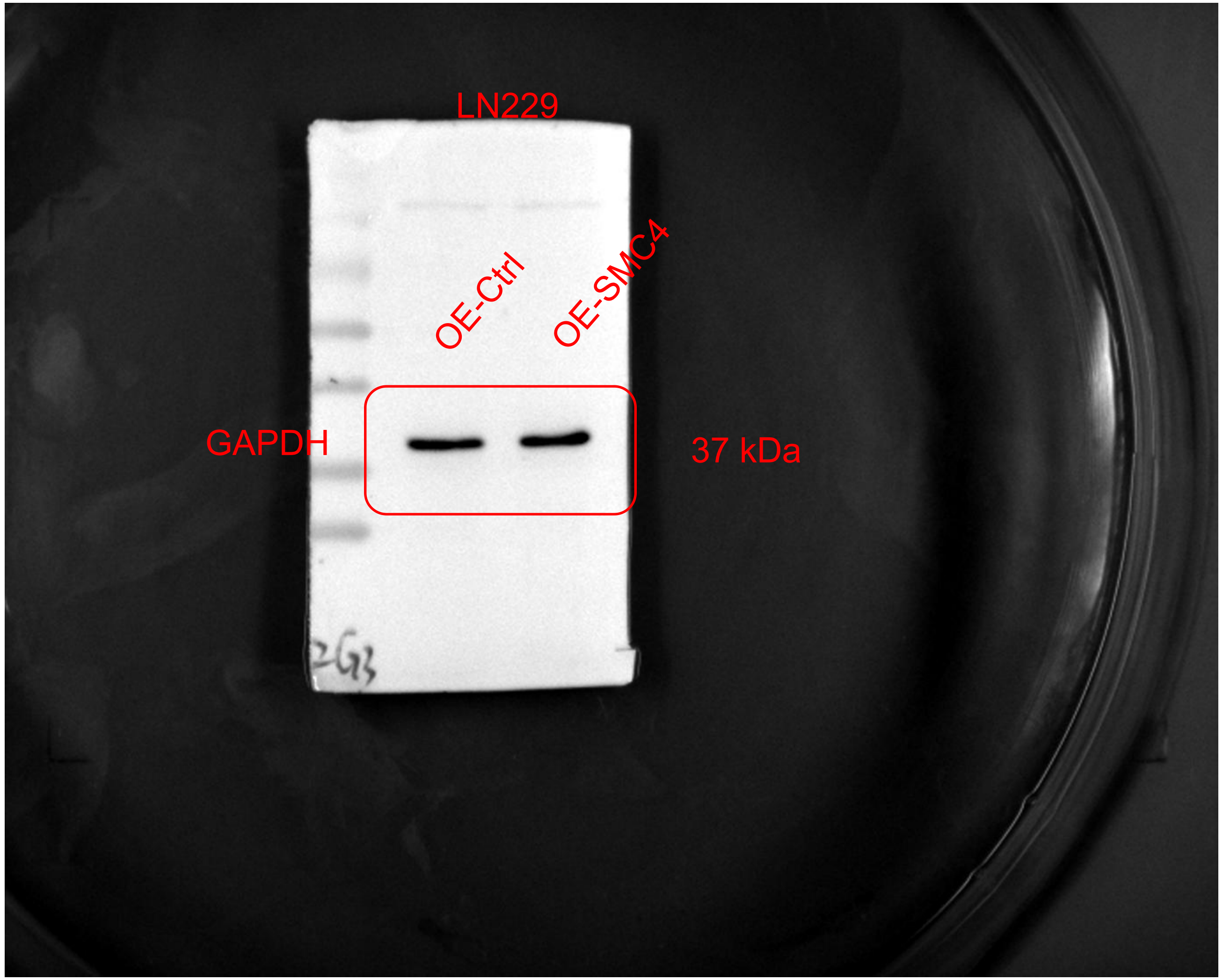

Figure 4H

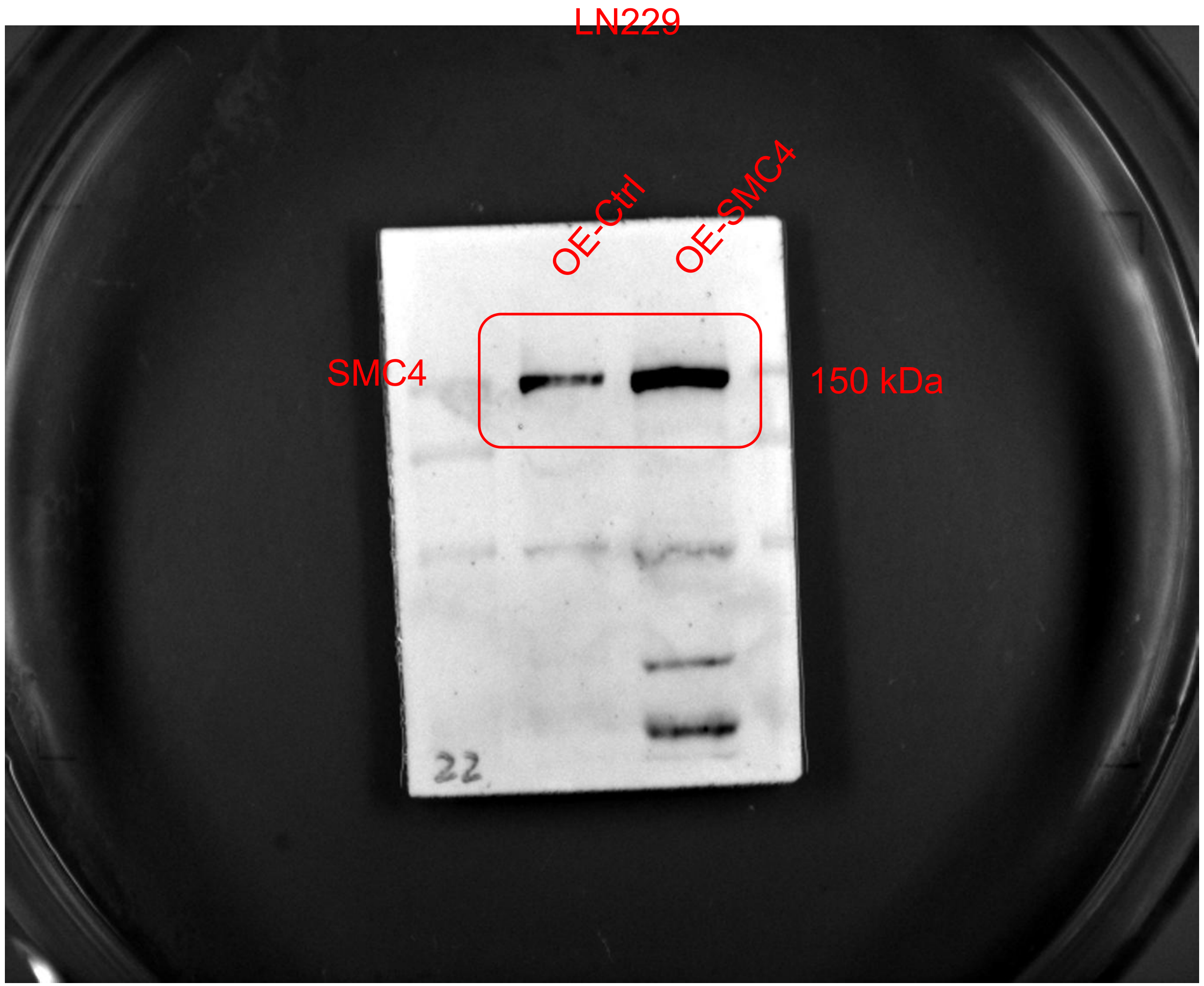

Figure 4H

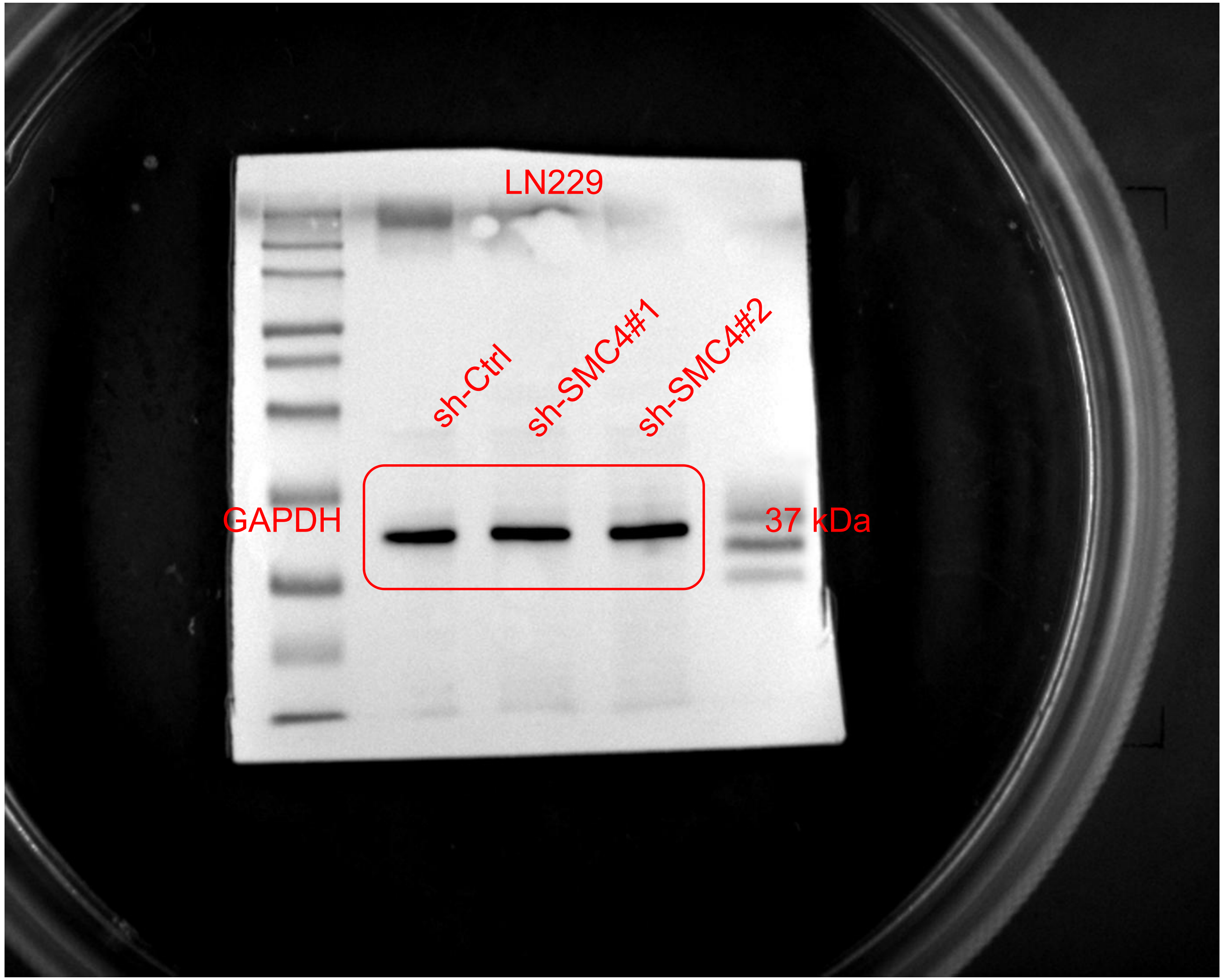

Figure 4H

LN229

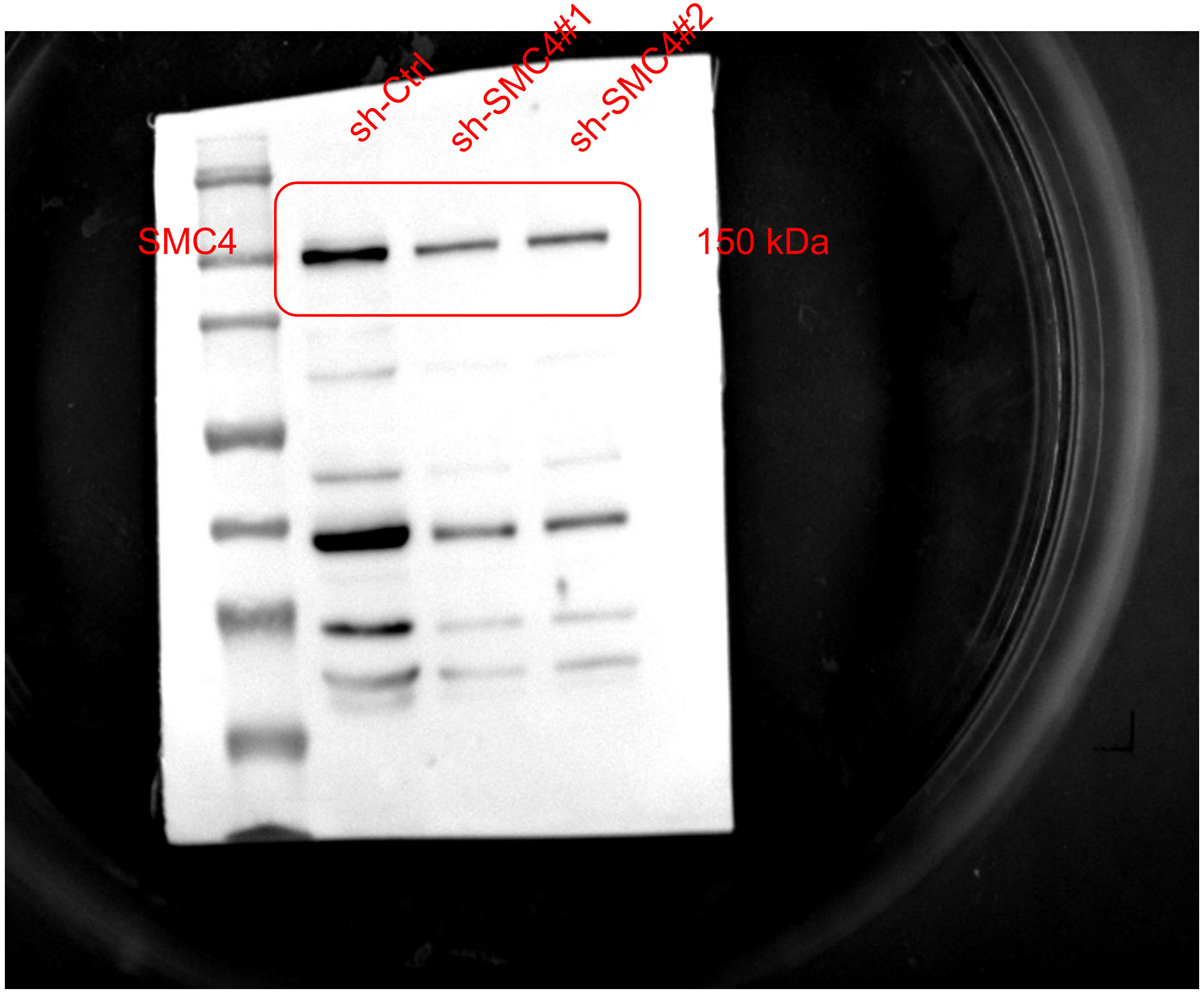

Figure 4H

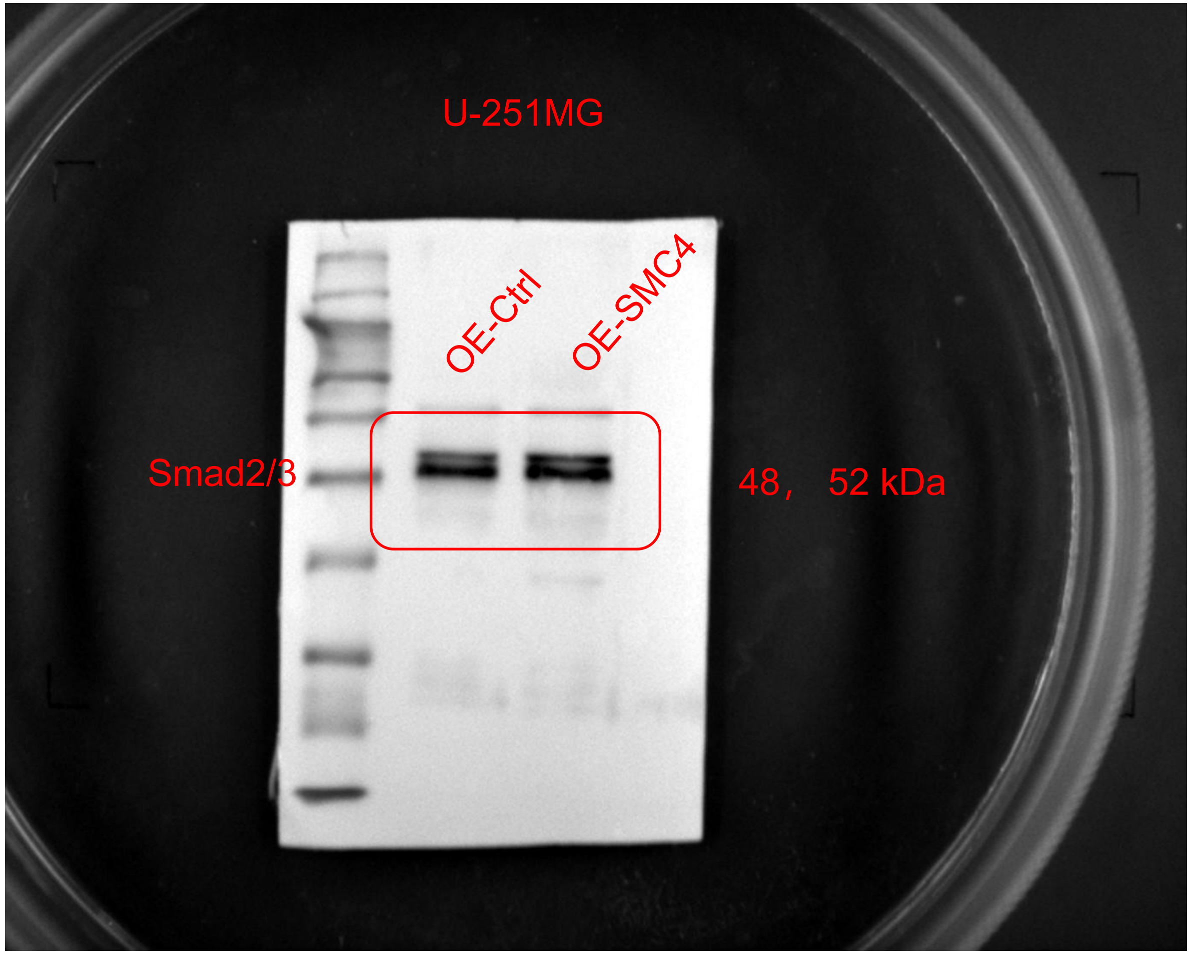

Figure 4H

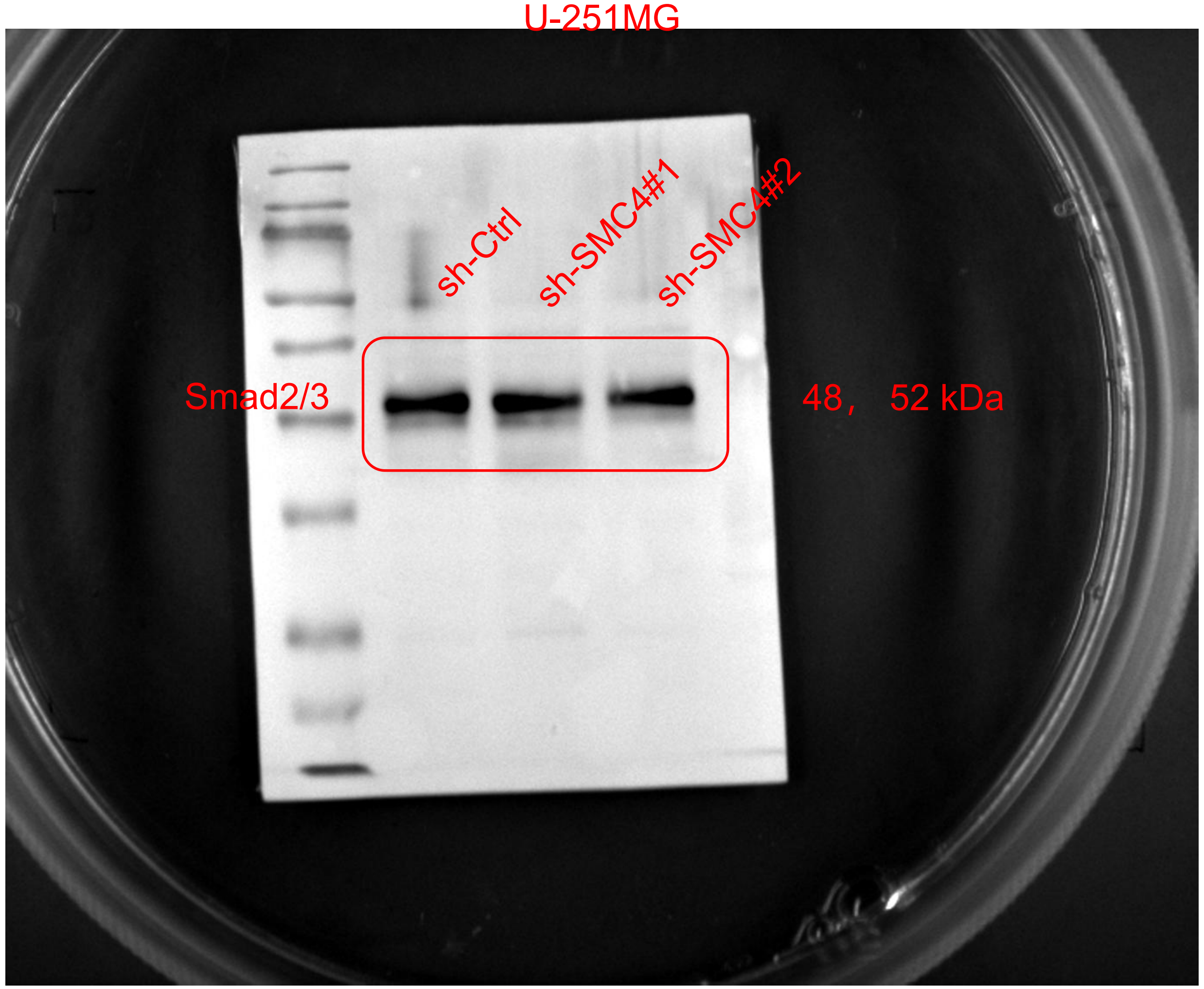

Figure 4H

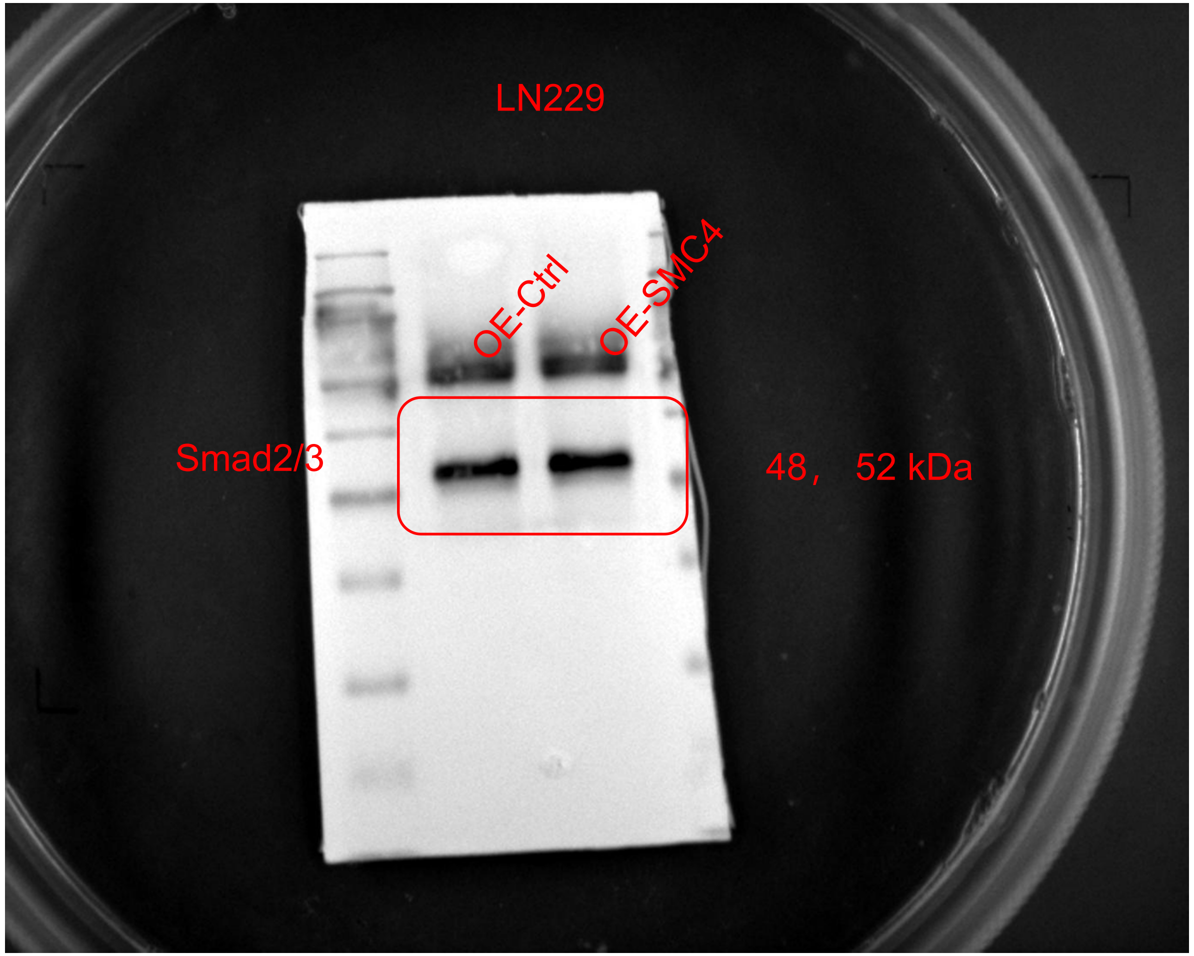

Figure 4H

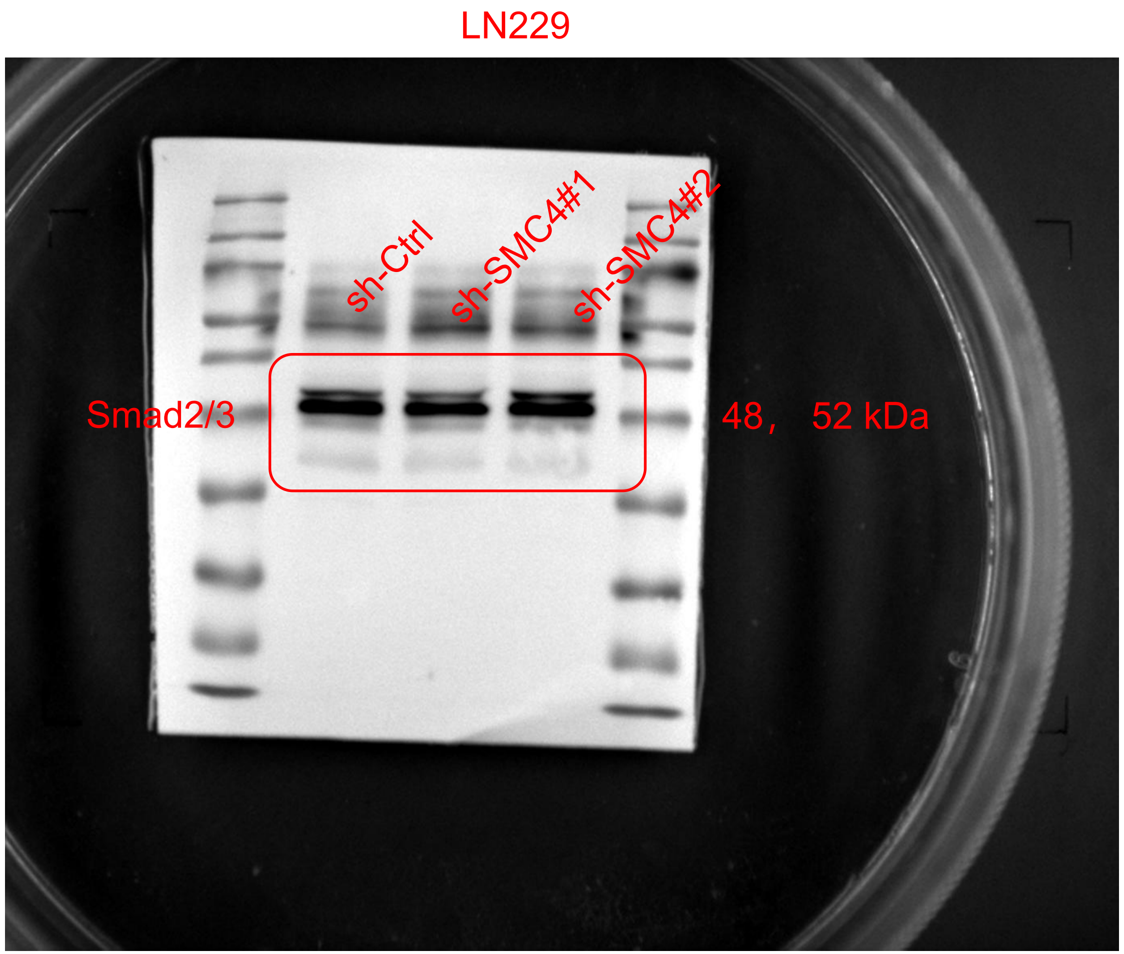

Figure 4H

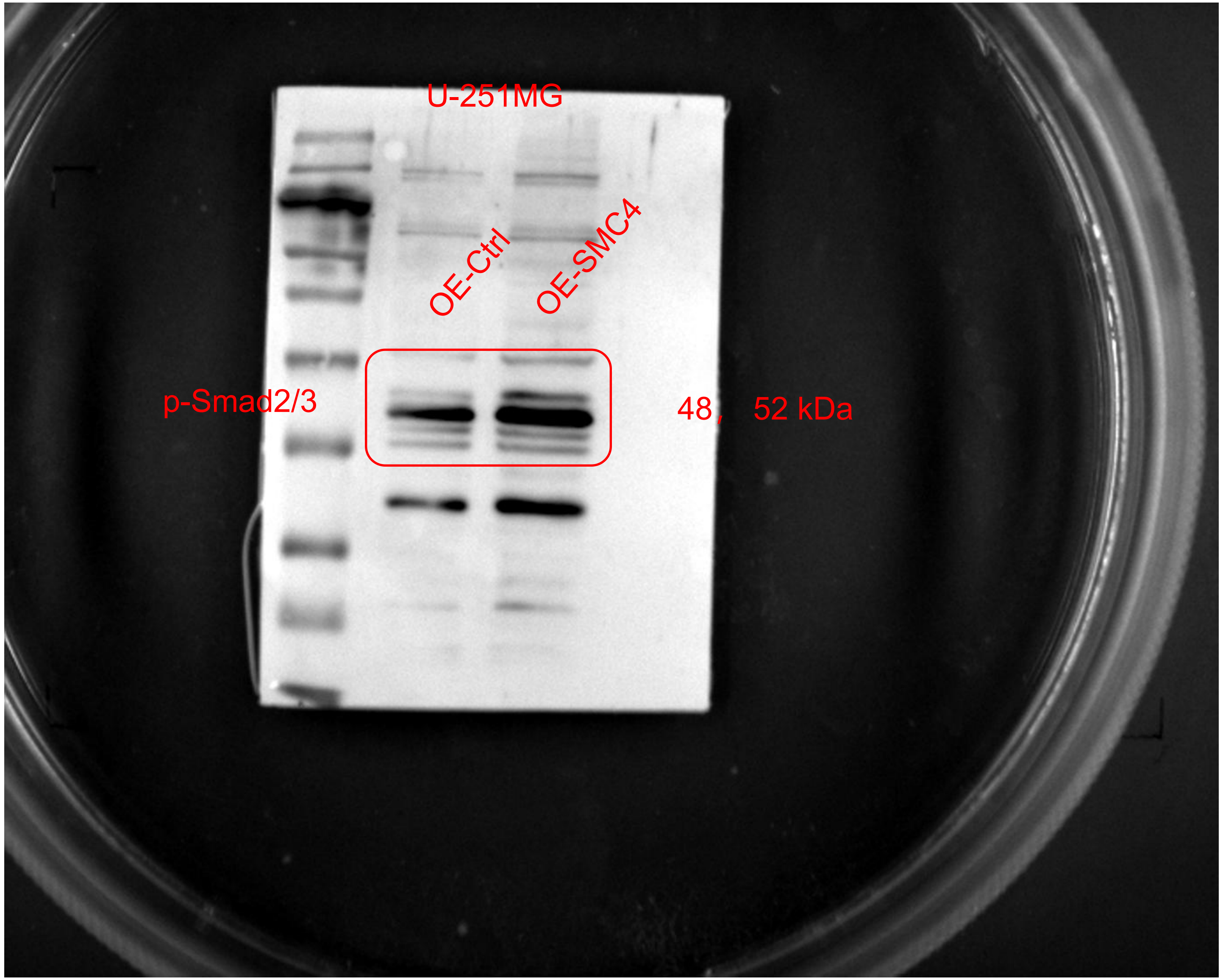

Figure 4H

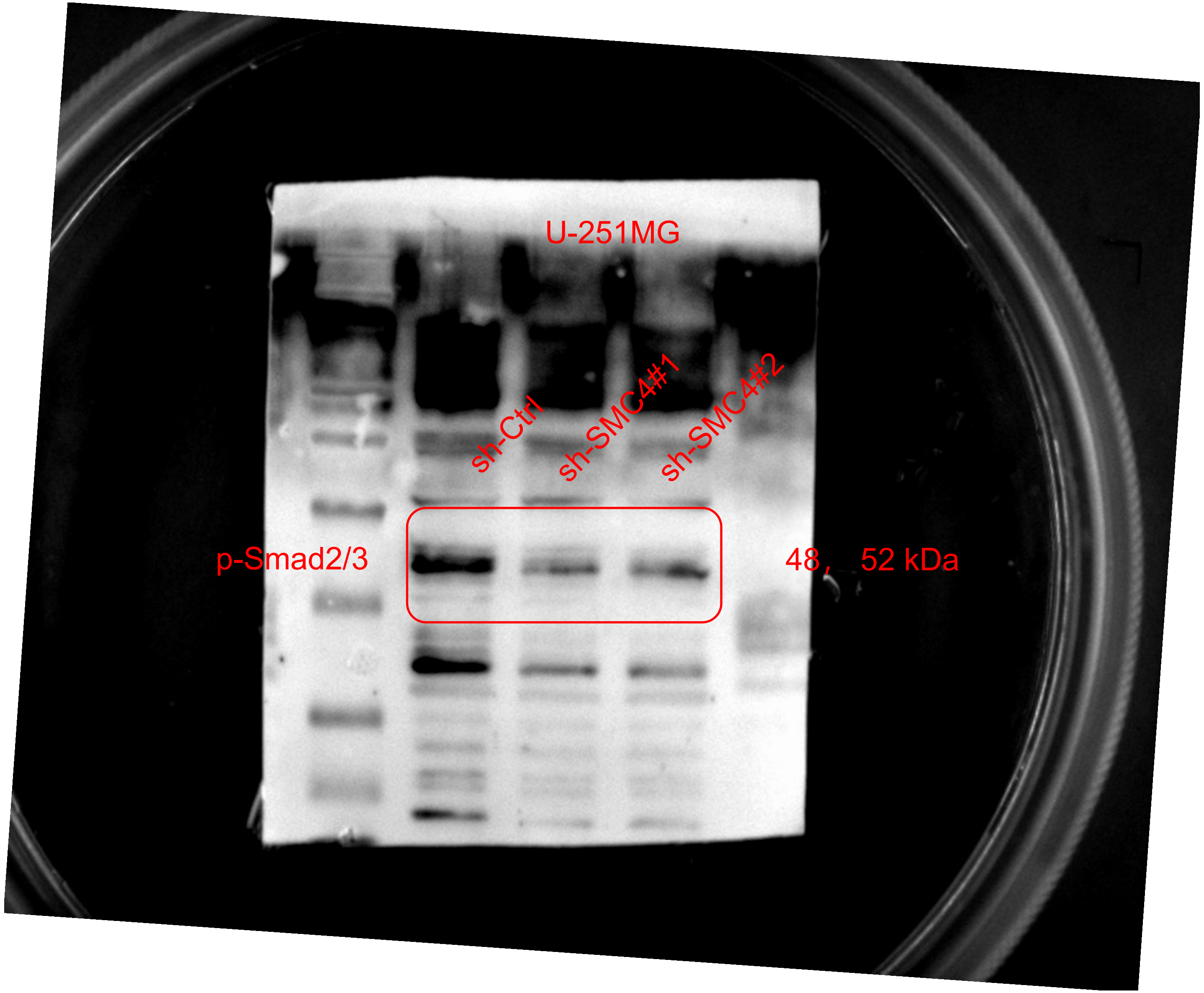

Figure 4H

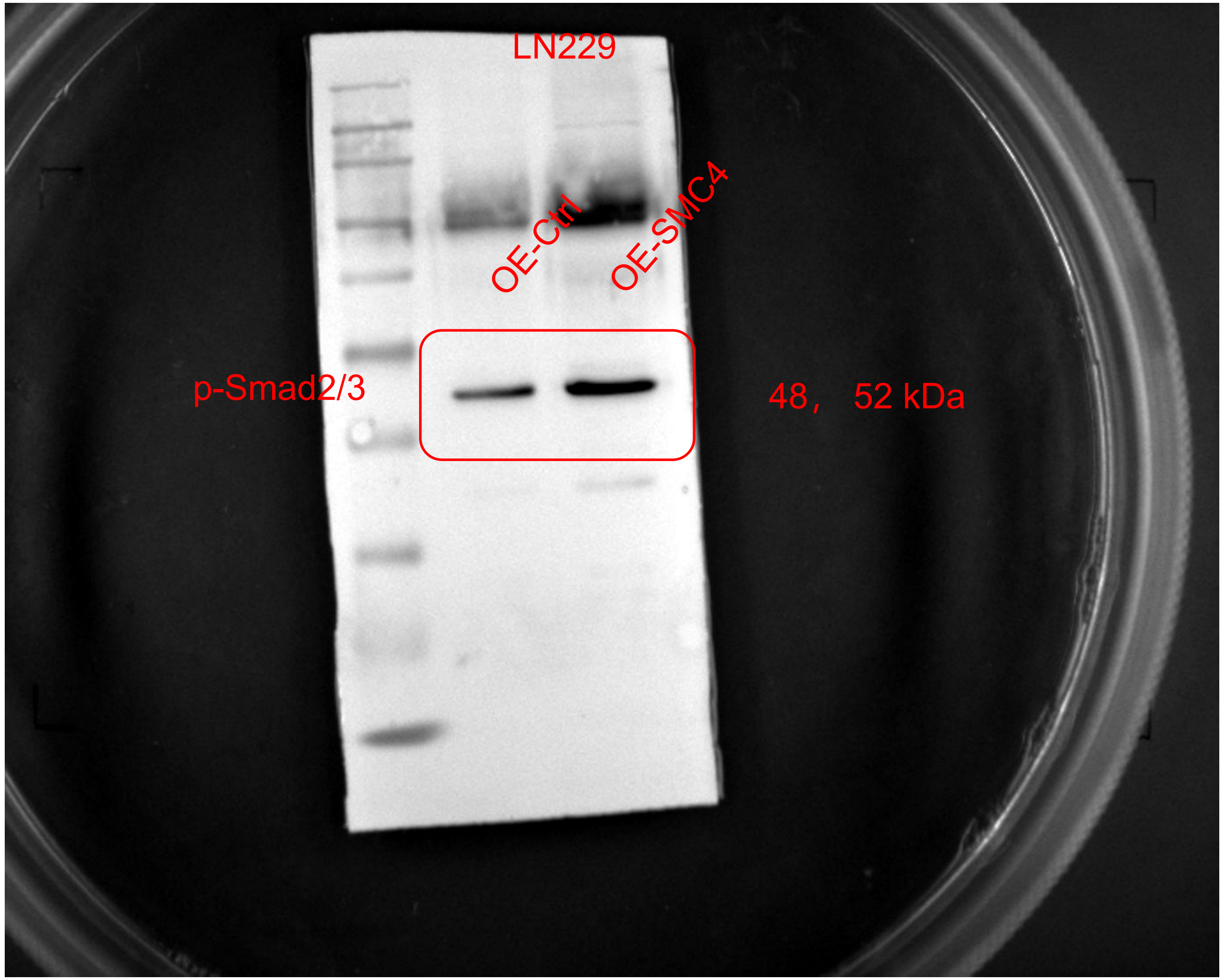

Figure 4H

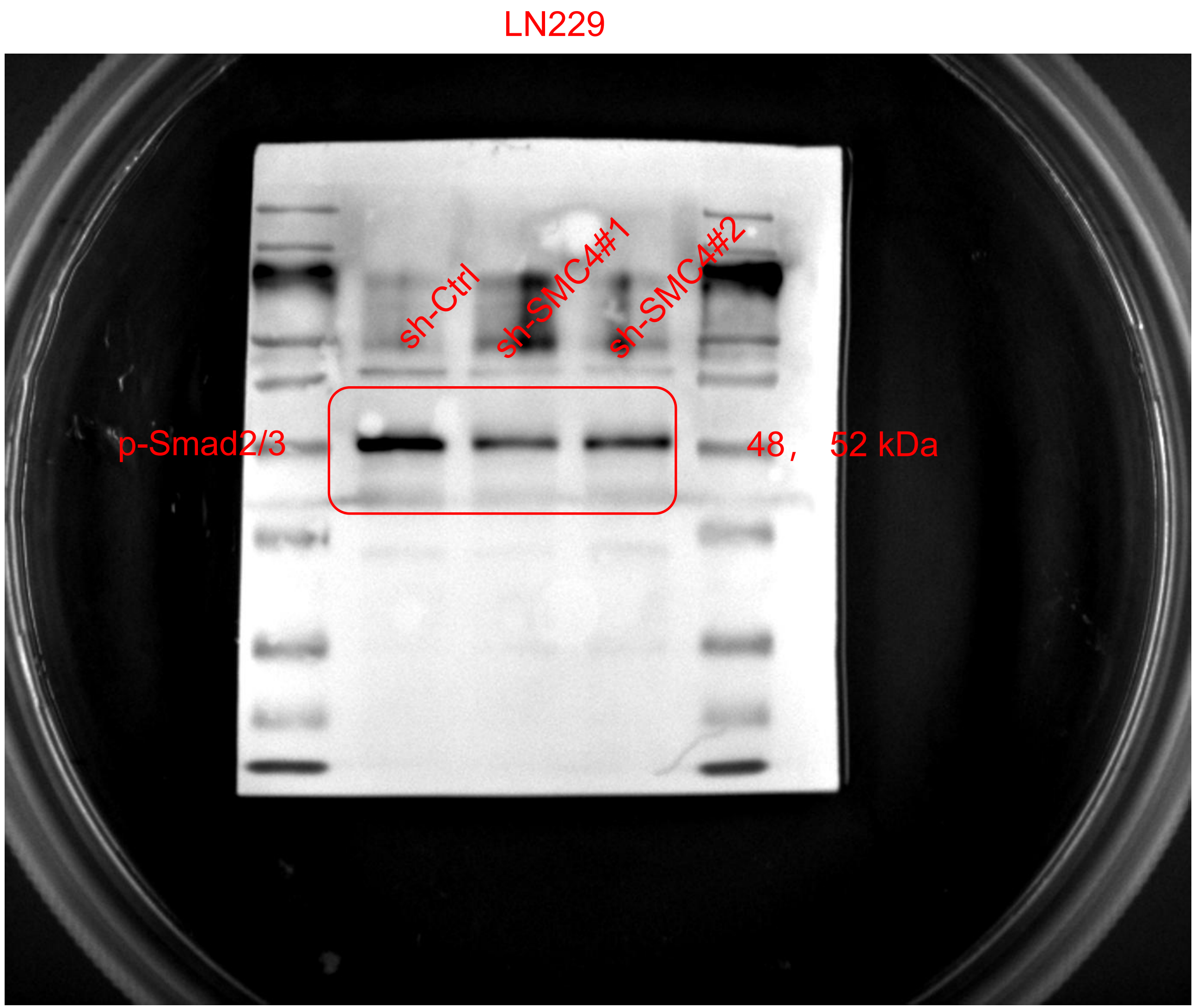

Figure 4H

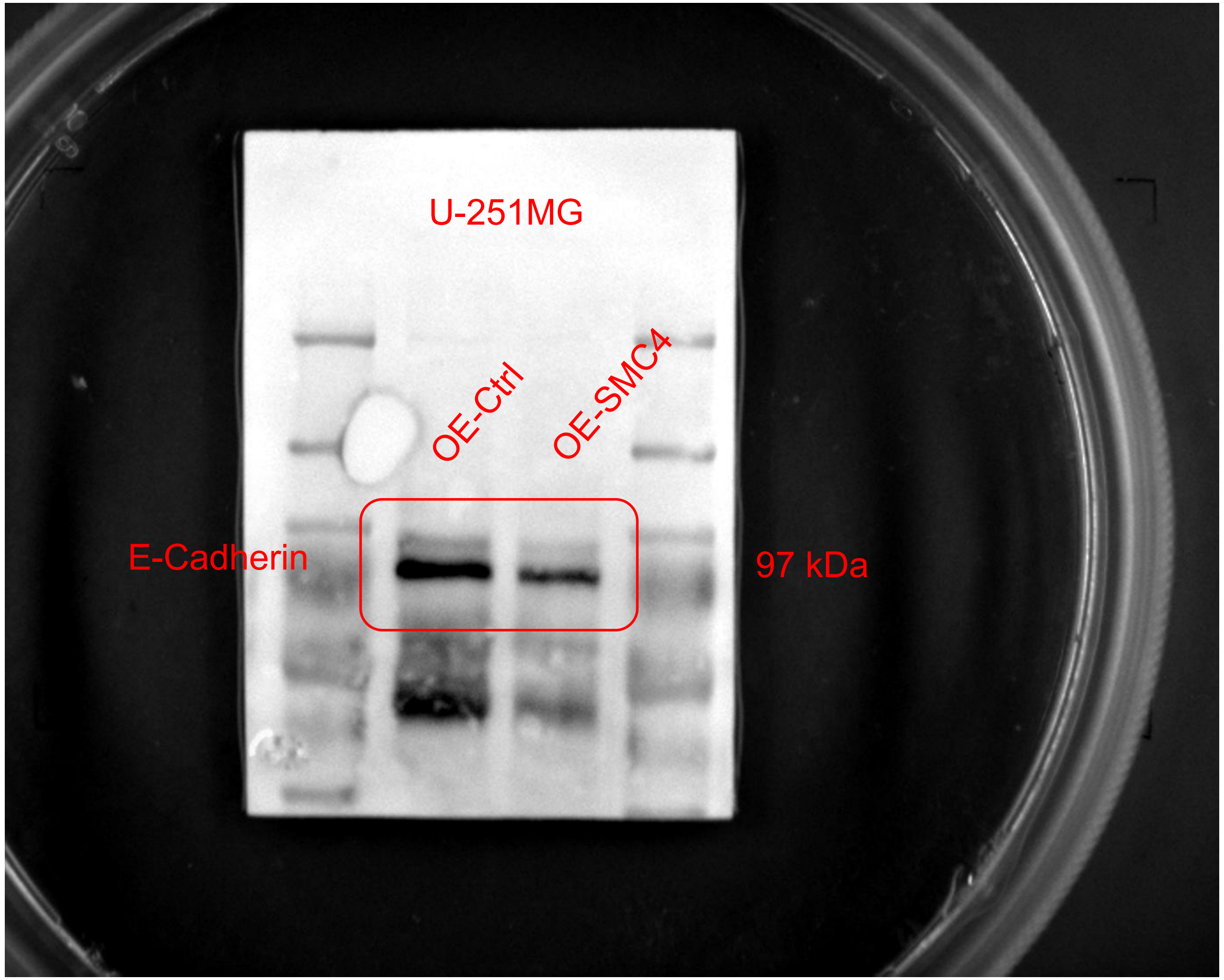

Figure 4H

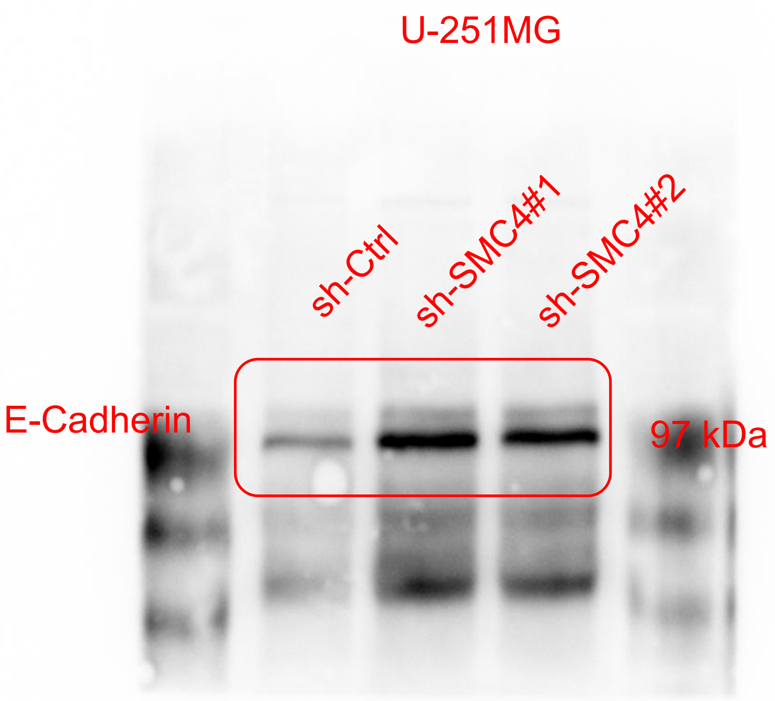

Figure 4H

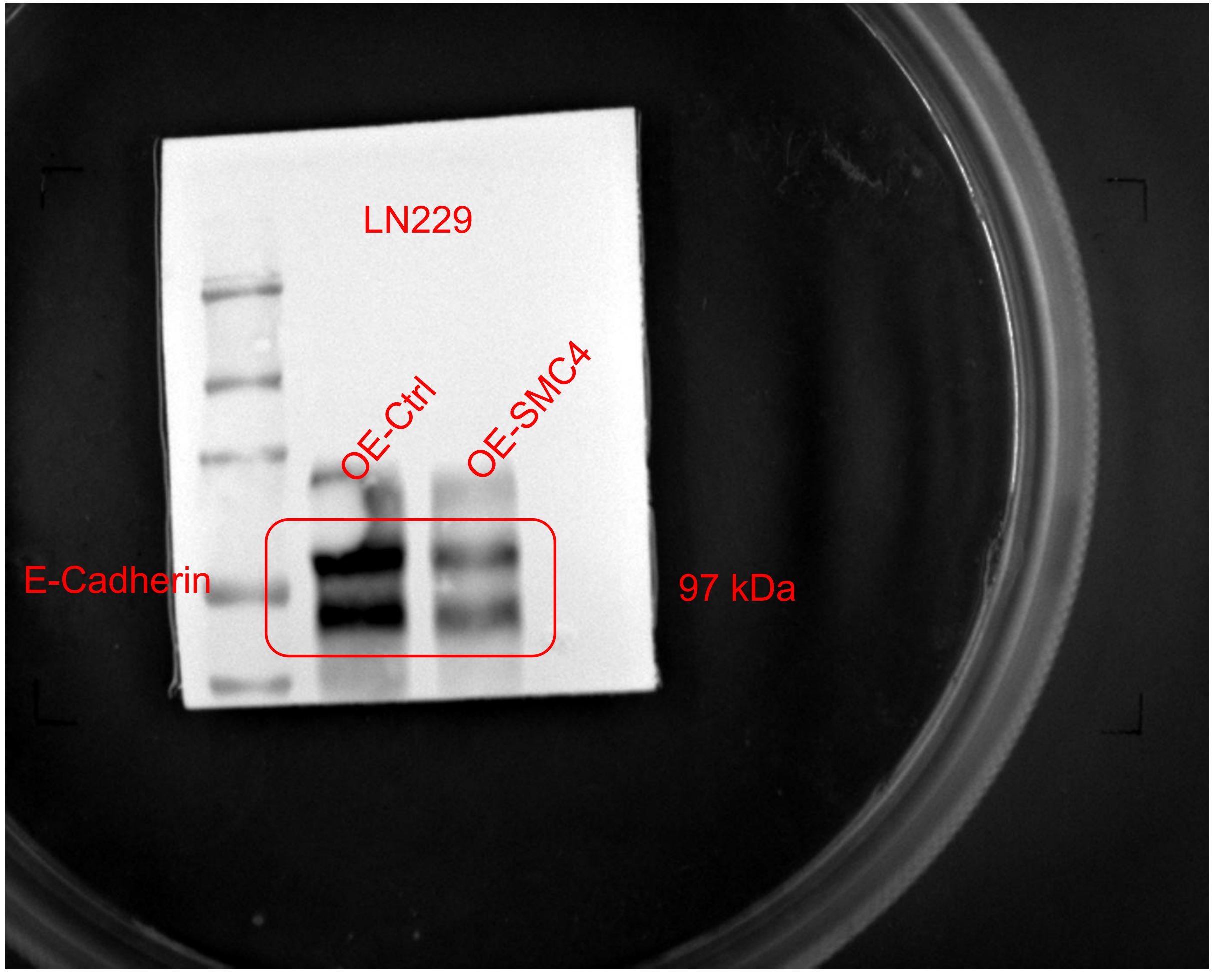

Figure 4H

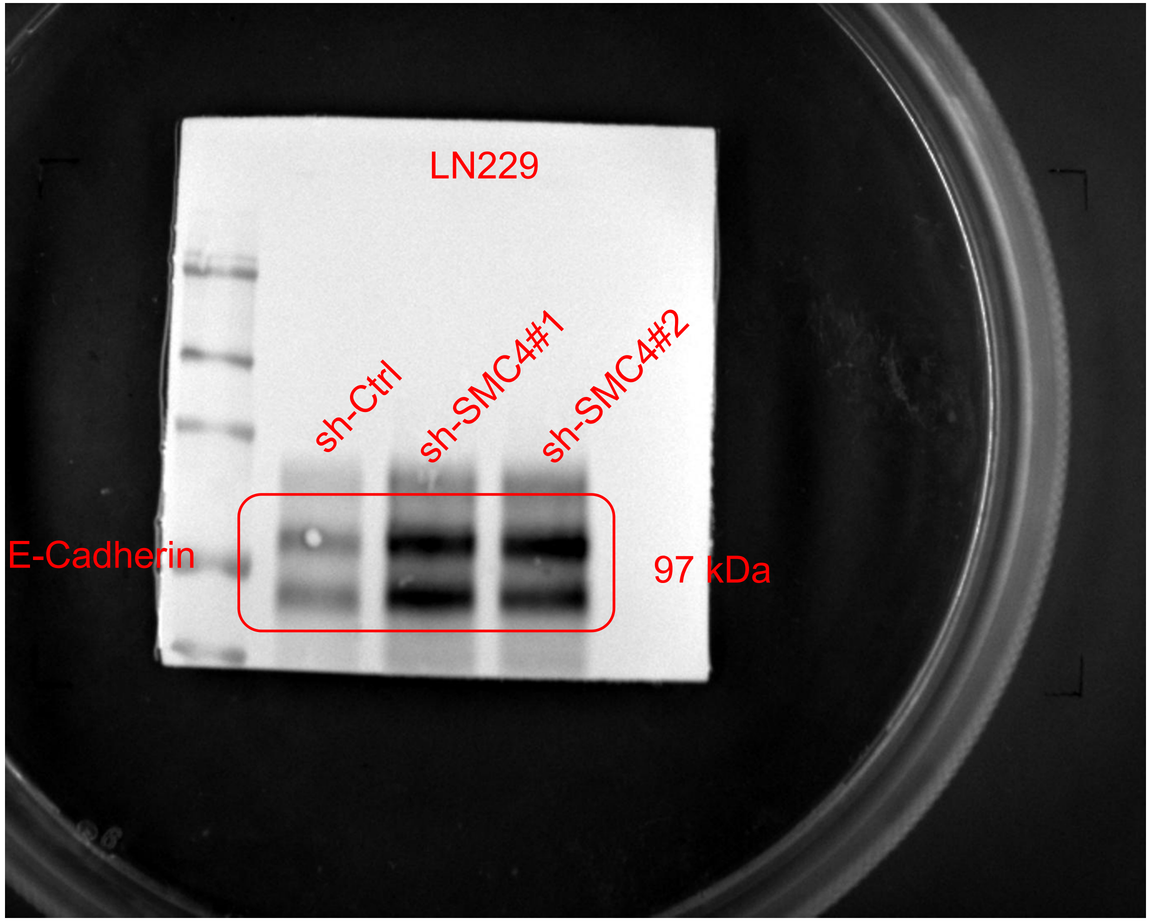

Figure 4H

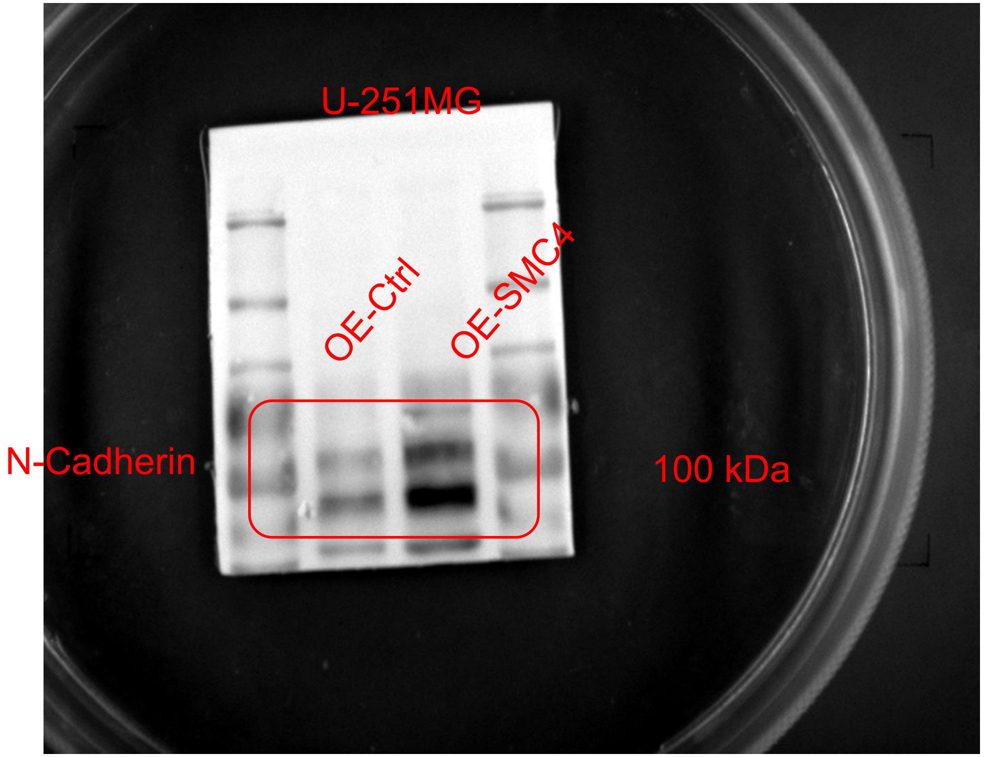

Figure 4H

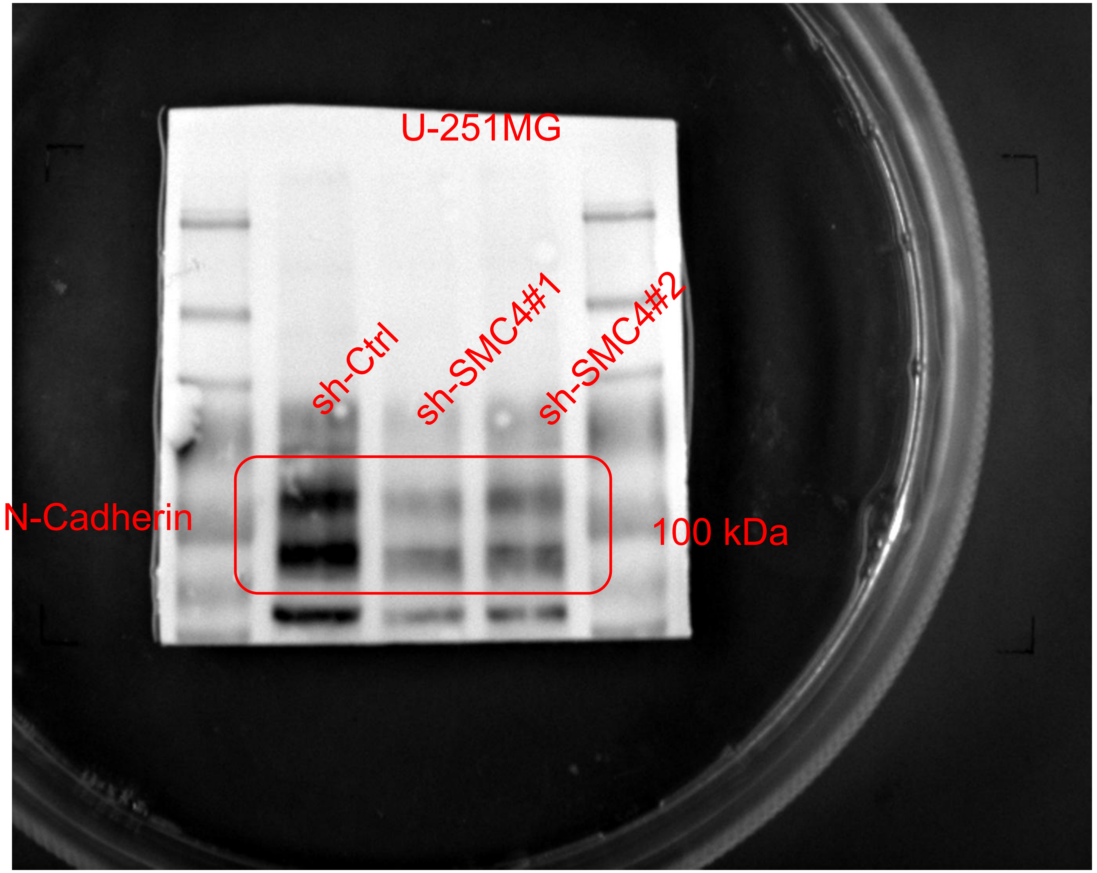

Figure 4H

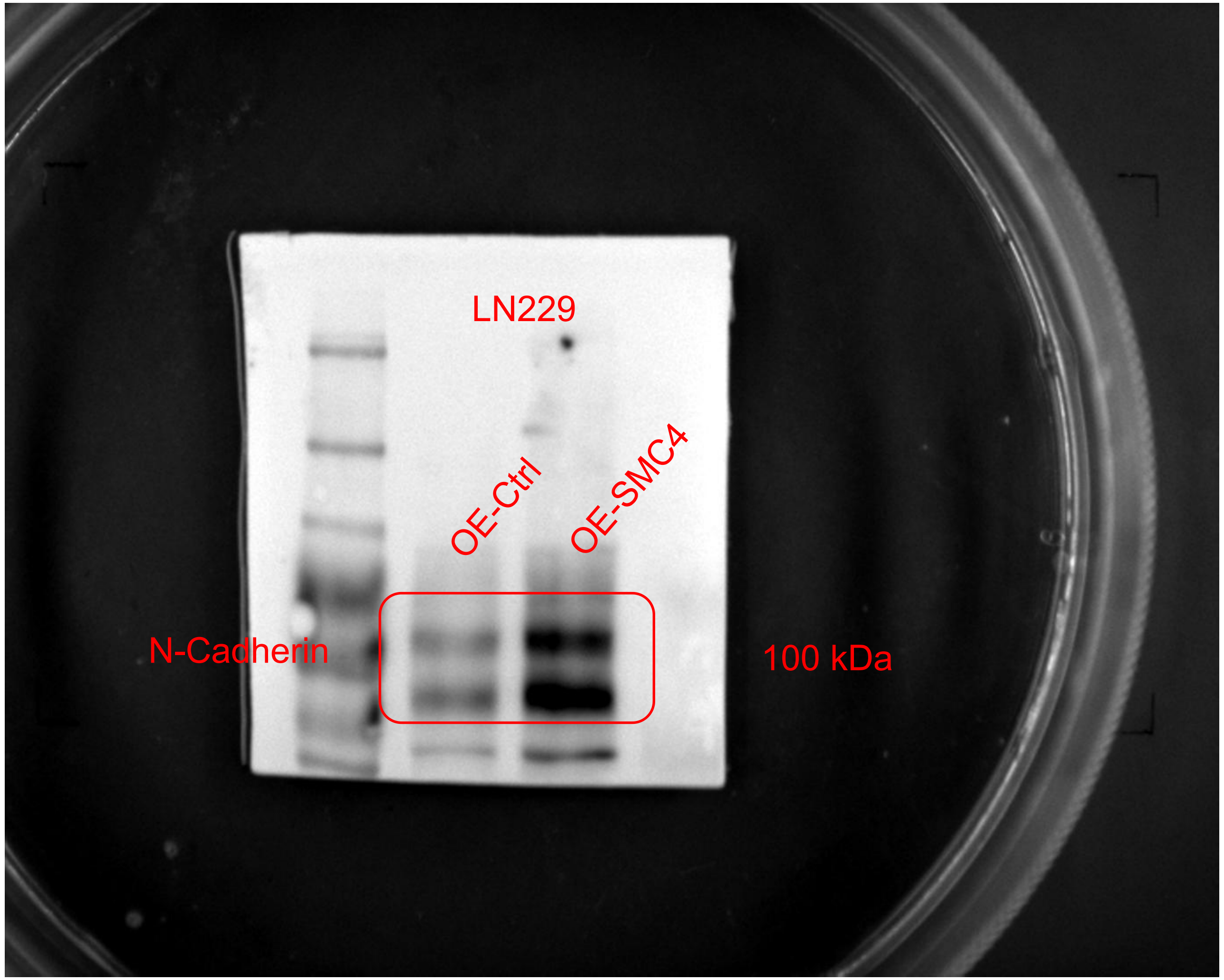

Figure 4H

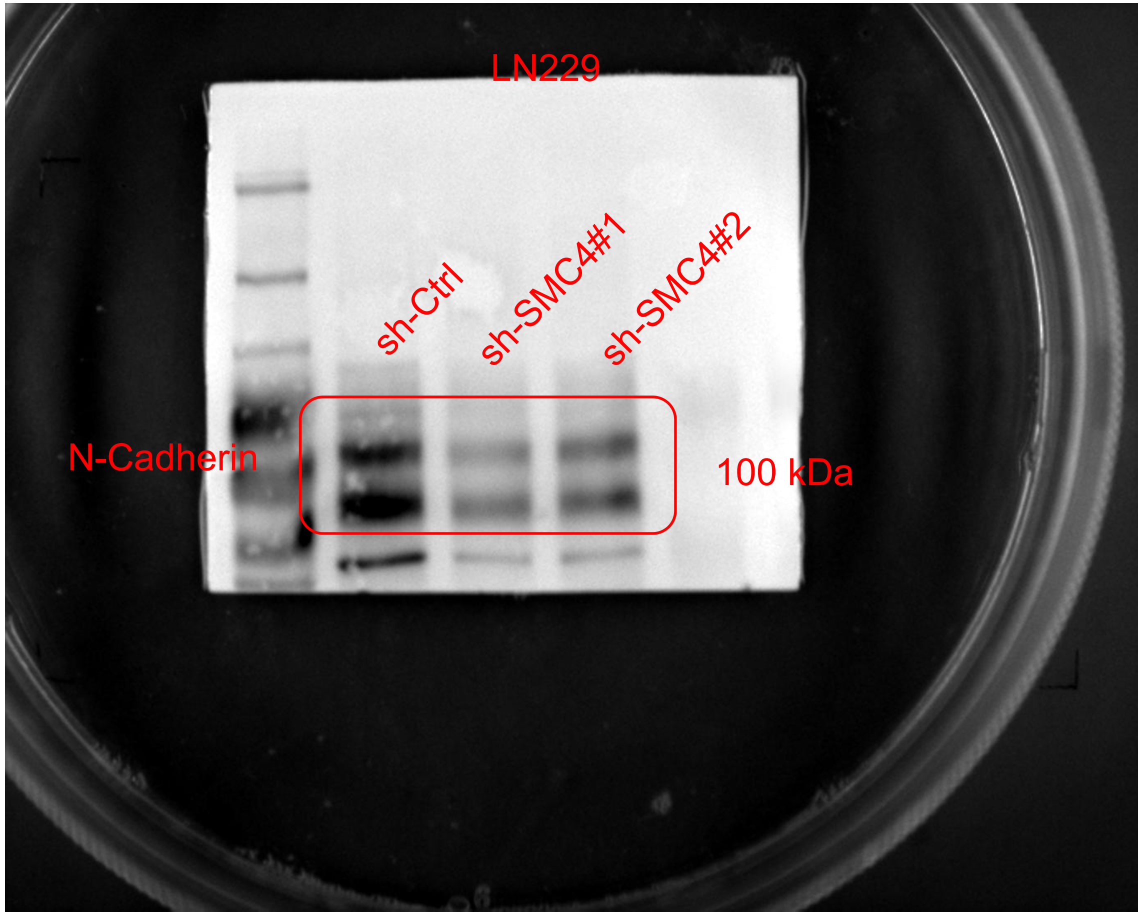

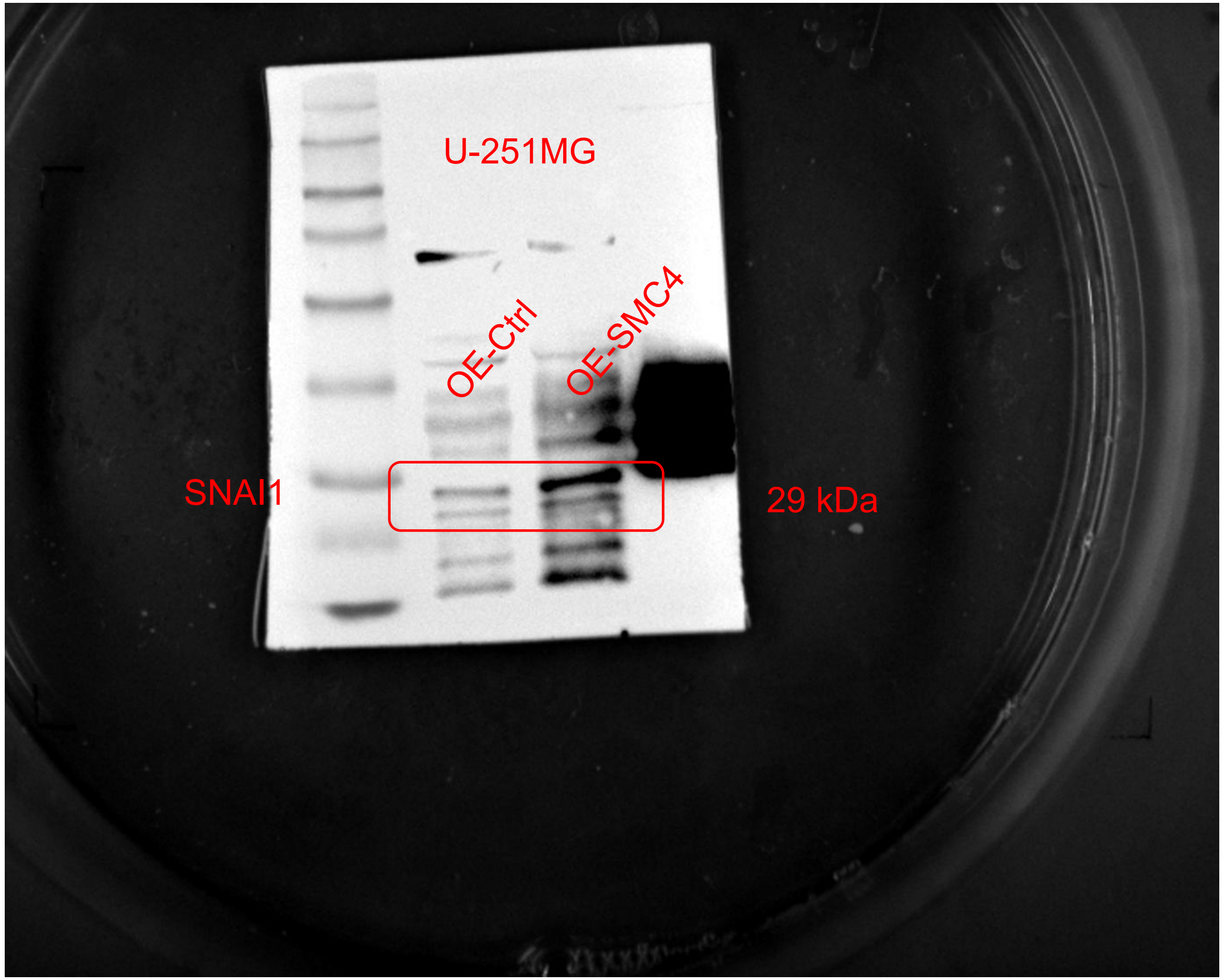
U-251MG
OE-Ctrl
OE-SMC4
SNAI1
29 kDa

Figure 4H

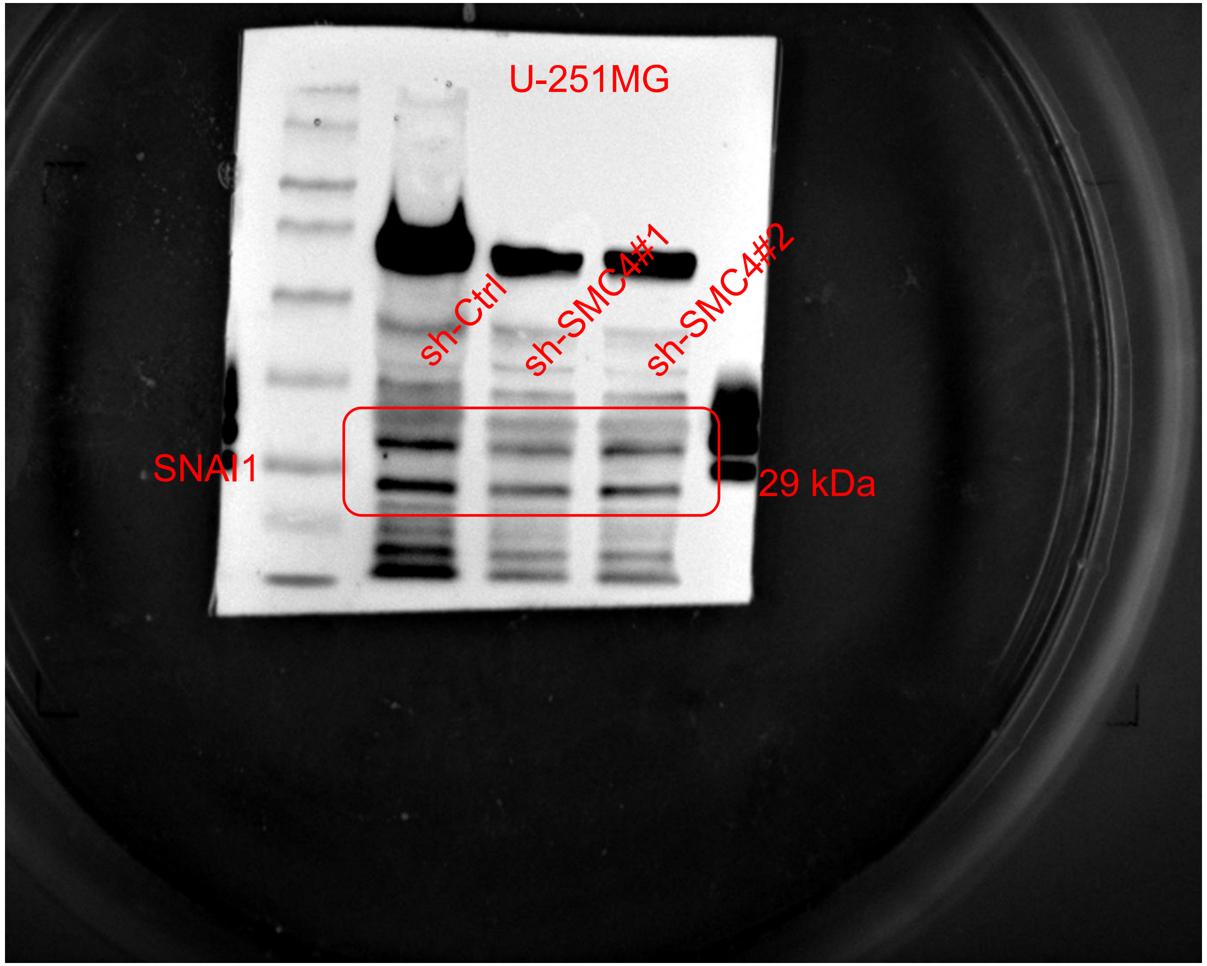

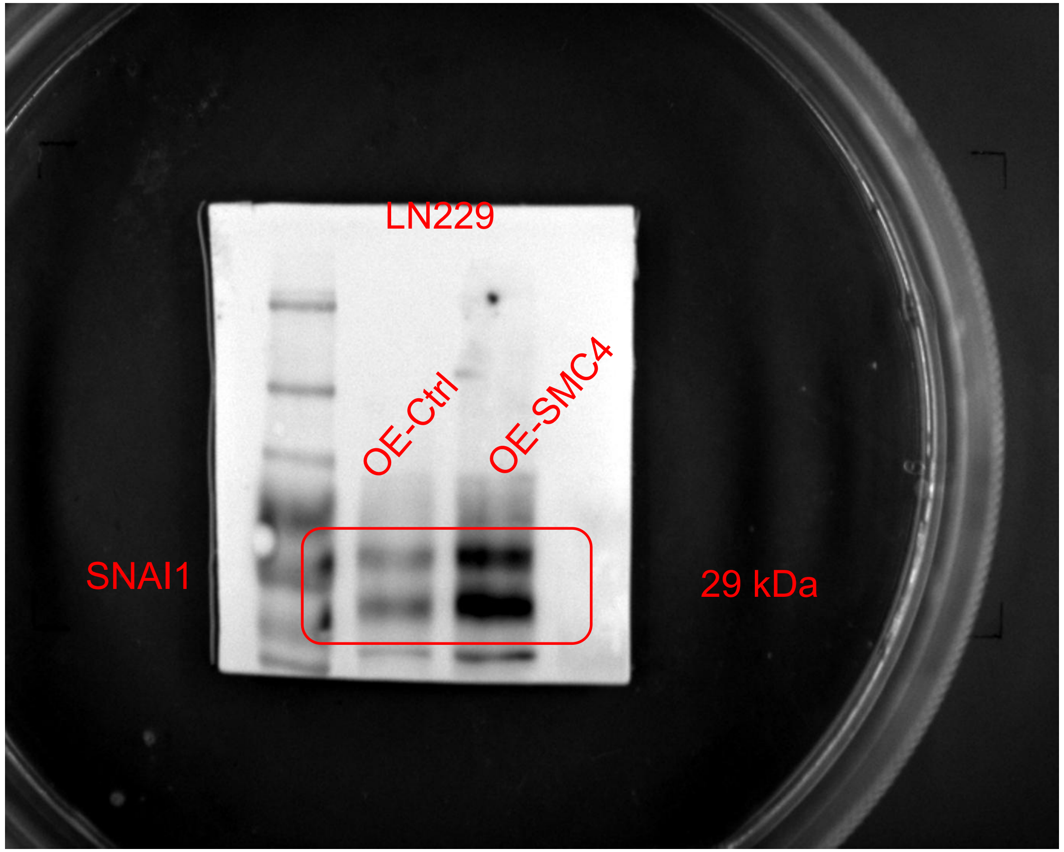
LN229
OE-Ctrl
OE-SMC4
SNAI1
29 kDa

Figure 4H

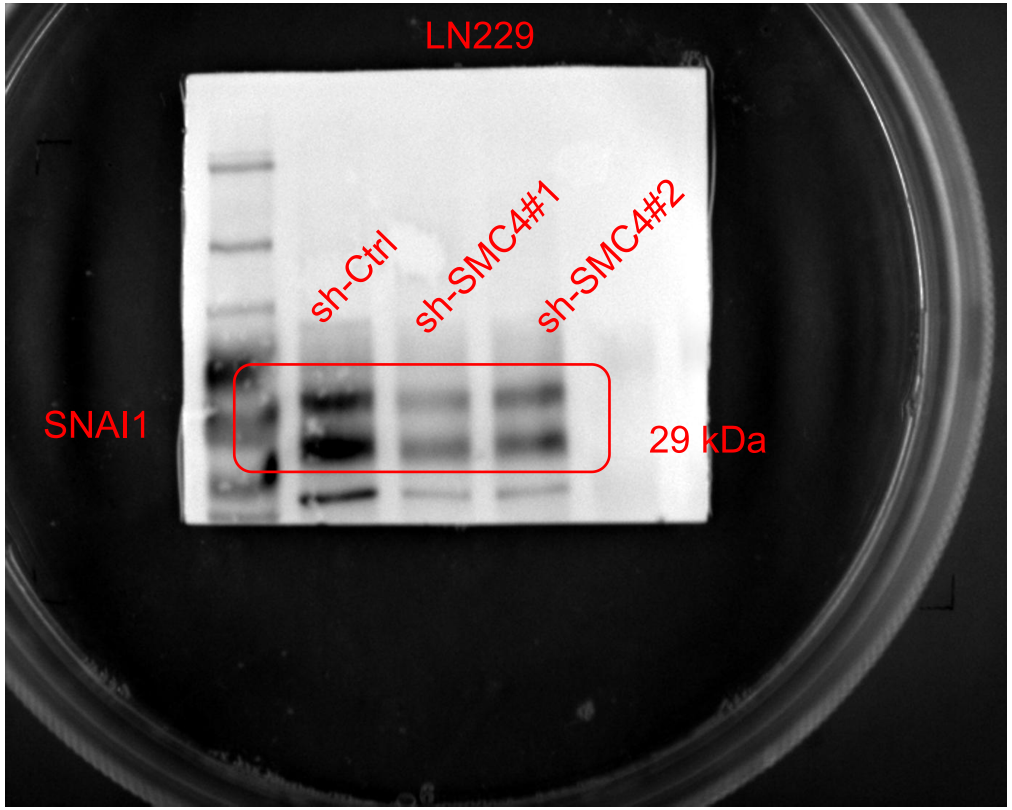

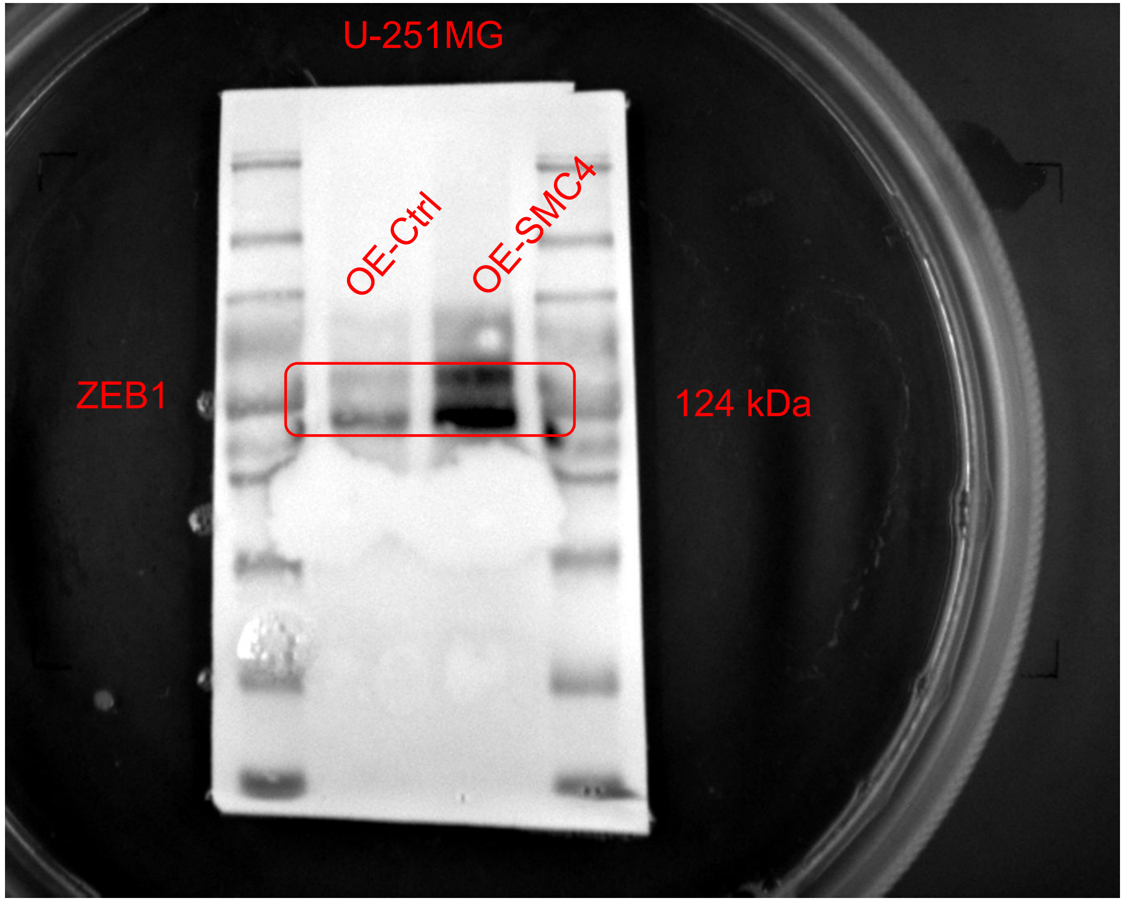
U-251MG
OE-Ctrl
OE-SMC4
ZEB1
124 kDa

Figure 4H

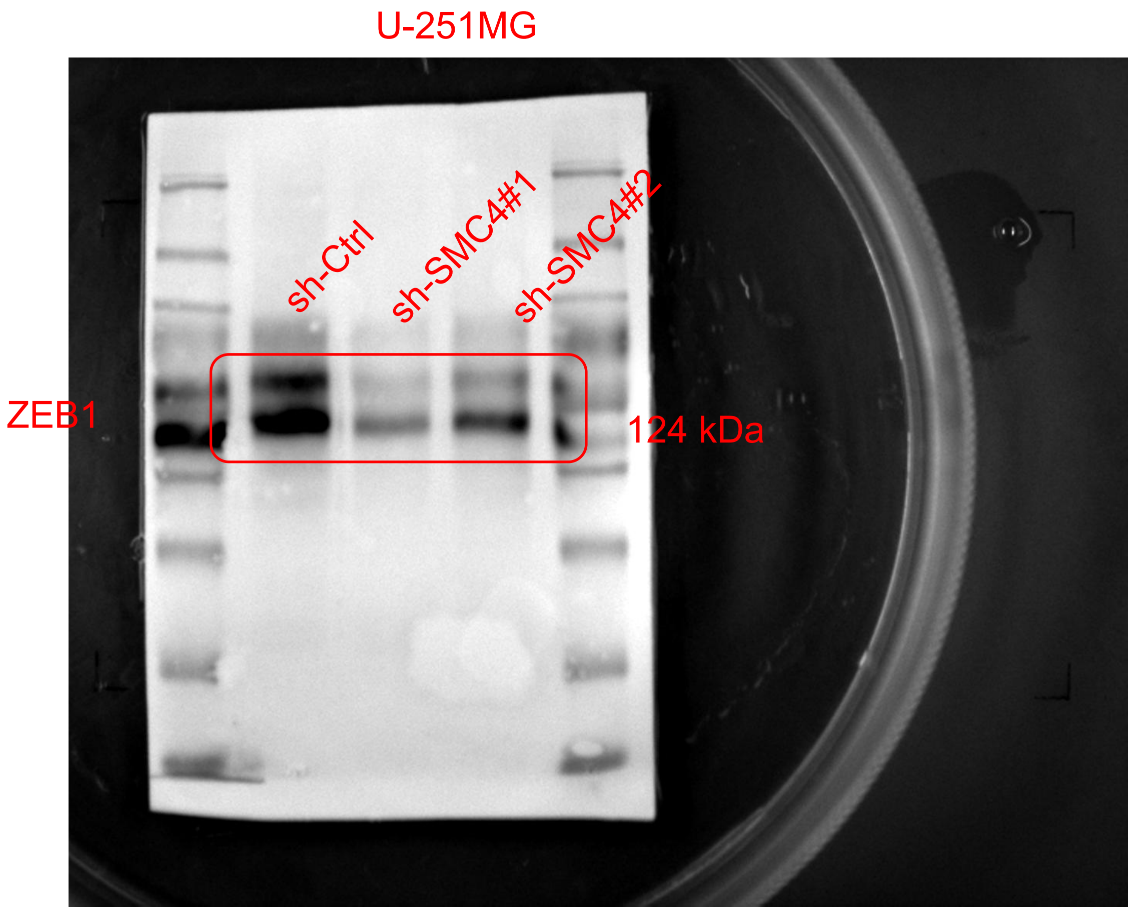

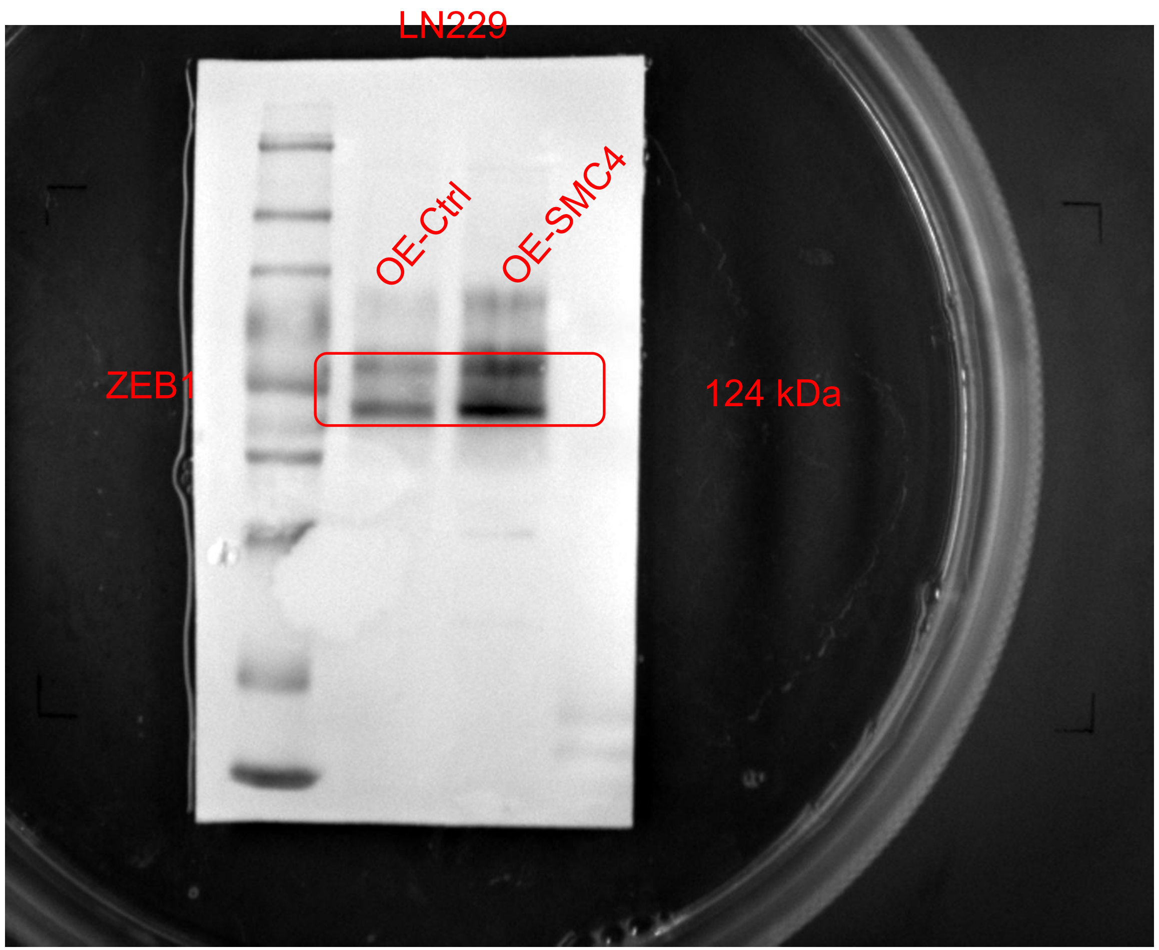
LN229
OE-Ctrl
OE-SMC4
ZEB1
124 kDa

Figure 4H

LN229

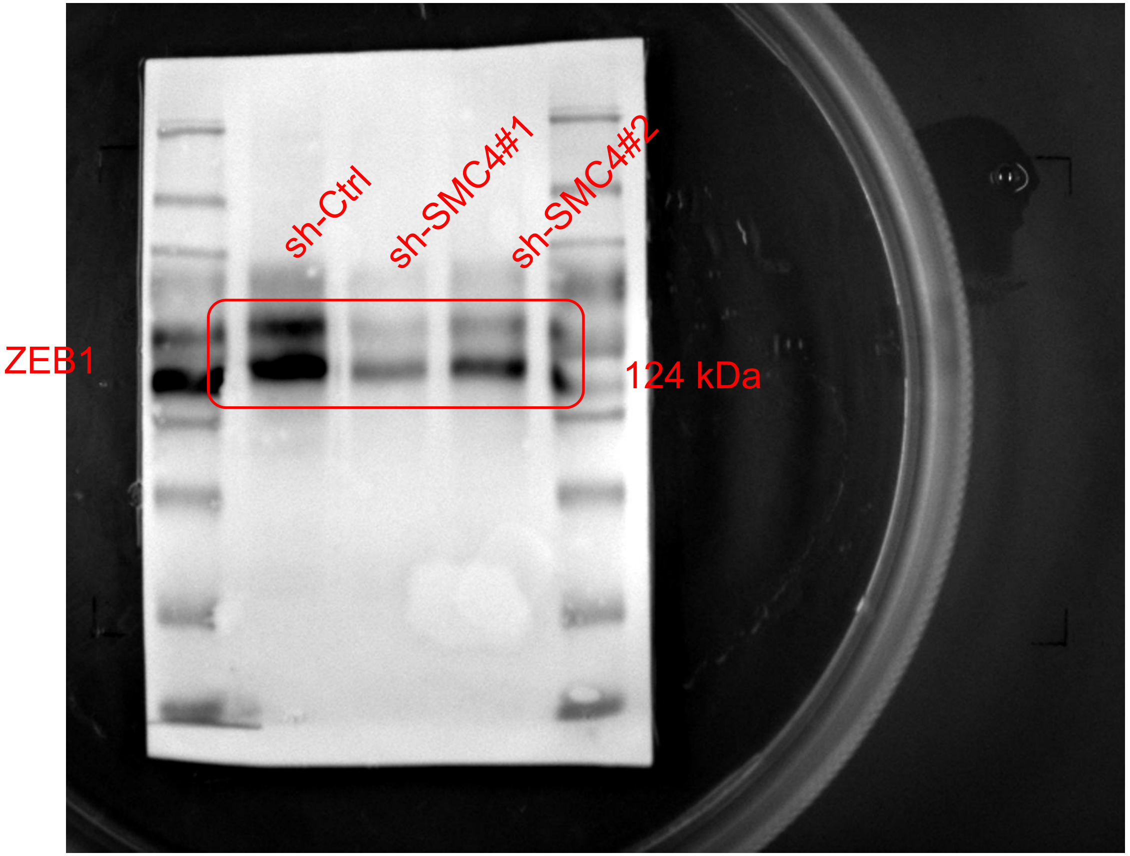

U-251MG

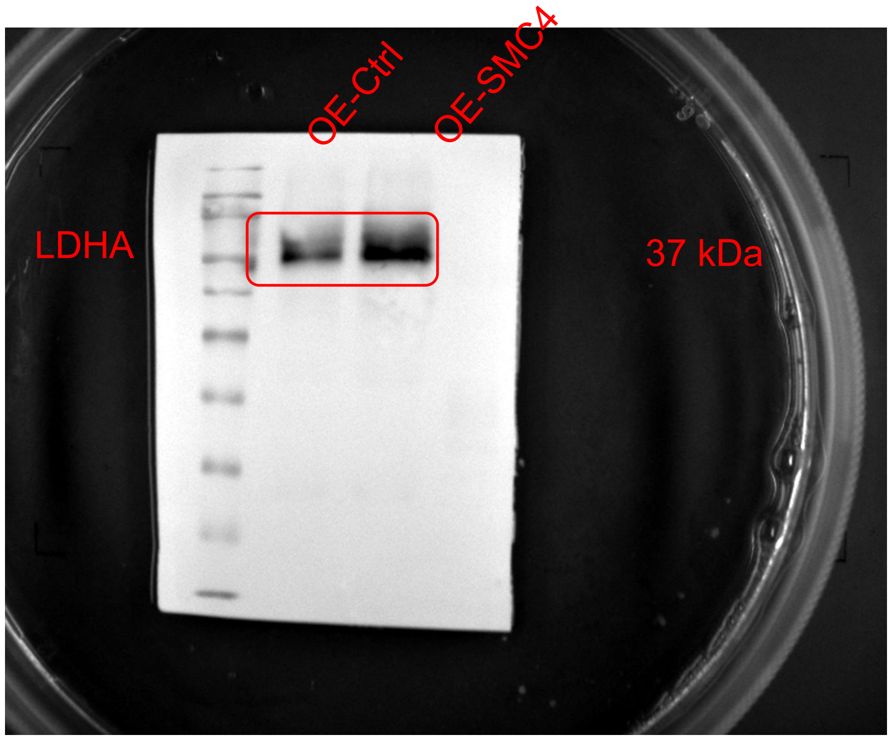

Figure 4H

U-251MG

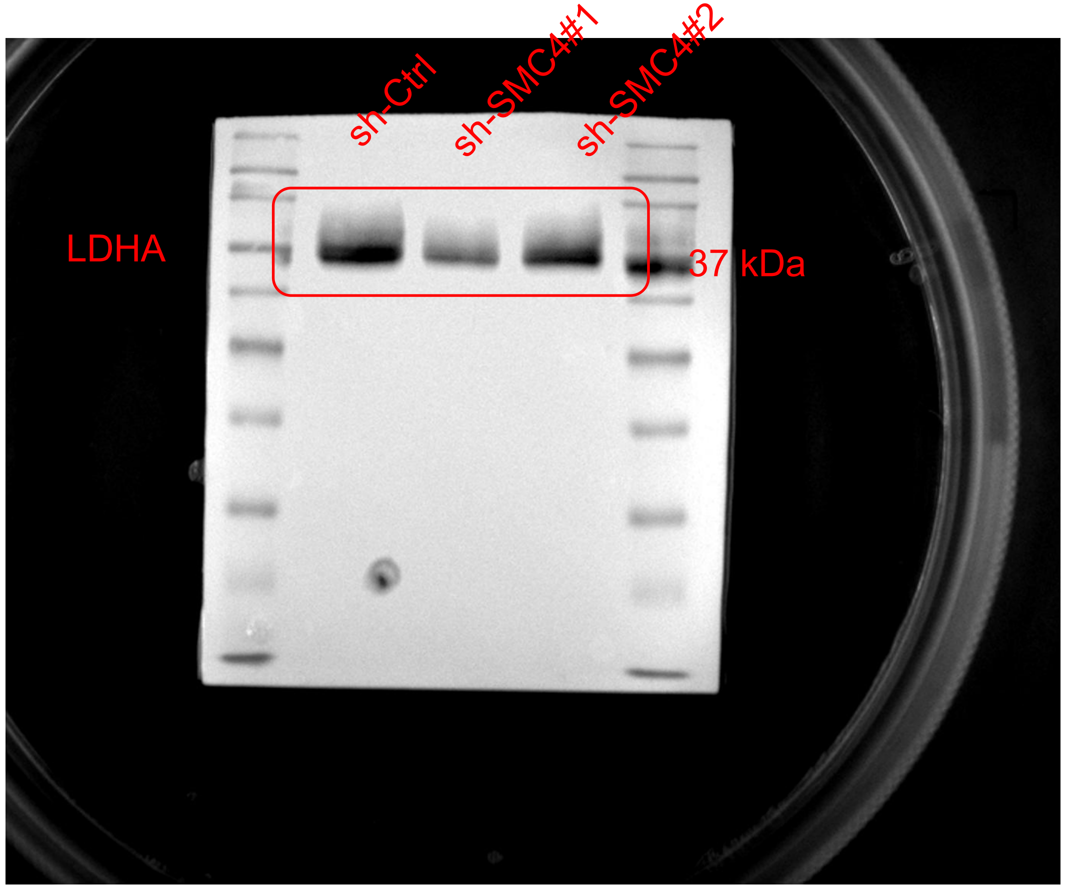

LN229

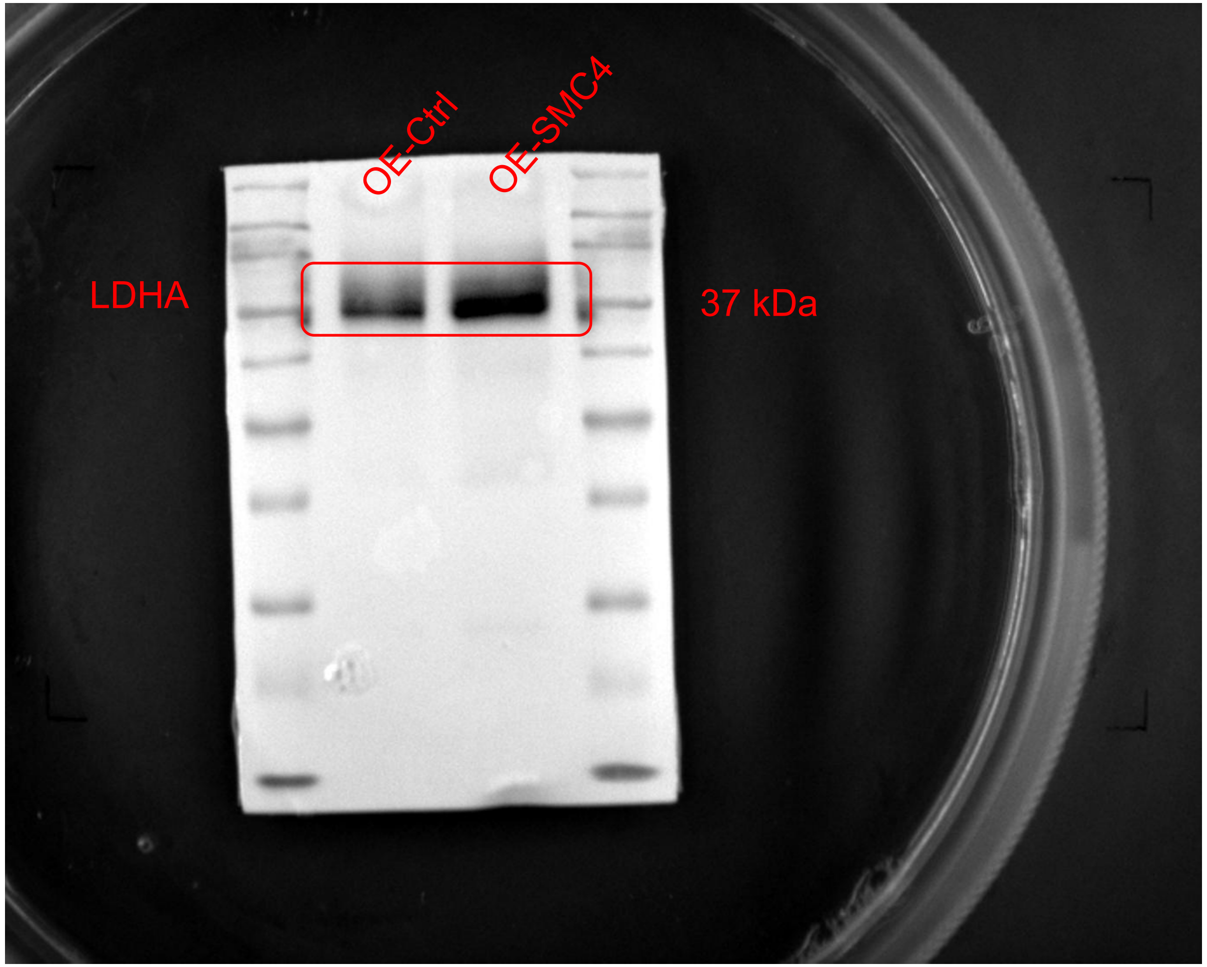

Figure 4H

LN229

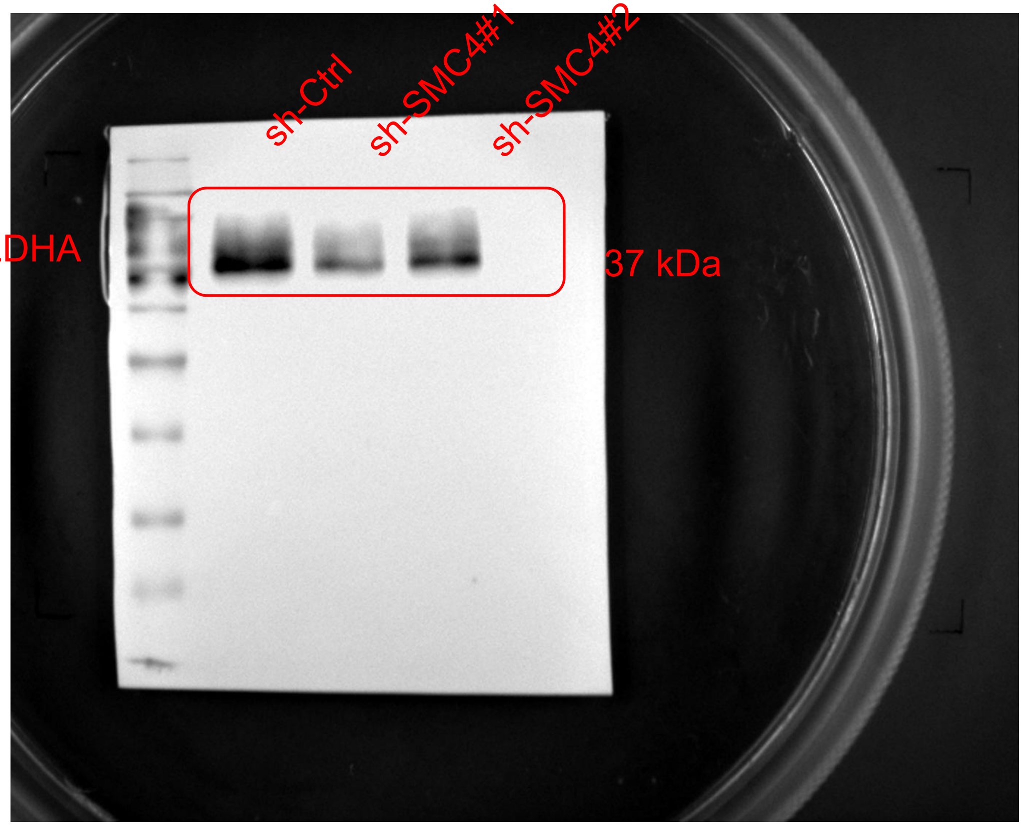

Figure 6A

U-251MG

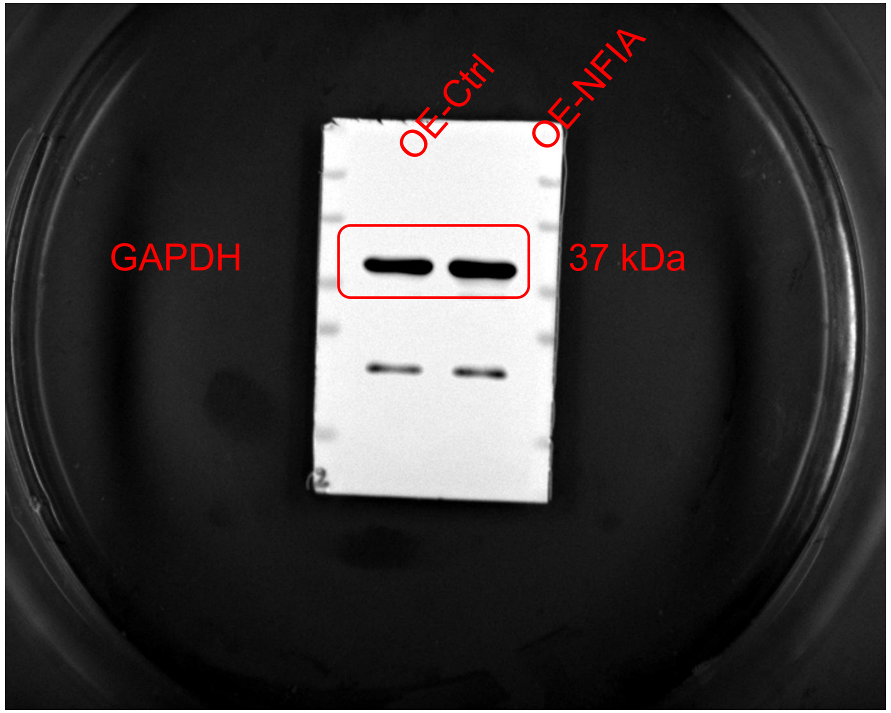

Figure 6A

U-251MG

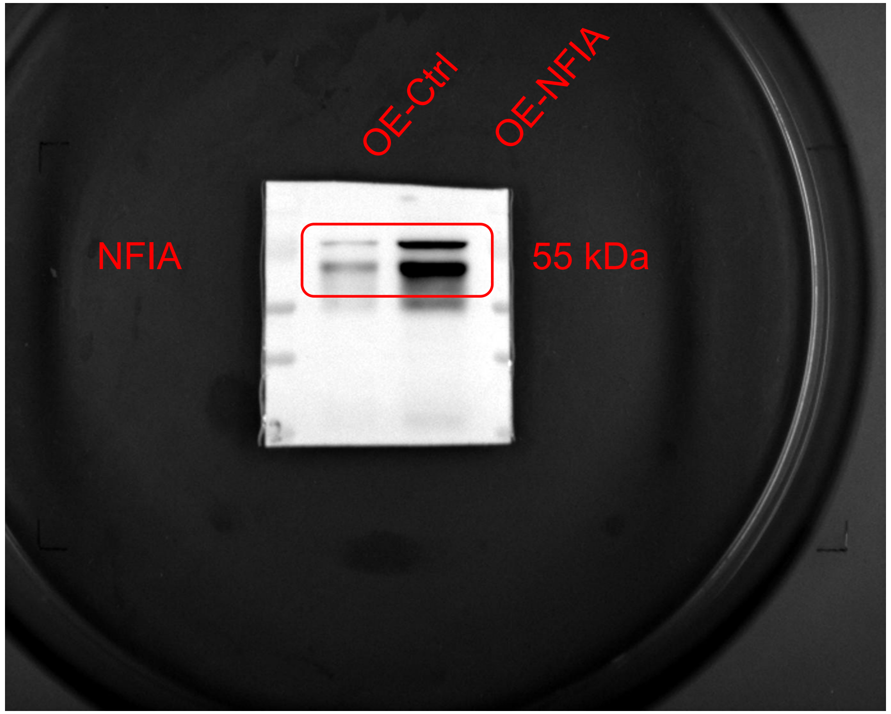

Figure 6A

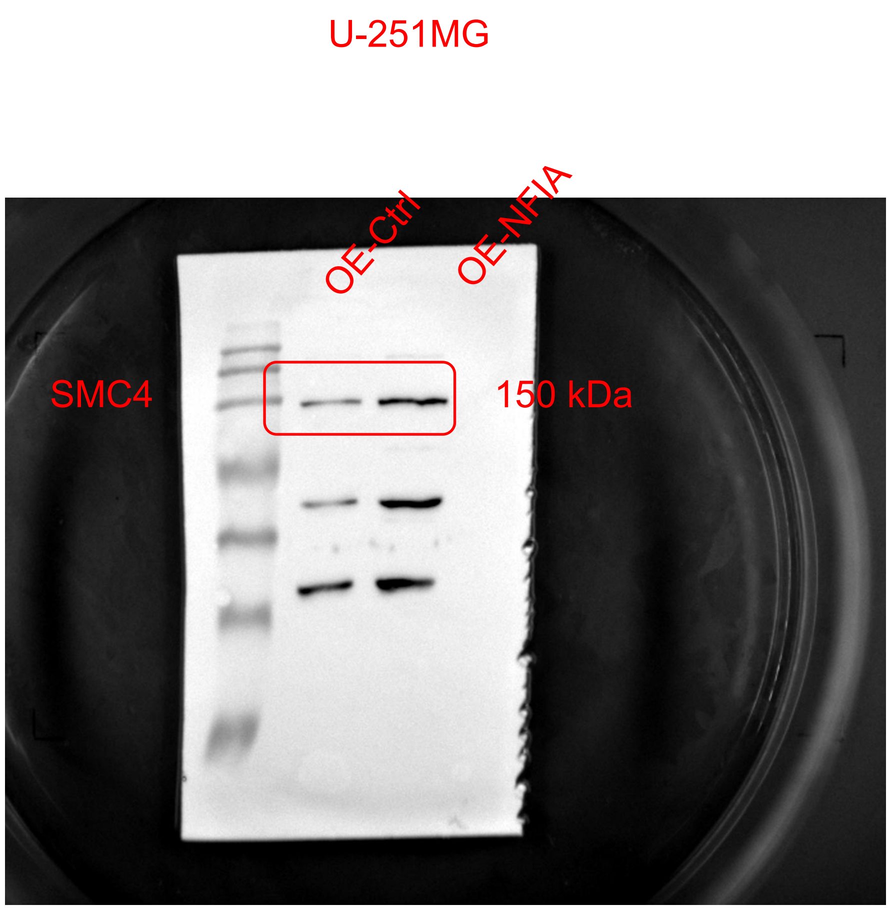

Figure 6A

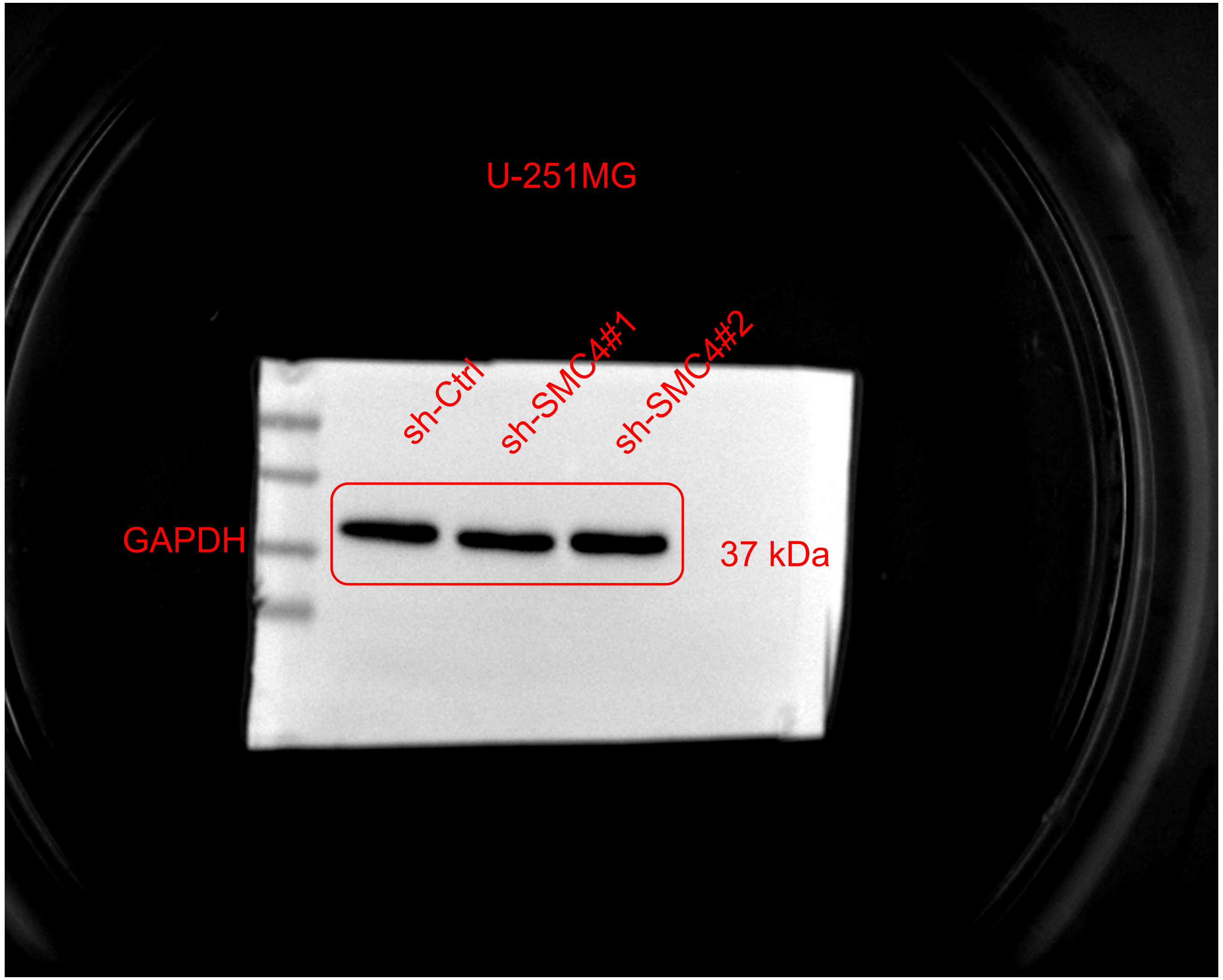

Figure 6A

U-251MG

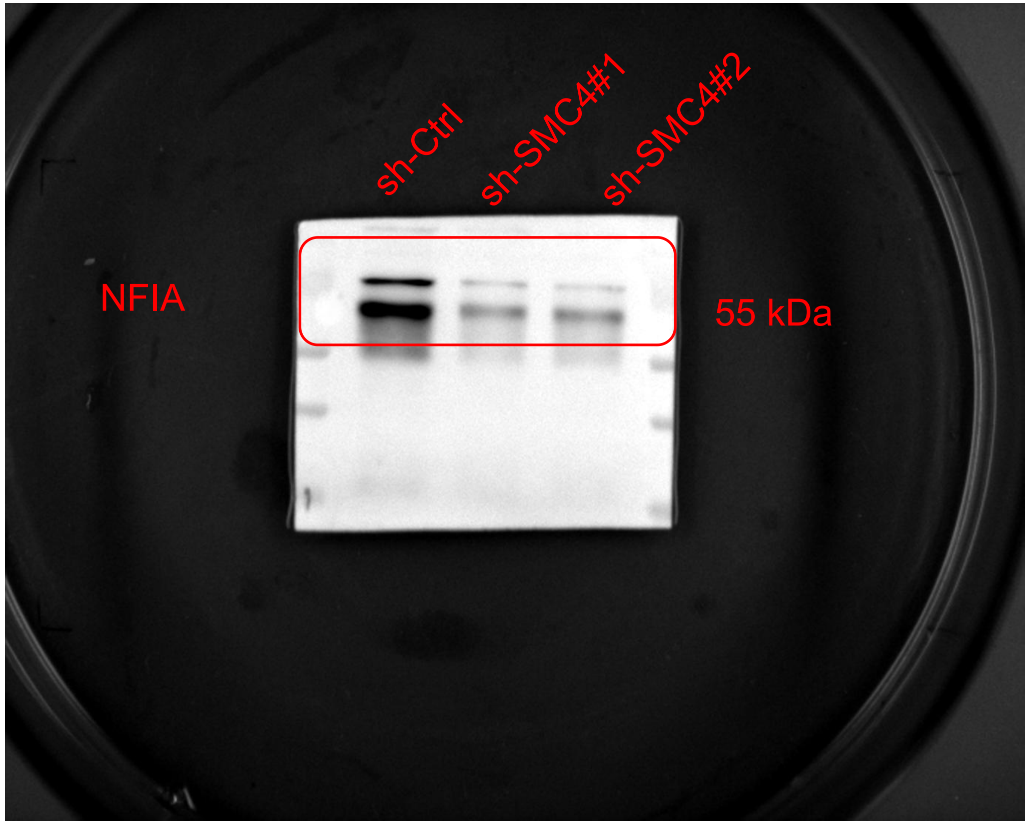

Figure 6A

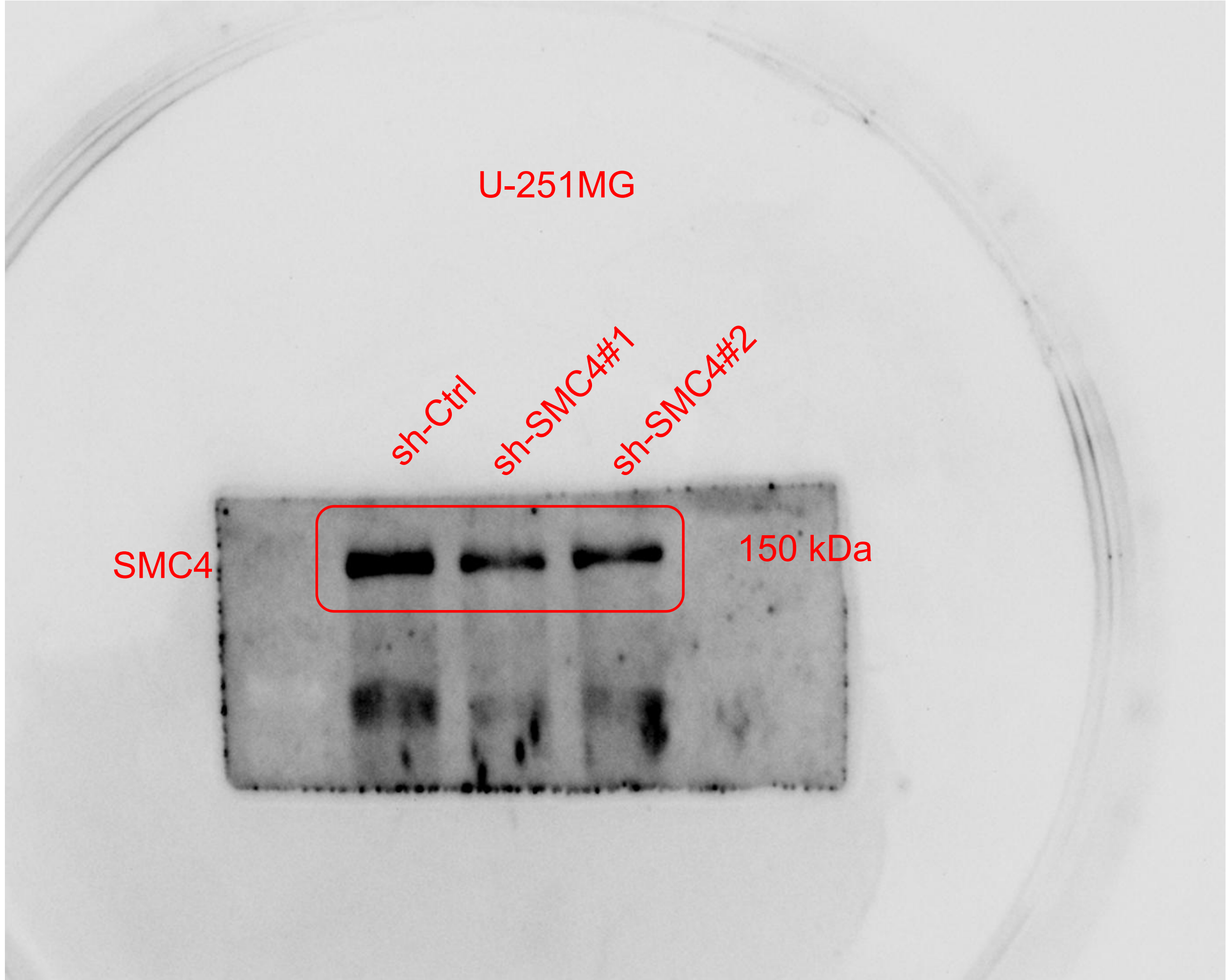

Figure 6B

LN229

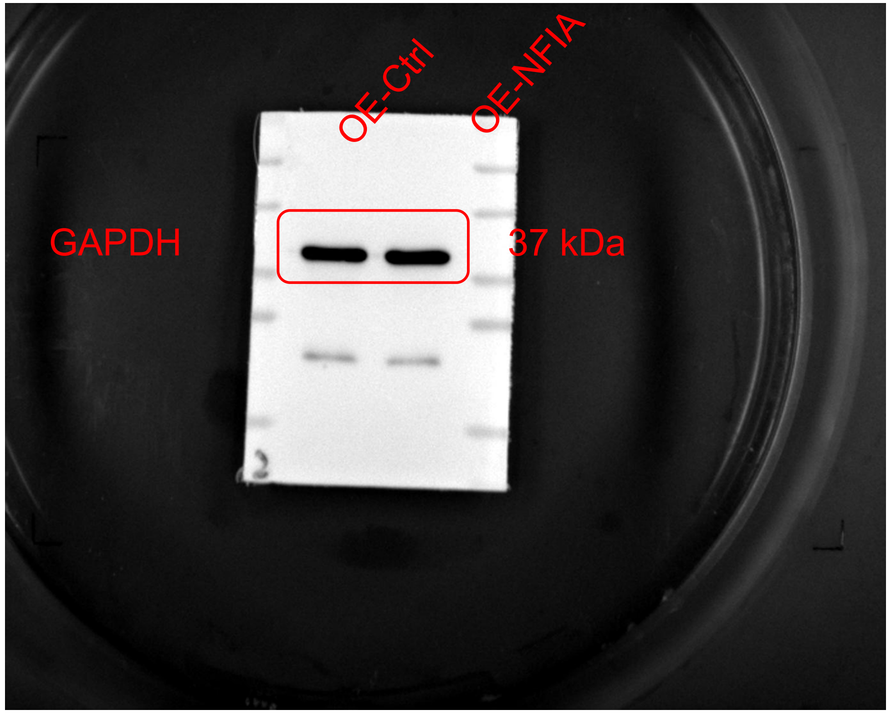

Figure 6B

LN229

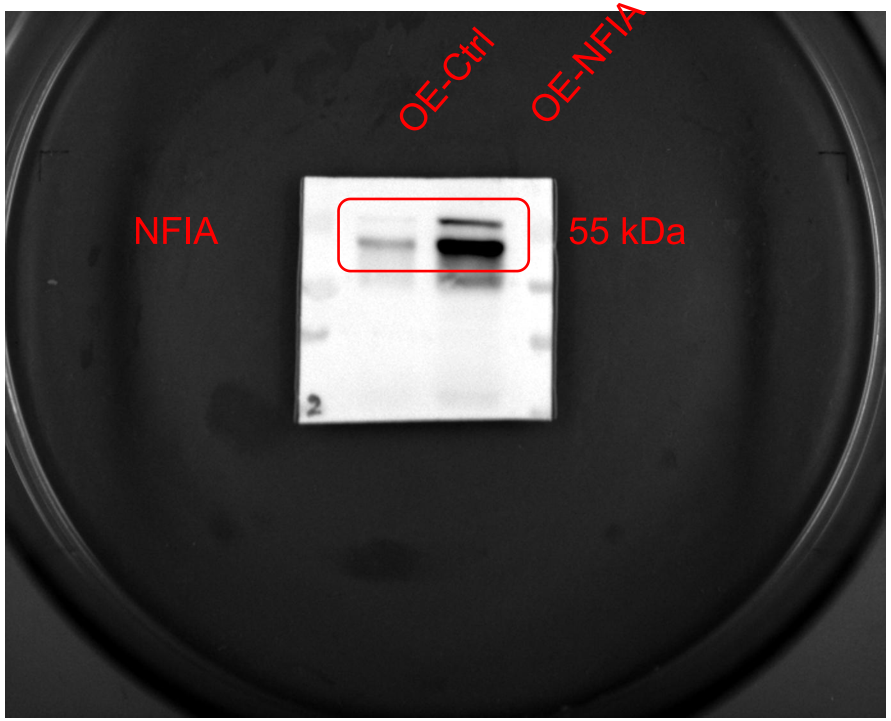

Figure 6B

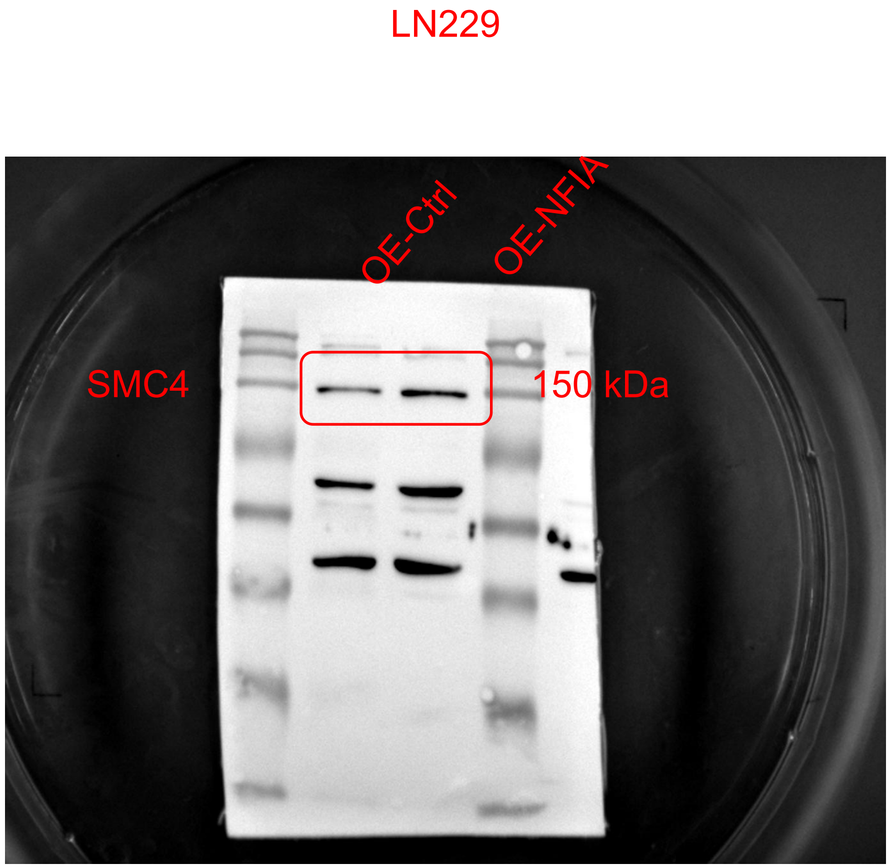

Figure 6A

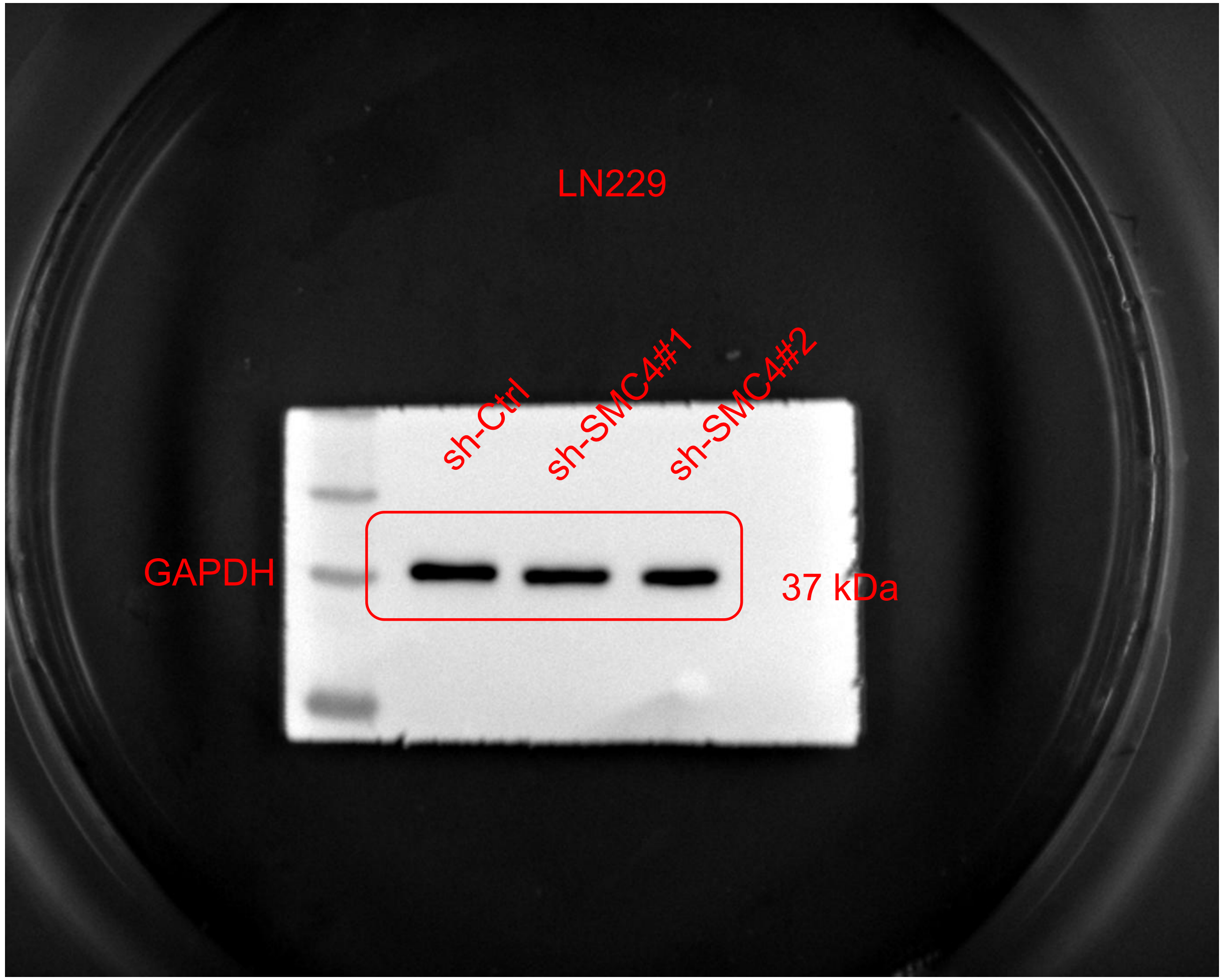

Figure 6A

LN229

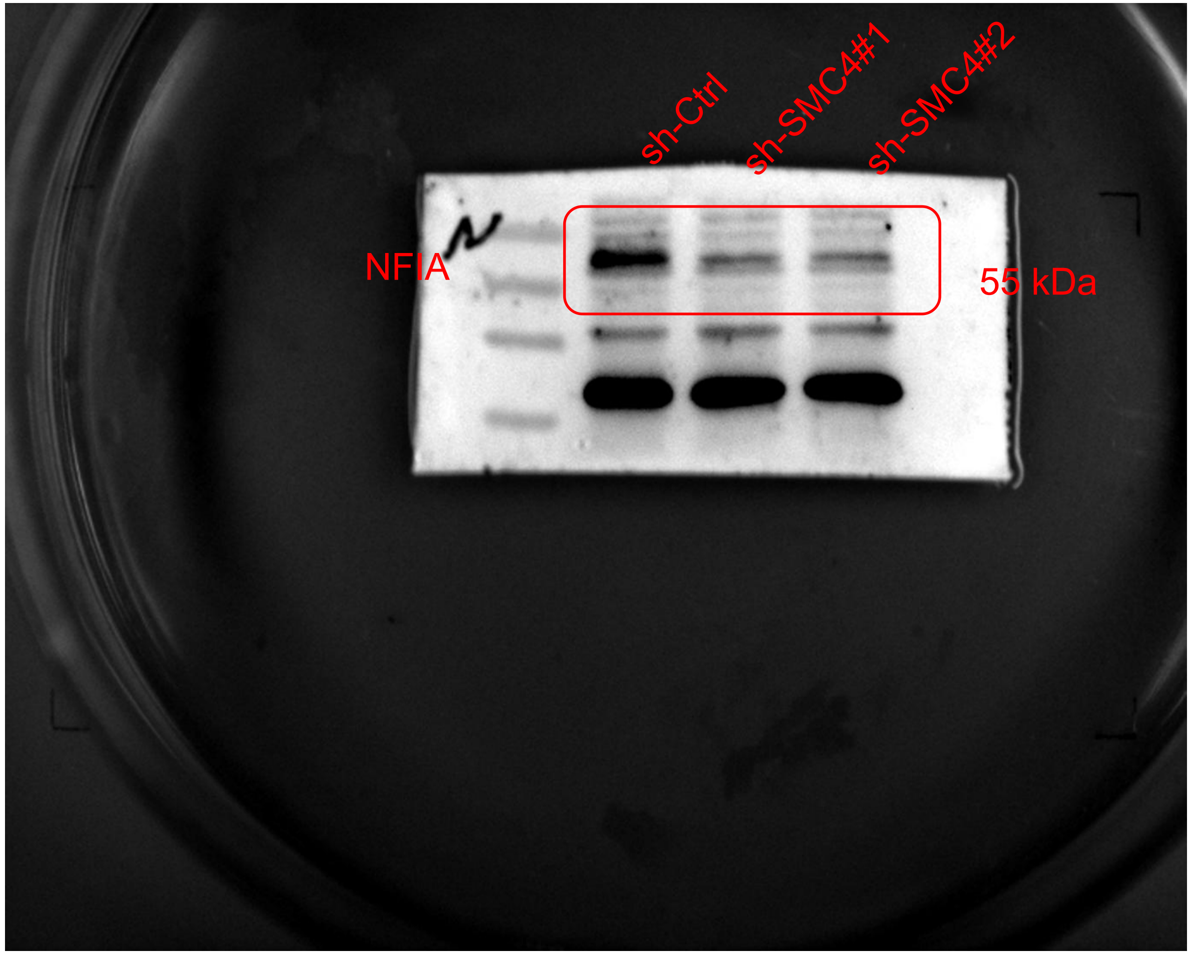

Figure 6A

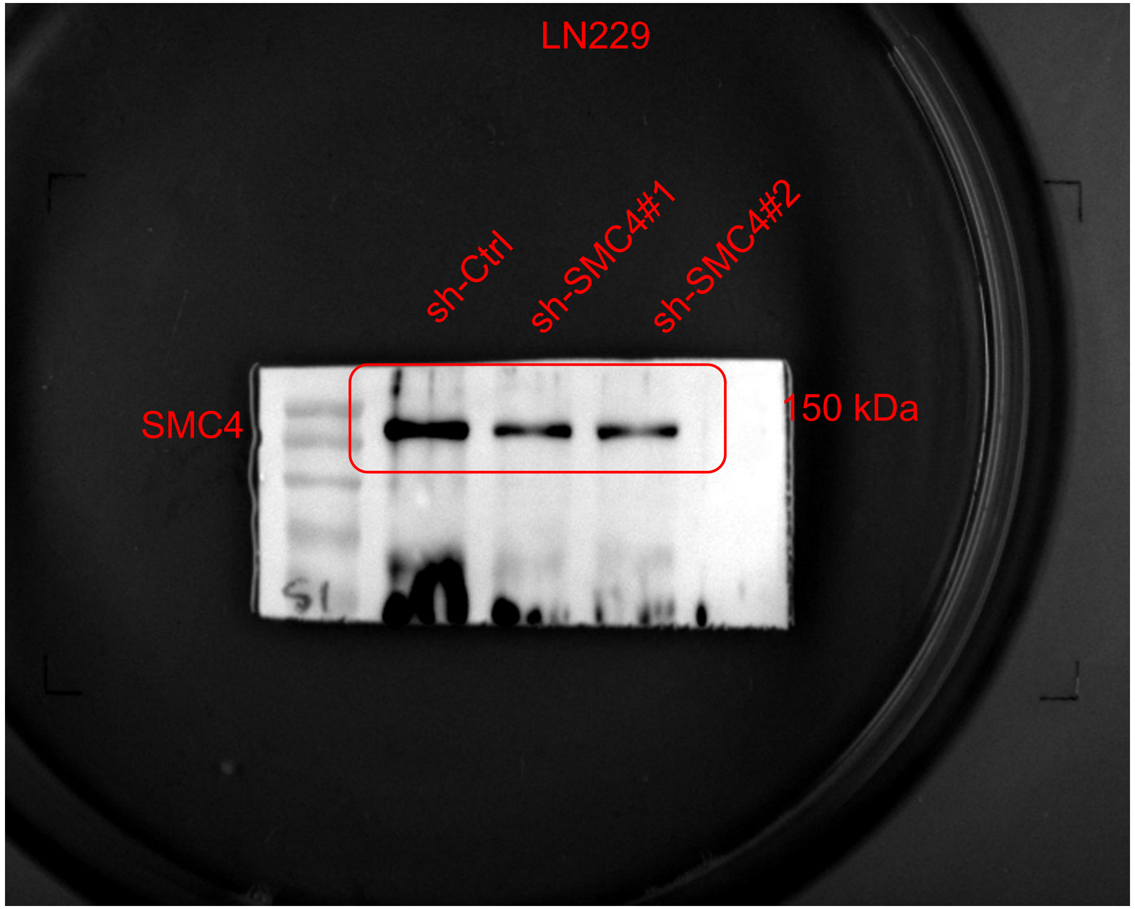

Supplement: Supplementary file 1 [file DataSheet1.pdf]
