# Supplementary material for: NFIA-dependent upregulation of SMC4 promotes metastasis and metabolic reprogramming in glioma
Source: Front Oncol. 2025 Aug 25;15:1624370. doi: 10.3389/fonc.2025.1624370 (PMC12417569; doi:10.3389/fonc.2025.1624370)
Supplement: Supplementary file 2 [file DataSheet2.docx]

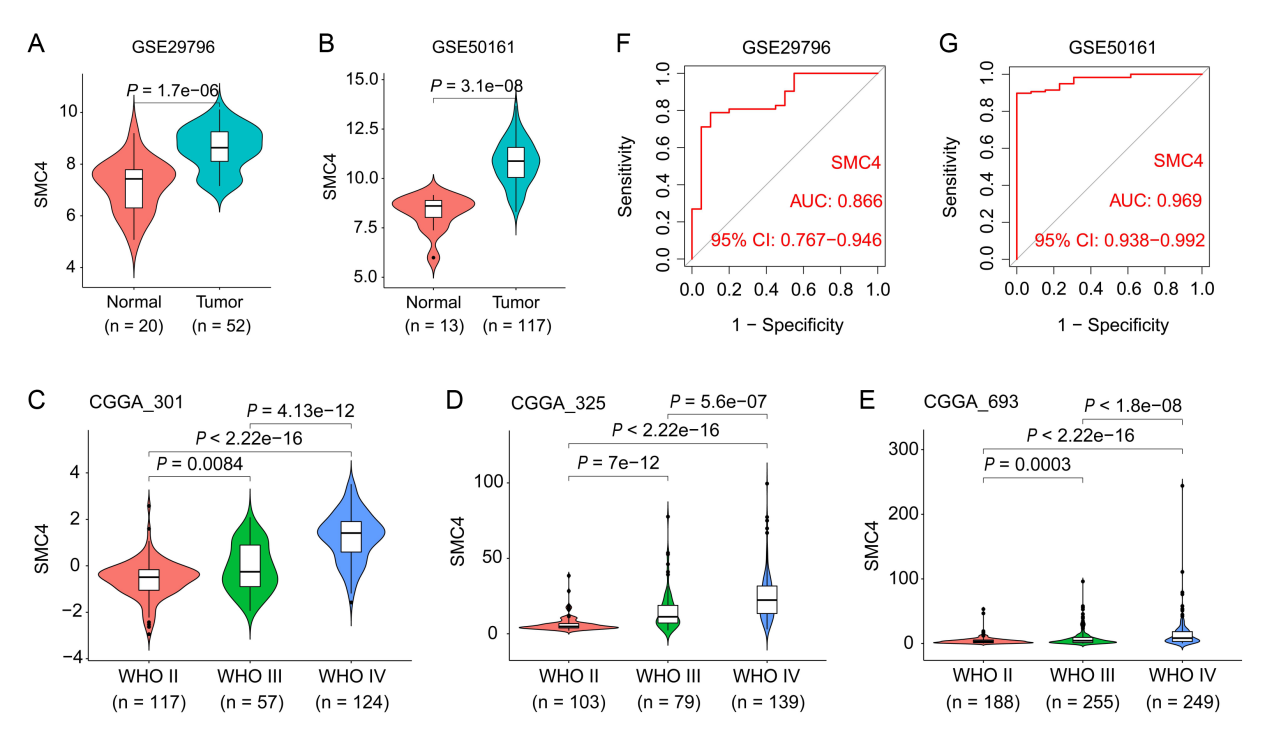


**Supplementary figure 1. SMC4 is aberrantly overexpressed in gliomas and is closely associated with gliomagenesis**

(A-B) SMC4 mRNA expression in normal brain tissues versus glioma tissues from the GSE29796 and GSE50161 datasets. (C-E) Grade-dependent SMC4 mRNA upregulation in CGGA_301, CGGA_325, and CGGA_693 cohorts. (F-G) ROC curve analysis of SMC4 expression for glioma diagnosis i GSE29796 and GSE50161 datasets.


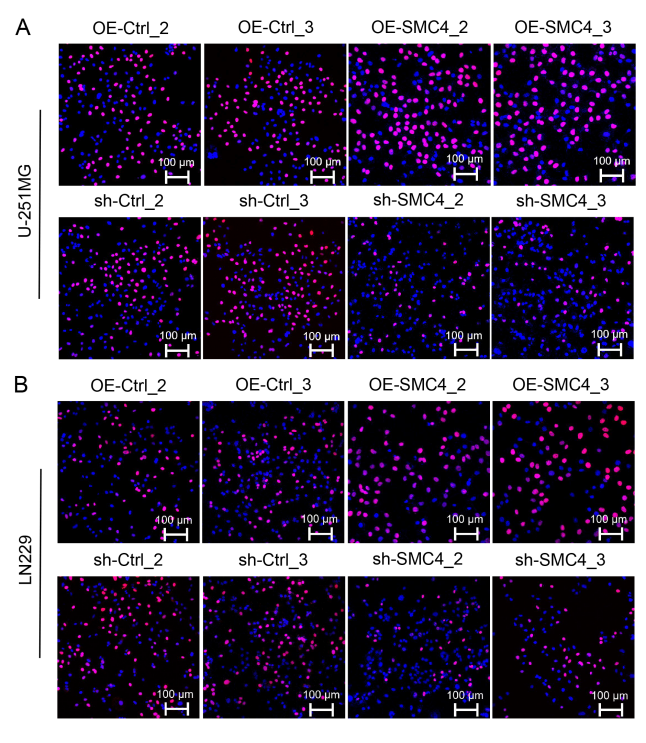


**Supplementary figure 2. EdU incorporation assay for detecting cell proliferation**

(A-B) SMC4 overexpression enhanced the positive cell rate of EdU in U-251MG and LN229, whereas knockdown reduced positive rates.


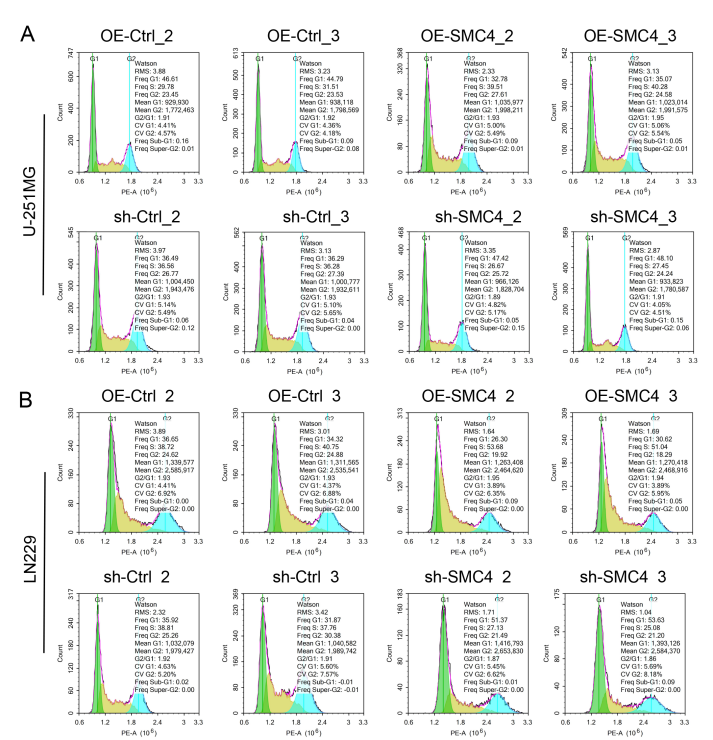


**Supplementary figure 3. Detection of cell cycle by flow cytometry**

(A-B) Flow cytometry analysis of cell cycle distribution in U-251MG and LN229 cells. SMC4 overexpression (OE-SMC4) reduced G1-phase proportion and increased S-phase accumulation, while SMC4 knockdown (sh-SMC4) exerted opposite effects. Representative flow plots.


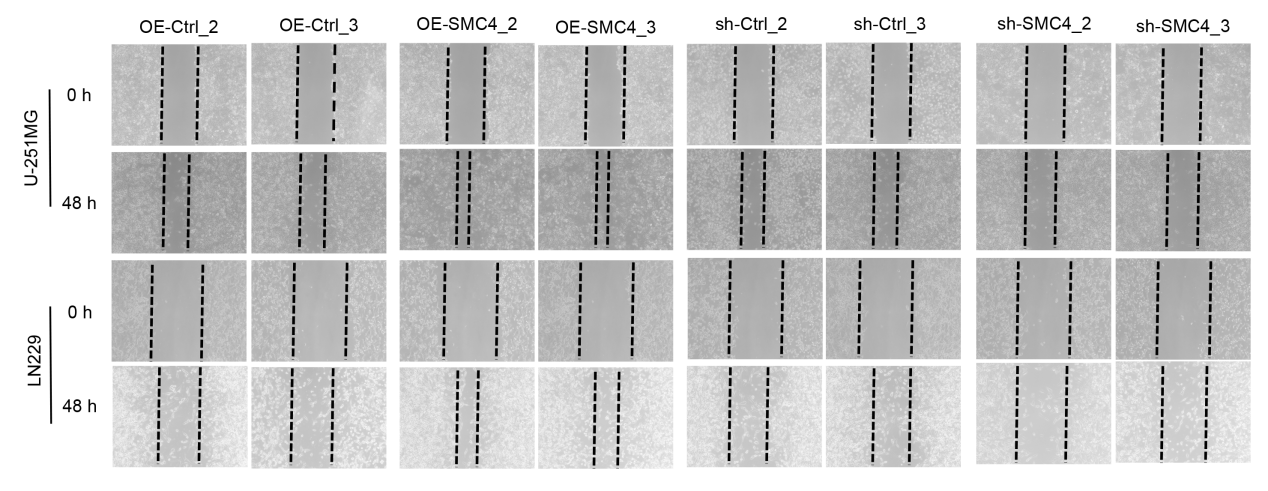


**Supplementary figure 4. Cell scratch assay to evaluate cell migration ability**

The wound-healing assay was performed to evaluate the effect of SMC4 overexpression/knockdown on the migratory capacity of U-251MG and LN229 cells. Overexpression of SMC4 promotes the migration of U-251MG and LN229 cells, while SMC4 knockdown inhibits the migration of U-251MG and LN229 cells.


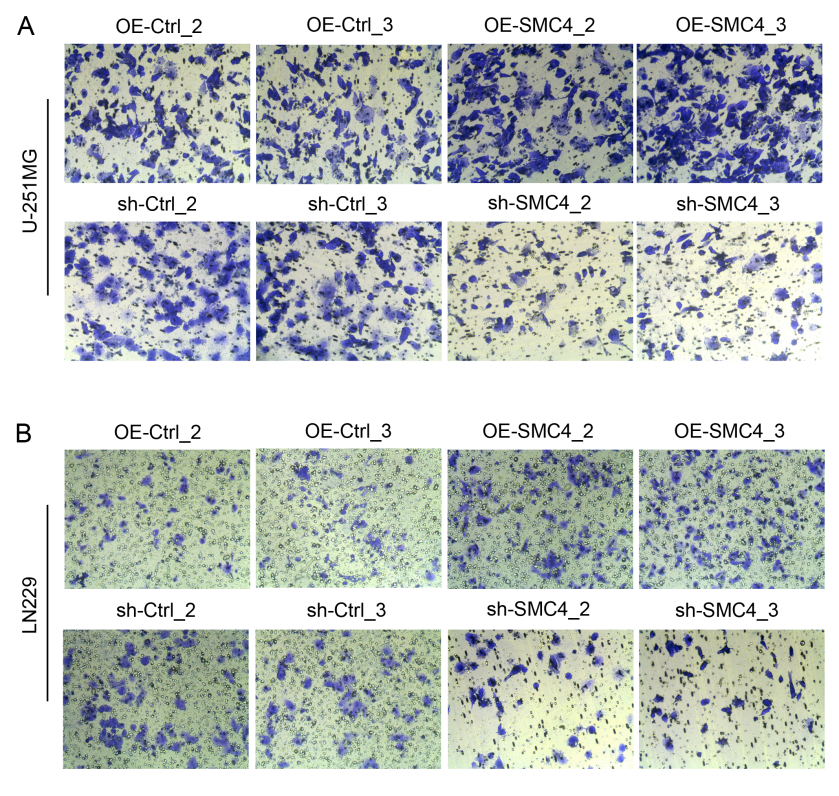


**Supplementary figure 5. Transwell assay for detecting cell invasive ability**

(A-B) The Transwell assay was conducted to examine the effects of SMC4 overexpression/knockdown on the invasive capabilities of U-251MG and LN229 cells. Overexpression of SMC4 enhances the invasive ability of U-251MG and LN229 cells, while SMC4 knockdown reduces the invasive ability of U-251MG and LN229 cells.


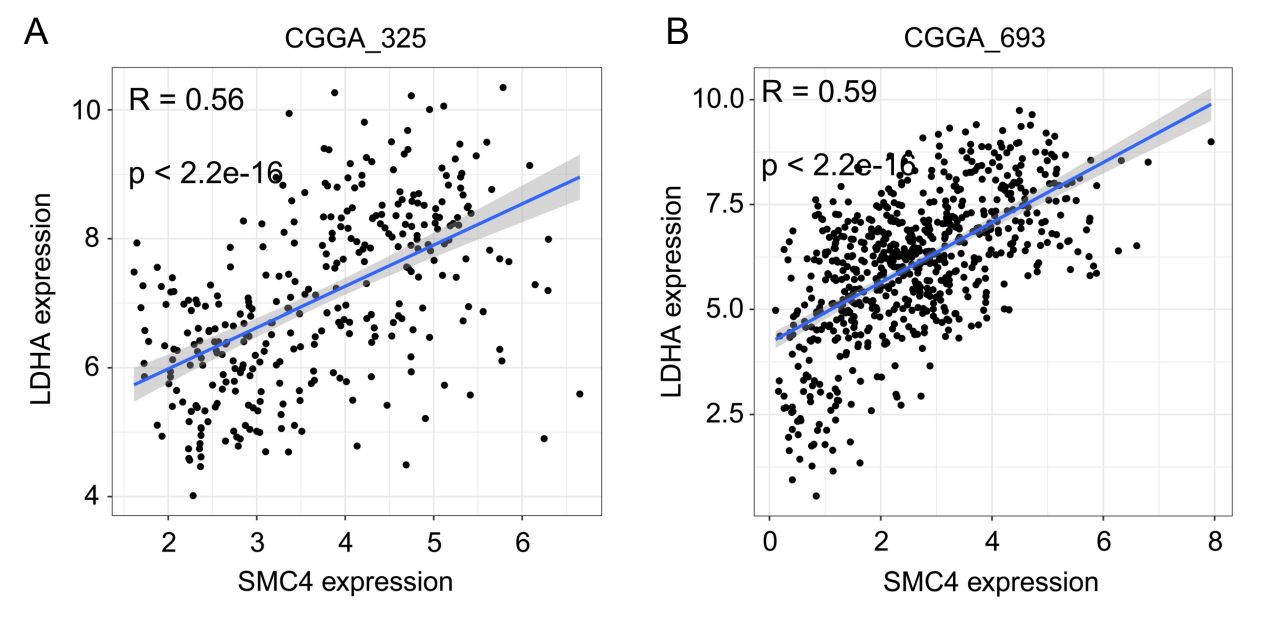


**Supplementary figure 6. Analysis of the correlation between LDHA and SMC4 expression in glioma**

(A-B) The expression levels of LDHA and SMC4 were significantly positively correlated in CGGA_325 and CGGA_693 cohorts.


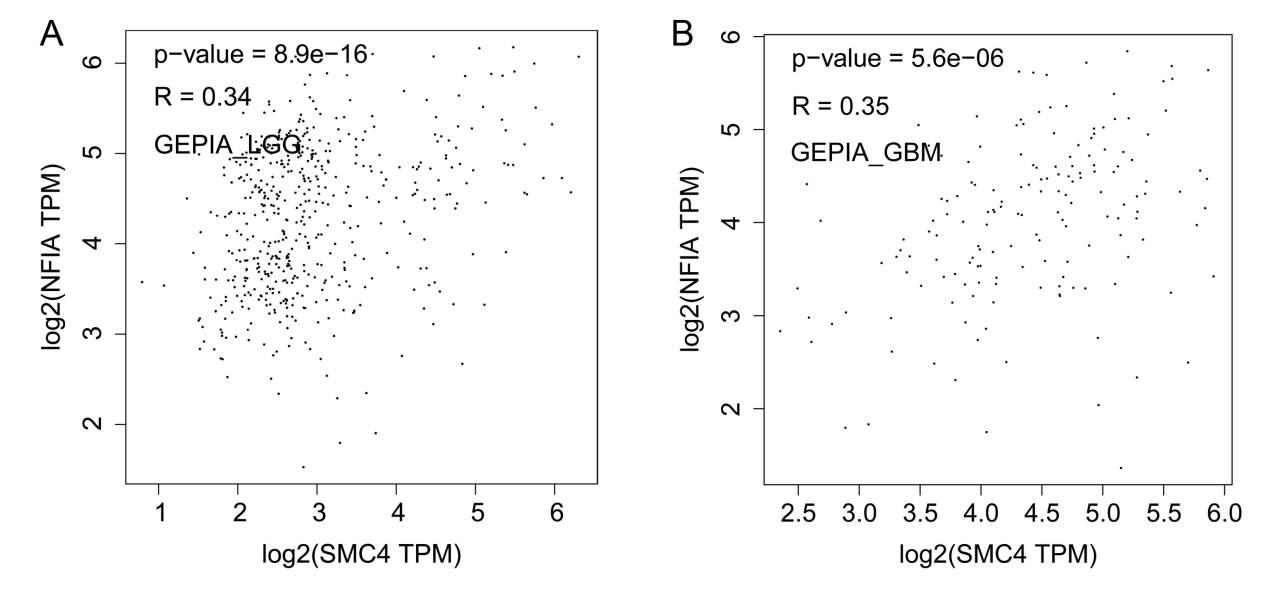


**Supplementary figure 7. Analysis of the correlation between NFIA and SMC4 expression in glioma**

(A-B) Analysis of the GEPIA database showed that the expression levels of NFIA and SMC4 were significantly positively correlated in TCGA_LGG and TCGA_GBM cohorts.


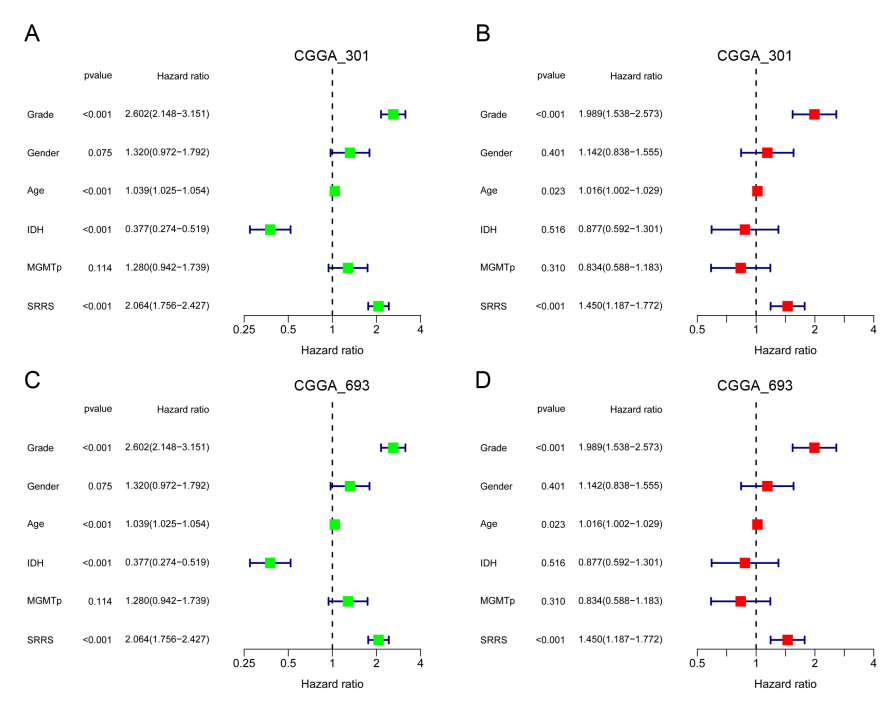


**Supplementary figure 8. Univariate and multivariate regression analyses to identify prognostic factors in patients**

(A-D) Univariate and multivariate regression analyses of the correlation between SRRS and clinical characteristics regarding OS in the CGGA_301 and CGGA_693 datasets.
